# Bmi‐1‐RING1B prevents GATA4‐dependent senescence‐associated pathological cardiac hypertrophy by promoting autophagic degradation of GATA4

**DOI:** 10.1002/ctm2.574

**Published:** 2022-04-07

**Authors:** Haiyun Chen, Jiawen Zhou, Hongjie Chen, Jialong Liang, Chunfeng Xie, Xin Gu, Rong Wang, Zhiyuan Mao, Yongjie Zhang, Qing Li, Guoping Zuo, Dengshun Miao, Jianliang Jin

**Affiliations:** ^1^ Department of Human Anatomy Research Centre for Bone and Stem Cells Key Laboratory for Aging & Disease The State Key Laboratory of Reproductive Medicine Nanjing Medical University Nanjing Jiangsu 211166 China; ^2^ The Research Center for Aging Affiliated Friendship Plastic Surgery Hospital of Nanjing Medical University Nanjing Jiangsu 210029 China; ^3^ Department of Nutrition and Food Safety School of Public Health Nanjing Medical University Nanjing Jiangsu 211166 China; ^4^ Department of Science and Technology Jiangsu Jiankang Vocational College Nanjing Jiangsu 210029 China

**Keywords:** Bmi‐1, GATA4, RING1B, selective autophagy, ubiquitination

## Abstract

**Aims:**

Senescence‐associated pathological cardiac hypertrophy (SA‐PCH) is associated with upregulation of foetal genes, fibrosis, senescence‐associated secretory phenotype (SASP), cardiac dysfunction and increased morbidity and mortality. Therefore, we conducted experiments to investigate whether GATA4 accumulation induces SA‐PCH, and whether Bmi‐1‐RING1B promotes GATA4 ubiquitination and its selective autophagic degradation to prevent SA‐PCH.

**Methods and results:**

*Bmi‐1*‐deficient (*Bmi‐1^−/−^*), transgenic *Bmi‐1* overexpressing (*Bmi‐1^Tg^*) and wild‐type (WT) mice were infused with angiotensin II (Ang II) to stimulate the development of SA‐PCH. Through bioinformatics analysis with RNA sequencing data from cardiac tissues, we found that Bmi‐1‐RING1B and autophagy are negatively related to SA‐PCH. *Bmi‐1* deficiency promoted GATA4‐dependent SA‐PCH by increasing GATA4 protein and hypertrophy‐related molecules transcribed by GATA4 such as ANP and BNP. *Bmi‐1* deficiency stimulated NF‐κB‐p65‐dependent SASP, leading to cardiac dysfunction, cardiomyocyte hypertrophy and senescence. *Bmi‐1* overexpression repressed GATA4‐dependent SA‐PCH. GATA4 degraded by Bmi‐1 was mainly dependent on autophagy rather than proteasome. In human myocardium, p16 positively correlated with ANP and GATA4 and negatively correlated with LC3B, Bmi‐1 and RING1B; GATA4 positively correlated with p62 and negatively correlated with Bmi‐1 and LC3B. With increased p16 protein levels, ANP‐, BNP‐ and GATA4‐positive cells or areas increased; however, LC3B‐positive cells or areas decreased in human myocardium. GATA4 is ubiquitinated after combining with Bmi‐1‐RING1B, which is then recognised by p62, is translocated to autophagosomes to form autophagolysosomes and degraded. Downregulated GATA4 ameliorated SA‐PCH and cardiac dysfunction by reducing GATA4‐dependent hypertrophy and SASP‐related molecules. Bmi‐1 combined with RING1B (residues 1–179) and C‐terminus of GATA4 (residues 206–443 including zinc finger domains) through residues 1–95, including a RING‐HC‐finger. RING1B combined with C‐terminus of GATA4 through the C‐terminus (residues 180–336). Adeno‐associated viral vector serotype 9 (AAV9)‐*cytomegalovirus (CMV)‐Bmi‐1‐RING1B* treatment significantly attenuated GATA4‐dependent SA‐PCH through promoting GATA4 autophagic degradation.

**Conclusions:**

Bmi‐1‐RING1B maintained cardiac function and prevented SA‐PCH by promoting selective autophagy for degrading GATA4.

**Translational perspective:**

AAV9‐*CMV*‐*Bmi‐1*‐*RING1B* could be used for translational gene therapy to ubiquitinate GATA4 and prevent GATA4‐dependent SA‐PCH. Also, the combined domains between Bmi‐1‐RING1B and GATA4 in aging cardiomyocytes could be therapeutic targets for identifying stapled peptides in clinical applications to promote the combination of Bmi‐1‐RING1B with GATA4 and the ubiquitination of GATA4 to prevent SA‐PCH and heart failure. We found that degradation of cardiac GATA4 by Bmi‐1 was mainly dependent on autophagy rather than proteasome, and autophagy agonists metformin and rapamycin could ameliorate the SA‐PCH, suggesting that activation of autophagy with metformin or rapamycin could also be a promising method to prevent SA‐PCH.

## Graphical abstract

GATA4 is ubiquitinated after combining with Bmi‐1‐RING1B, which is then recognized by p62, translocated to autophagosomes to form autophagolysosomes and degraded. Downregulated GATA4 ameliorated SA‐PCH and cardiac dysfunction by reducing GATA4‐dependent hypertrophy and SASP‐related molecules. Bmi‐1 combined with RING1B (residues 1‐179) and GATA4 (C‐terminus from residues 206 to 443 including zinc finger domains) through residues 1‐95 including a RING‐HC‐finger. RING1B combined with GATA4 (C‐terminus from residues 206 to 443 including zinc finger domains) through the C‐terminus (residues 180‐336). AAV9‐*CMV*‐*Bmi*‐1‐*RING1B* is used for gene therapy to prevent GATA4‐dependent SA‐PCH. The combined domains between Bmi‐1‐RING1B and GATA4 could be therapeutic targets for identifying stapled peptides to promote the combination of them.

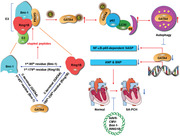

## 1 INTRODUCTION

As a key risk factor for heart failure (HF), pathological cardiac hypertrophy (PCH) results from many factors and regulators, especially renin‐angiotensin‐aldosterone system (RAAS) and its primary effector peptide angiotensin II (Ang II).^1^ Aged heart has an elevating Ang II level, and activation of RAAS is a core mechanism underlying cardiac aging in rodents.^1^, ^2^ Senescence‐associated pathological cardiac hypertrophy (SA‐PCH) is associated with the upregulation of foetal genes, fibrosis, senescence‐associated secretory phenotype (SASP), cardiac dysfunction and increased morbidity and mortality and is a prominent condition as aging becomes a growing global public health issue.^3^

GATA4 is a zinc‐finger transcription factor, which is highly expressed in embryonic and adult cardiomyocytes, classically regulating the expression levels of several structural and regulatory foetal genes, including atrial natriuretic peptide (*ANP*), brain natriuretic peptide (*BNP*) and beta myosin heavy chain (*β*‐*MHC*) at different cardiac developmental stages.^4^, ^5^ GATA4 maintains embryonic heart tube formation and cardiac development. Re‐activation of GATA4‐regulated foetal genes is considered a hallmark of PCH and HF under hypertrophic stimuli in adults.^4^, ^5^, ^6^ As a novel senescence regulator, stabilised GATA4 protein is required to activate the transcription factor NF‐κB to trigger SASP and promote aging progress.^7^, ^8^ GATA4 protein is mainly degraded by p62‐mediated selective autophagy and its inhibition prevents aging.^7^, ^8^ However, it is unclear how GATA4‐autophagic degradation is suppressed during cardiac senescence. Several lines of evidence suggest that selective autophagy decreases, causing myocardial hypertrophy and cardiac dysfunction and promoting aging.^9^, ^10^ Thus, it is urgent to study how to regulate selective autophagy of GATA4 to prevent SA‐PCH.

B cell‐specific Maloney murine leukemia virus insertion region 1 (Bmi‐1) inhibits the transcription of the *Ink4a*/*Arf* locus and maintains mitochondrial function and redox balance for avoiding cell cycle arrest and senescence.^11^, ^12^ Our previous findings demonstrate that *Bmi‐1* knockout (*Bmi‐1* ^–/–^) mice are frail and have stress‐induced premature senescence (SIPS) in multiple organs and shortened life span.^11^, ^13^, ^14^, ^15^, ^16^ We found the primary effector peptides Ang II and aldosterone (ALD) increased in plasma from *Bmi‐1* ^–/–^ mice compared to wild‐type (WT) littermates.^11^ However, whether Bmi‐1 maintains cardiac function and prevents senescence of cardiomyocytes and SA‐PCH is still unclear.

Previous studies find that the PRC1 complex formed by Bmi‐1 and RING1B is important in histone and protein ubiquitination. Bmi‐1 significantly stimulates the activity of E3 ubiquitin ligase RING1B for ubiquitination.^17^, ^18^, ^19^, ^20^ Because p62 primarily recognises ubiquitinated substrates, E3 ubiquitin ligase enzyme might be involved as a signal transducer for GATA4‐selective autophagic degradation.^21^ However, whether Bmi‐1‐RING1B promotes GATA4 ubiquitination and its selective autophagic degradation to prevent SA‐PCH is unknown.

This study demonstrated that Bmi‐1‐RING1B promoted GATA4 ubiquitination and its selective autophagic degradation to prevent SA‐PCH. We also clarify the combined domains between Bmi‐1‐RING1B and GATA4. This signal in aging cardiomyocytes could be a therapeutic target for identifying stapled peptides to promote the combination of Bmi‐1‐RING1B with GATA4 and the ubiquitination of GATA4 to prevent SA‐PCH and HF. Adeno‐associated viral vector serotype 9 (AAV9)‐ *cytomegalovirus (CMV)‐Bmi‐1‐RING1B* treatment significantly ameliorated GATA4‐dependent SA‐PCH through promoting GATA4 autophagic degradation, which could be used for translational gene therapy.

## 2 MATERIALS AND METHODS

Please see the detail in Supplementary Information 4.

### 2.1 High‐throughput sequencing analysis

The gene expression profile data comparing cardiac tissues from young and aging mice were downloaded from Gene Expression Omnibus (GEO) database (https://www.ncbi.nlm.nih.gov/geo/). GSE161078 contains the mRNA expression data of whole hearts from 14‐week‐old, 12‐month‐old and 18‐month‐old mice.

Please see the detail for analysis in Supplementary Information 4.

### 2.2 Animals

The *Bmi‐1* heterozygous (*Bmi‐1* ^+/−^) mouse line with a C57BL/6J background was from McGill University.^13^ *Bmi‐1* overexpression in mice with a C57BL/6J background under the control of the 2.4‐kb *Prx1* promoter (*Bmi‐1^Tg^*) was generated at Nanjing Medical University (Nanjing, China).^22^, ^23^ All experiments on animals followed the guidelines for the Care and Use of Laboratory Animals published by the US National Institutes of Health (NIH Publication, 8th Edition, 2011) and uses of mice were approved by the Institutional Animal Care and Use Committee of Nanjing Medical University (Permit Number: IACUC‐1706001).

For chronic induction of cardiac hypertrophy, male mice were anesthetised with 0.02 g/ml pentobarbital sodium (50 mg/kg body weight, intraperitoneal injection) and implanted with ALZET® Micro‐Osmotic Pumps (Model 1004) (#10370‐16, DURECT Corporation, CA, USA) in the subcutaneous skin on the back for infusing angiotensin II (Ang II, #A9525, Sigma‐Aldrich, St. Louis, MO, USA), and the dose was 1.3 mg/kg/day for 4 weeks. For tissue collection, mice were anaesthetised using overdose anesthesia (50 mg/kg body weight, intraperitoneal injection) and then sacrificed by cervical dislocation and hearts were used for *ex vivo* experiments.

### 2.3 Cell line from adult human ventricular cardiomyocytes

Human AC16 cells (BeNa Culture Collection, Beijing, China) were cultured in medium including 90% DMEM/F12, 10% FBS (Gibco) and supplemented with 100 U/ml penicillin and 100 μg/ml streptomycin as previously described.^24^, ^25^ Ang II (1 × 10^–5^ for 70 h) was used to induce hypertrophy of AC16 cells.

### 2.4 Mouse embryonic cardiomyocytes cultures

Please see the detail in Supplementary Information 4.

### 2.5 Samples of human myocardium tissues

Human heart tissue samples were collected from 25 autopsied donors at the Department of Human Anatomy at Nanjing Medical University. This research complies with ethical guidelines of the 1975 Declaration of Helsinki. Anatomical methods and all experiments^16^ were approved by the Committee on the Ethics of Nanjing Medical University (Permit Number: 2019–902).

### 2.6 Colour Doppler echocardiography

The ultrasound imaging system (Vevo 2100, VisualSonics, Toronto, Canada) with high frequency was used to measure as previously described.^26^

### 2.7 Plasma Ang II and aldosterone

Ang II and aldosterone (ALD) levels in plasma were detected using radioimmunoassay kits (Beijing North Institute of Biological Technology, Beijing, China).^11^

### 2.8 Western blots

Primary antibodies were against p19 (NB200‐106, Novus Biologicals, CO, USA), p21 (#2947, Cell Signaling Technology, Beverly, MA, USA), p53 (#2424, Cell Signaling Technology, USA), p16 (ab211542, Abcam, Cambridge, MA, USA), renin (#5250, Cell Signaling Technology, USA), Ang II (NBP1‐31127, Novus Biologicals, USA), ANP (#AB5490, Millipore, MA, USA; sc‐515701, Santa Cruz Biotechnology Inc., Dallas, TX, USA, USA), BNP (#DF6902, Affinity Biosciences, OH, USA; ab19645, Abcam, USA), GATA4 (#19530, Proteintech, IL, USA; sc‐25310, Santa Cruz Biotechnology Inc., USA), LC3B (#NB600‐1384, Novus Biologicals, USA), p62 (#39749, Cell Signaling Technology, USA), Bmi‐1 (#5856, Cell Signaling Technology, USA; 66161‐1‐Ig, Proteintech, USA), RING1B (#5694, Cell Signaling Technology), NF‐κB‐p65 (sc‐8008, Santa Cruz Biotechnology Inc., USA; #8242, Cell Signaling Technology, USA), p‐p65 (Ser536) (ab76302, Abcam, USA), IκB‐α (AF1282, Beyotime Biotechnology, Shanghai, China), p‐IκB‐α (Ser32) (sc‐8404, Santa Cruz Biotechnology Inc., USA), p‐Chk2 (Thr68) (PA5‐104715, Invitrogen Inc. CA, USA), SOD‐2 (NB100‐1992, Novus Biologicals, USA), HSC70 (10654‐1‐AP, Proteintech, USA), p‐ULK1 (Ser757) (#14202, Cell Signaling Technology, USA) and ULK1 (sc‐390904, Santa Cruz Biotechnology, USA). β‐actin (AP0060, Bioworld Technology Inc., MN, USA) was loading control for total protein.

### 2.9 RNA extraction and real‐time RT‐PCR

The TRIzol reagent (#15596, Invitrogen Inc., USA) was used to extracted total RNAs from hearts or cells. The mRNA expression levels were quantitatively detected as described.^16^ Primers are listed in Table S1 (Supplementary Information 5).

### 2.10 Histology

Heart samples were prepared as previously described.^11^, ^14^ Sections were made and used for histochemical staining including immunohistochemistry, haematoxylin and eosin (H&E), Masson's trichrome (Masson) and wheat germ agglutinin (WGA).

#### 2.10.1 Immunohistochemical staining

Primary antibodies were against ANP (#AB5490, Millipore, USA; sc‐515701, Santa Cruz Biotechnology Inc., USA), BNP (#DF6902, Affinity Biosciences, USA; ab19645, Abcam, USA), GATA4 (#19530, Proteintech, USA), 8‐OHdG (ab62623, Abcam, USA), γH2A.X (#9718, Cell Signaling Technology, USA), NF‐κB‐p65 (#8242, Cell Signaling Technology, USA), IL‐1β (sc‐52012, Santa Cruz Biotechnology Inc., USA), IL‐6 (sc‐1265, Santa Cruz Biotechnology Inc., USA) or TNF‐α (NBP1‐19532, Novus Biologicals, USA).

#### 2.10.2 Immunofluorescent staining

Primary antibodies were against GATA4 (#19530, Proteintech, USA), GATA4 (sc‐25310, Santa Cruz Biotechnology Inc., USA), LC3B (#NB600‐1384, Novus Biologicals, USA), p62 (#39749, Cell Signaling Technology, USA) or LAMP2 (sc‐20004, Santa Cruz Biotechnology Inc., USA).

#### 2.10.3 Masson's trichrome staining

The Masson’s trichrome staining kit (#KGMST‐8003, KeyGen Biotech Co. Ltd., Nanjing, Jiangsu, China) was used.^16^

#### 2.10.4 Wheat germ agglutinin staining (WGA)

WGA conjugated to Alexa Fluor 488 (MP00831, Molecular Probes Inc., OR, USA) was used.^1^

#### 2.10.5 SA‐β‐gal staining

Cells were fixed and stained using a cell senescence β‐galactosidase staining kit (#C0602, Beyotime Institute of Biotechnology, Shanghai, China).^16^

### 2.11 Autophagy detection lentivirus

#### 2.11.1 Autophagy lentivirus infection

Autophagy lentivirus (#HB‐LP210 0001) was from HanBio Technology Co., Ltd. in Shanghai of China and used as previously described.^27^ Its fusion protein elements included mCherry red fluorescent protein (mRFP), green fluorescent protein (GFP) and autophagy marker protein LC3B (mRFP‐GFP‐LC3) to monitor formation of autophagosomes and autolysosomes.

#### 2.11.2 Overexpression and knockdown lentivirus infection

The *Bmi‐1* overexpression lentivirus and knockdown lentivirus were designed and synthesised by Genechem Co., Ltd. in Shanghai of China. Please see the details of infection in Supplementary Information 4.

### 2.12 Intracellular reactive oxygen species analysis

2′, 7′‐Dichlorofluorescein diacetates (DCFDA) (D399, Invitrogen Inc., USA) was used at 5 mM.^11^, ^12^

### 2.13 Enzyme‐linked immunosorbent assay

Concentrations of Bmi‐1 (#H00115), RING1B/RNF2 (#H00515), p16 (#H01273), GATA4 (#H00555), ANP (#H0470), p62/SQSTM1 (#H00597) and LC3B (#H00815) in human myocardial tissue were detected using enzyme‐linked immunosorbent assay (ELISA) kits from Yifeixue Biotechnology in Nanjing of China.^16^

### 2.14 Protein sequence alignment

The amino acid sequences of GATA3, GATA4 and GATA6 protein from mouse or human were aligned using online tools (Uniprot/Align: https://www.uniprot.org/align/) (Supplementary Information 6 and 7).

The amino acid sequences of GATA4 protein and motif ‘KFERQ’ from mouse or human were aligned using online tools (Uniprot/Align: https://www.uniprot.org/align/) (Supplementary Information 8).

### 2.15 Duolink proximity ligation assay

Antibodies against GATA4 (sc‐25310, Santa Cruz Biotechnology Inc., USA), Bmi‐1 (#5856, Cell Signaling Technology), Bmi‐1 (AM1930b, ABCEPTA Inc., CA, USA), RING1B (#5694, Cell Signaling Technology) or HSC70 (10654‐1‐AP, Proteintech, USA) was used. PLA in situ fluorescence (Sigma‐Aldrich, USA) was conducted according to the manufacturer's instructions.^16^

### 2.16 Plasmid construction and transfection of truncated and full‐length fragments

All of plasmids were produced by TranSheep Bio Co. Ltd in Shanghai of China.

Based on the structural features of Bmi‐1, we generated three truncated fragments containing a RING‐HC‐finger, WD40‐associated ubiquitin‐like (RAWUL) domain and carboxy‐terminal domain of human Bmi‐1, all with a His‐tag in pmCherry‐C1 vector plasmid. We generated a *Bmi‐1* full‐length overexpression plasmid carrying a His‐tag in the pcDNA3.1 plasmid.

GATA4 is composed of an amino‐terminal GATA‐type transcription activator domain and a conserved carboxy‐terminal zinc finger DNA‐binding domain. We generated two truncated fragments and a full‐length *GATA4* carrying a Flag‐tag in the PEGFP‐C1 vector.

RING1B was divided into an amino‐terminal conserved protein containing a RING Zn‐finger, a carboxy‐terminal RAWUL domain, two truncated fragments and a full‐length *RING1B*. All had an HA‐tag in the PEGFP‐C1 vector.

*Ubiquitin* full‐length overexpression plasmid was generated carrying a Myc‐tag in the pcDNA3.1 vector.

### 2.17 Protein immunoprecipitation

Total proteins were used for immunoprecipitation using Pierce™ Crosslink Magnetic IP/Co‐IP kits (#88805, Thermo Scientific Pierce™ Crosslink Magnetic IP/Co‐IP Kit, Thermo Fisher Scientific, IL, USA) as recommended by the supplier. Primary antibodies against Bmi‐1 (#5856, Cell Signaling Technology, USA), RING1B (#5694, Cell Signaling Technology, USA), GATA4 (#19530, Proteintech, USA), p62 (#39749, Cell Signaling Technology, USA), anti‐ubiquitin (#91112, Cell Signaling Technology, USA), HSC70 (10654‐1‐AP, Proteintech, USA), DYKDDDDK Tag (Anti‐FLAG M2 antibody, #14793, Cell Signaling Technology, USA), His‐Tag (#12698, Cell Signaling Technology, USA), HA‐Tag (#3724, Cell Signaling Technology, USA) and Myc‐Tag (M1405‐5, Hangzhou HuaAn Biotechnology, China) were used.

### 2.18 Protein stability

*Bmi‐1* overexpressed AC16 cells or controlled AC16 cells or were planted into 10‐cm plates and treated with Ang II (1 × 10^–5^ for 70 h) to induce hypertrophy. Cells were cocultured with 5 μM^16^ MG132 (#474787, Sigma‐Aldrich, USA) or 100 nM^28^ bafilomycin A1 (Baf‐A1, #S1413, Sigma‐Aldrich, USA) for 12 h, then treated with 100 μM^29^ cycloheximide (CHX, #5.08739, Sigma‐Aldrich, USA) plus 5 μM MG132, or CHX plus 100 nM Baf‐A1 for indicated times. Then proteins were extracted to detect the target proteins with Western blots.

### 2.19 Ubiquitination assay

As previously described method,^29^ the 293T cells were transfected with plasmids including Myc‐ubiquitin, Flag‐GATA4, HA‐RING1B and/or His‐Bmi‐1, and lysed in the presence of deubiquitination inhibitor N‐ethylmaleimide (E3876, Sigma‐Aldrich, USA), and subjected to immunoprecipitation with anti‐DYKDDDDK Tag (Anti‐FLAG M2 antibody, #14793, Cell Signaling Technology, USA) antibody. Western blot was used to detect the ubiquitination levels with anti‐ubiquitin antibody (#91112, Cell Signaling Technology, USA).

### 2.20 Adeno‐associated virus carrying *Bmi‐1‐RING1B*

Serotype 9 adeno‐associated virus (AAV9) carrying the target sequence that overexpresses *Bmi‐1‐RING1B* or the corresponding control virus was designed and produced (Hanbio Biotechnology, China). Previous reports considered AAV9‐*cytomegalovirus (CMV)* viral packaging as an efficient and safe tool for cardiac gene transfer due to its consistent transduction efficiency and established cardiac tropism.^30^, ^31^, ^32^ It has been used in multiple preclinical studies, for example, in the first‐in‐human cardiac gene therapy trial because of its robust expression.^33^, ^34^ Because of the large base number of *Bmi‐1* (975 bp)–*RING1B* (1, 011 bp) complex, we used the universal promoter *CMV* promoter to better express *Bmi‐1–RING1B* complex. Eight‐week‐old male mice were injected with 100 μl AAV9‐*CMV*‐*Bmi‐1‐RING1B* (1 × 10^12^ v.g/ml) by caudal vein. After 2 weeks of AAV9‐*CMV*‐*Bmi‐1‐RING1B* treatment, these mice were anesthetised with 0.02 g/ml pentobarbital sodium (50 mg/kg body weight, intraperitoneal injection). Subcutaneous infused with angiotensin II (Ang II, #A9525, Sigma‐Aldrich, USA) for 4 weeks at a dose of 1.3 mg/kg/day was to induce cardiac hypertrophy using ALZET® Micro‐Osmotic Pumps (Model 1004) (#10370‐16, DURECT Corporation, USA).

### 2.21 Statistical analysis

As previously described, all analyses used GraphPad Prism software (Version 6.07; GraphPad Software Inc., San Diego, CA, USA).^35^ *p* Values were two‐sided and values less than .05 were considered statistically significant.^11^, ^15^, ^16^

## 3 RESULTS

### 3.1 Bmi‐1‐RING1B and autophagy are negatively related to SA‐PCH

**FIGURE 1:**
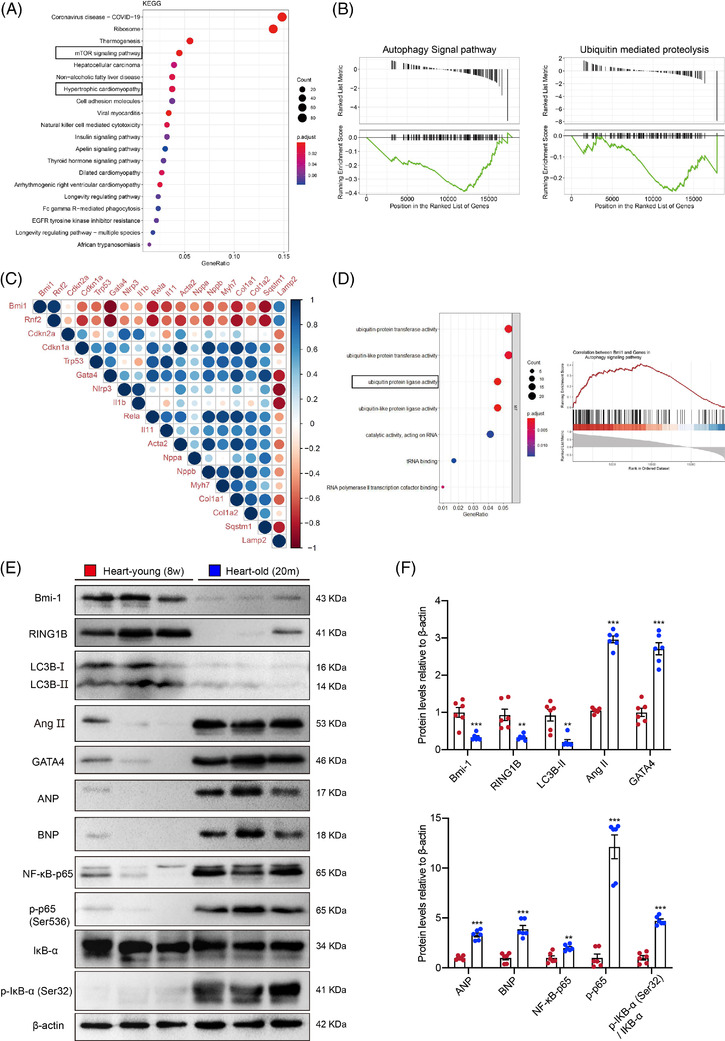
Bmi‐1‐RING1B and autophagy are negatively related to SA‐PCH. (A) Kyoto Encyclopedia of Genes and Genomes (KEGG) analysis was conducted using differentially expressed genes (DEGs) in young (14‐week‐old) and old (18‐month‐old) mouse hearts. (B) Gene set enrichment analysis (GSEA) on autophagy signal pathway and ubiquitin‐mediated proteolysis. (C) Genes Correlation analysis using 12‐ and 18‐ month mouse hearts RNAseq count data. (D) Gene Ontology (GO) analysis and GSEA were processed using genes that were highly related to *Bmi‐1*. (E) Western blots of cardiac extracts of WT young (8‐week‐old) or aging (20‐month‐old) mice showing Bmi‐1, RING1B, LC3BII, Ang II, GATA4, ANP, BNP, NF‐κB‐p65 and p‐p65 (Ser536), IκB‐α and p‐IκB‐α (Ser32); β‐actin was the loading control. (F) Protein levels relative to β‐actin were assessed by densitometric analysis. Six mice per group were used for experiments. Values are mean ± SEM of six determinations per group, **p < .01, ***p < .001 compared with the WT young mice, unpaired *t*‐test for bar graphs

To gain insight into the mechanism by which aging causes heart disease progression, bioinformatics methods were used to analyse the profile of gene expression in aging hearts. We searched the GEO database, and 14 weeks and 18 months of mouse heart RNA sequencing were analysed from the GSE161078 dataset. KEGG analysis using differentially expressed genes (DEGs) data and gene set enrichment analysis (GSEA) were performed. The results showed that the aging hearts had developed hypertrophic cardiomyopathy, and the autophagy‐related mTOR signal pathway was likely to be involved in the occurrence of the disease (Figure 1A). Then GSEA results showed that the autophagy levels and ubiquitin‐mediated protein degradation signal were downregulated in aging hearts (Figure 1B).

To clarify which gene plays a key role in the elderly hypertrophic cardiomyopathy, we conducted correlation analysis using 12 months and 18 months mouse heart RNA sequencing count data from the GSE161078 dataset and found that *Bmi‐1* and *Rnf2* (RING1B) were negatively related to the marker genes of aging, cardiac hypertrophy and inflammation (Figure 1C). Next, we performed Gene Ontology enrichment analysis and GSEA analysis on genes that are highly correlated to *Bmi‐1*, and the results showed that genes highly related to *Bmi‐1* were mostly involved in ubiquitin‐protein and ubiquitin‐like protein transferase activities and ubiquitin‐protein and ubiquitin‐like protein ligase activities, and the genes involved in the autophagy pathway were most positively correlated to *Bmi‐1* expression (Figure 1D).

To further verify the results of the bioinformatics analysis, protein levels in the heart tissues of young (8‐week‐old) and aging (20‐month‐old) mice were detected by Western blot, which showed that Bmi‐1, RING1B and LC3BII protein levels were downregulated; however, Ang II, GATA4, ANP, BNP, NF‐κB‐p65, p‐p65 (Ser536) and p‐IκB‐α (Ser32)/IκB‐α protein levels were upregulated in aged mice hearts compared with those from young mice (Figure 1E and F). The mRNA levels of *p65* (*RelA*) and *TRAF3IP2* were detected in myocardial tissues of young (8‐week‐old) and aging (20‐month‐old) mice. Results showed that in comparison with young mice, the mRNA levels of *p65* (*RelA*) and *TRAF3IP2* were increased in myocardial tissues of aged mice (Figure S1A).

### 3.2 *Bmi‐1* deficiency induces myocardial dysfunction, RAAS activation and senescence

To investigate how Bmi‐1 maintains myocardial function, 3‐, 6‐ and 9‐week‐old *Bmi‐1^–/–^* mice and WT littermates were studied with colour Doppler echocardiography. *Bmi‐1^–/–^* mice showed cardiac dysfunction as evidenced by decreased LVEF and LVFS compared with WT littermates at 3‐ and especially 9‐week‐old. At 6 weeks of age, the cardiac function of *Bmi‐1^–/–^* mice was closest to WT levels (Figure S2A–C).

We sought to determine whether the cardiac dysfunction caused by *Bmi‐1* deficiency is accompanied by activation of RAAS and cardiomyocyte senescence. Radioimmunoassay, Western blots and real‐time RT‐PCR were used. Ang II and ALD levels in plasma and renin and Ang II protein level in the aorta and myocardial tissues showed a significant increase in *Bmi‐1^–/–^* mice compared with WT littermates (Figure S2D–F). To investigate whether RAAS activation triggered by oxidative stress and DNA damage response (DDR) in heart was ameliorated by antioxidant N‐acetylcysteine (NAC) supplementation in *Bmi‐1^–/–^* mice, we observed these factors and cell senescence marker p16, p19, p21, p53, NF‐κB‐p65 and p‐p65, ROS levels, antioxidase superoxide dismutase 2 (SOD2) and DDR‐core molecule p‐Chk2 (Thr68) in the heart and aorta. Levels of ROS, p16, p19, p21, p53, NF‐κB‐p65, p‐p65 and p‐Chk2 (Thr68) proteins and *p16* mRNA significantly increased in the aorta and myocardial tissues from *Bmi‐1^–/–^* mice compared with WT littermates; however, SOD2 protein level decreased (Figure S2G–M). By electron microscopy, the number of mitochondria decreased. Mitochondria swelled and cristae were blurred. Myofibrils were reduced and disordered and lipid droplets and vacuoles appeared in the cytoplasm. NAC supplementation decreased ROS levels, ameliorated this ultrastructure, inhibited expression of renin, Ang II and p16 protein and improved cardiac function and cardiac hypertrophy in hearts from *Bmi‐1^–/–^* mice (Figure S3A–I).

### 3.3 *Bmi‐1* deficiency aggravates cardiac hypertrophy and decreases autophagic flux

**FIGURE 2:**
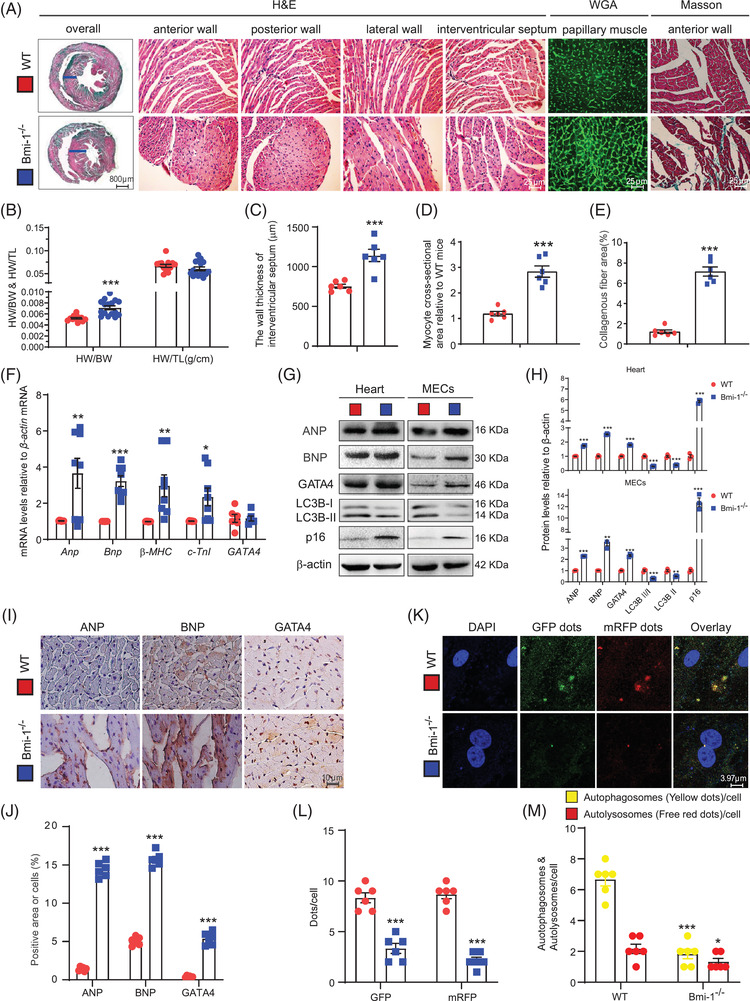
*Bmi‐1* deficiency aggravates cardiac hypertrophy and decreases autophagic flux. (A) Representative micrographs of paraffin‐embedded heart ventricular wall sections of 6‐week‐old *Bmi‐1^–/–^* mice and WT mice stained for haematoxylin‐eosin (H&E), wheat germ agglutinin (WGA) and Masson's trichrome (Masson) staining. (B) Heart weight relative to body weight (HW/BW) and heart weight relative to tibia length (HW/TL). (C) The wall thickness of interventricular septum. (D) Myocyte cross‐sectional area from *Bmi‐1^–/–^* mice relative to WT mice. (E) Collagenous fibre area from *Bmi‐1^–/–^* mice relative to WT mice was counted with anterior wall staining for Masson. (F) *Anp*, *Bnp*, *β‐MHC*, *c‐TnI* and *GATA4* mRNA levels in hearts by real‐time RT‐PCR, calculated as ratio to *β‐actin* mRNA and expressed relative to WT. (G) Western blots of cardiac tissue extracts and mouse embryonic cardiomyocytes (MECs) showing ANP, BNP, GATA4, LC3B and p16; β‐actin was the loading control. (H) Protein levels relative to β‐actin were assessed by densitometric analysis. (I) Representative micrographs of paraffin‐embedded heart sections immunohistochemical staining for ANP, BNP and GATA4. (J) The percentage of cells positive for ANP, BNP, GATA4 or positive area relative to total cells or area. (K) Representative micrographs of fluorescence with autophagy lentivirus transfection, with DAPI for nuclei. (L) Percentage of positive GFP dots or mRFP dots relative to total cells. (M) The number of autophagosomes and autolysosomes per cell. Six mice per group were used for experiments. Cell experiments were performed with three biological repetitions per group. Values are mean ± SEM from six determinations per group, *p < .05, **p < .01, ***p < .001 compared with WT group, unpaired Student's *t*‐test

To clarify whether *Bmi‐1* deletion led to cardiac hypertrophy, we observed cross‐sectional areas of cardiomyocytes from *Bmi‐1^–/–^* mice. Cardiac hypertrophy and fibrosis were analysed by H&E, WGA and Masson staining. Ventricular wall thickening, cardiac hypertrophy and interstitial fibrosis of myocardial tissues increased in *Bmi‐1^–/–^* mice compared with WT mice (Figure 2A and C–E). Heart‐to‐body‐weight (HW/BW) ratio also increased accordantly; however, no significant difference in heart weight to tibia length (HW/TL) ratio was observed (Figure 2B).

Using real‐time RT‐PCR, Western blots and immunohistochemical staining, hypertrophic pathological phenotypes were compared for *Bmi‐1^–/–^* and WT mice. Hypertrophic phenotypes were accompanied by the upregulation of hypertrophic genes and proteins ANP, BNP, *β‐MHC* and *c‐TnI*, and senescence biomarker p16 in myocardial tissues and cardiomyocytes (Figure 2F–H). However, mRNA for myocardial transcription factor *GATA4* was not changed in *Bmi‐1* ^–/–^ mice. Protein levels of GATA4 significantly increased in *Bmi‐1* ^–/–^ mice compared with WT mice (Figure 2F–J).

To determine whether cardiac hypertrophy in *Bmi‐1* ^–/–^ mice was associated with autophagy, mouse embryonic cardiomyocytes (MECs) were isolated and cultured from 13.5‐day foetal hearts from WT and *Bmi‐1^–/–^* mice and were infected with an autophagic flux‐detection lentivirus. Autolysosomes and autophagosomes significantly decreased in *Bmi‐1*‐null‐ MECs compared with WT MECs, just the same as the LC3B‐II/LC3B‐I and LC3B‐II levels (Figure 2G–H, K–M). These results showed that in *Bmi‐1^–/–^* mice, autophagy in myocardial tissue or MECs was significantly inhibited compared with WT mice.

### 3.4 *Bmi‐1* deficiency aggravates Ang II‐induced PCH by decreasing autophagic flux

**FIGURE 3:**
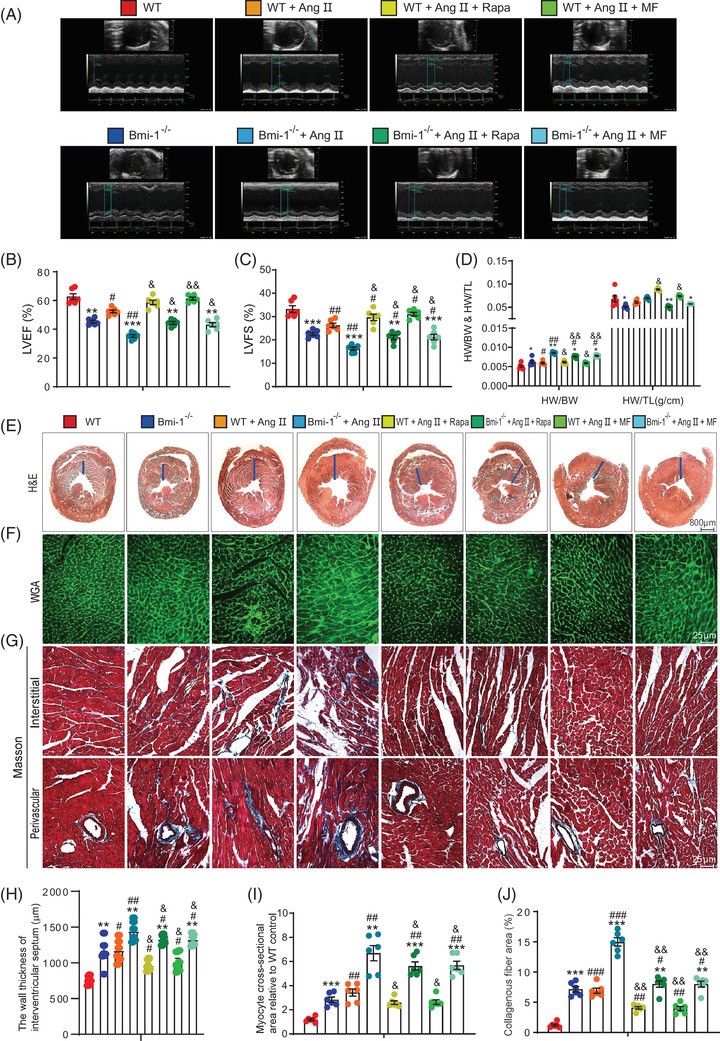
Autophagy agonist supplementation improves Ang II‐induced PCH in WT mice more than *Bmi‐1* ^–/–^ mice. Autophagy agonist metformin (as a water supplement, 1 mg/ml) or rapamycin intraperitoneal injection, 2 mg/kg/day), was administered to 4‐week‐old WT and *Bmi‐1^–/–^* mice that were treated with Ang II (1.3 mg/kg/day) for 4 weeks. (A) Colour Doppler echocardiography for 8‐week‐old mice. (B) Left ventricular ejection fraction (LVEF). (C) Left ventricular shortened fraction (LVFS). (D) The heart weight‐to‐body weight (HW/BW) and heart weight‐to‐tibia length (HW/TL) ratios were calculated. Representative micrographs of paraffin‐embedded heart ventricular wall sections for (E–G) haematoxylin‐eosin (H&E), wheat germ agglutinin (WGA) and Masson's trichrome (Masson) staining. (H) The wall thickness of interventricular septum. (I) Myocyte cross‐sectional area is relative to WT mice. (J) Collagenous fibre area. Six mice per group were used for experiments. Statistical analysis was performed with one‐way ANOVA for pairwise comparisons; values are mean ± SEM from six determinations per group, *p < .05, **p < .01, ***p < .001 compared to WT group with the same treatment; ^#^ *p* < .05, ^##^ *p* < .01, ^###^ *p* < .001 compared to control group with the same genotype; ^&^ *p* < .05, ^&&^ *p* < .01 compared to Ang II‐treated group with the same genotype

To further clarify the effect of Bmi‐1 on preventing the progress of Ang II‐induced PCH, *Bmi‐1^–/–^* mice and WT littermates were subcutaneously infused with Ang II to stimulate the development of PCH. After Ang II treatment, *Bmi‐1^–/–^* mice showed more serious cardiac hypertrophy than WT littermates as evidenced by decreased LVEF and LVFS analysed by colour Doppler echocardiography, increased HW/BW ratios and aggravated hypertrophic features and myocardial fibrosis by H&E, WGA and Masson's staining (Figure 3).

To determine whether aggravated SA‐PCH in *Bmi‐1*‐deficient mice is associated with autophagy downregulation, autophagy agonists metformin or rapamycin were administered to WT and *Bmi‐1^–/–^* mice treated with Ang II. Metformin or rapamycin supplementation increased LVEF and LVFS, decreased HW/BW ratios and ameliorated hypertrophic features and myocardial fibrosis in WT and *Bmi‐1^–/–^* mice with Ang II treatment (Figure 3). Rescue effects of metformin or rapamycin in *Bmi‐1^–/–^* mice were not good as those in WT mice. These results suggested that Bmi‐1 might prevent Ang II‐induced PCH by promoting autophagic flux.

### 3.5 *Bmi‐1* deficiency promotes GATA4‐dependent SA‐PCH by inhibiting autophagic degradation of GATA4

We further investigated whether Bmi‐1 prevented Ang II‐induced PCH by promoting autophagic degradation of GATA4 and inhibiting GATA4‐dependent myocardial hypertrophy, aging and inflammation. Metformin or rapamycin was administered to WT and *Bmi‐1^–/–^* mice treated with Ang II; GATA4, downstream signals and changes in autophagic flux were observed. With Ang II treatment, *Bmi‐1^–/–^* mice showed more serious myocardial hypertrophy, aging and inflammation than WT littermates. The evidence were as follows: increased protein expression with ANP, BNP and GATA4 (Figure S4A, B and E); upregulated mRNA for hypertrophic genes *Anp*, *Bnp*, *β‐MHC* and profibrotic genes α‐sarcomeric actin 1 (*Acta1*), *Acta2* and regulator of calcineurin 1.4 (*Rcan1.4*), and senescence gene *p16* (Figure S4C and D); increased p62, p16 and NF‐κB‐p65 protein levels and slightly increased LC3B‐II/LC3B‐I and LC3B‐II (Figure S4E and F). We checked autophagic vacuoles using electron microscope (Figure S4G) and observed LAMP2^+^p62^+^GATA4^+^ or LAMP2^+^LC3B^+^GATA4^+^ dots in cardiomyocytes (Figure S5). After supplementing with metformin or rapamycin, myocardial hypertrophy, aging and inflammation‐related genes and proteins of Ang II‐treated WT and *Bmi‐1^–/–^* mice were partially rescued. Rescued effects of metformin or rapamycin were lower in *Bmi‐1^–/–^* mice than in WT mice (Figures S4 and S5).

**FIGURE 4:**
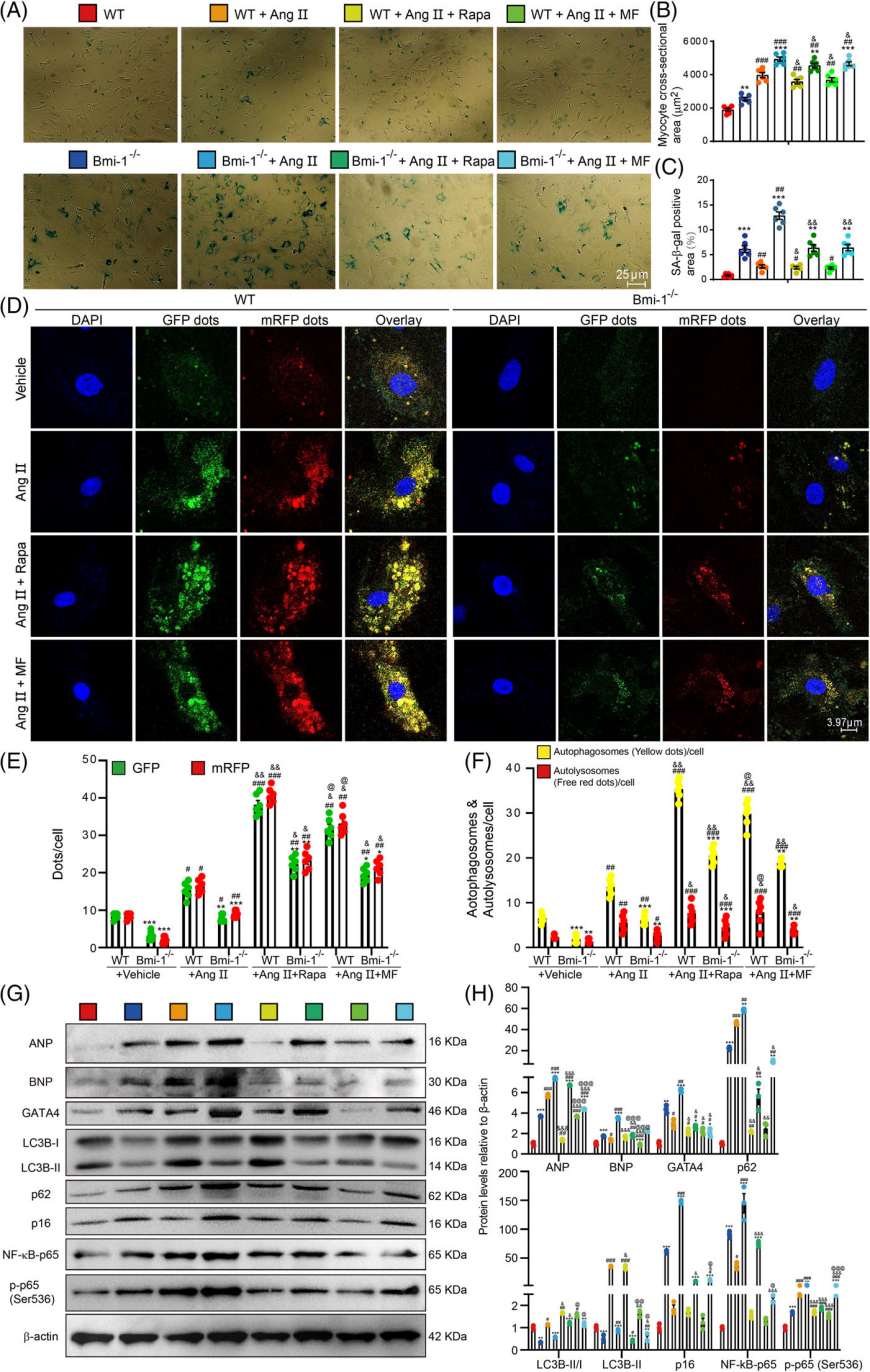
Autophagy agonist supplementation improves GATA4‐dependent SA‐PCH of embryonic cardiomyocytes in WT mice more than *Bmi‐1* ^–/–^ mice. Mouse embryonic cardiomyocytes (MECs) were isolated and cultured from 13.5‐day foetal hearts, and were treated with Ang II (5 × 10^–6^ mol/L for 70 h) to induce hypertrophy and were rescued with metformin (MF) (2.5 mM for 70 h) or rapamycin (Rapa) (100 nM for 70 h). (A) Representative micrographs of MECs stained for senescence‐associated β‐galactosidase (SA‐β‐gal). (B) Myocyte cross‐sectional area according to graph A. (C) The percentage of SA‐β‐gal‐positive area of myocytes. (D) Representative micrographs of WT and *Bmi‐1^–/–^* MECs, which transfected with autophagy lentivirus for 24 h under the treatment with Ang II together with metformin or rapamycin. (E) Percentage of positive GFP dots or mRFP dots relative to total cells in different treatment groups. (F) The number of autophagosomes and autolysosomes per cell. (G) Western blots of extracts from MECs showing ANP, BNP, GATA4, LC3B, p62, p16, NF‐κB‐p65 and p‐p65 (Ser536); β‐actin was the loading control. (H) Protein levels relative to β‐actin were assessed by densitometric analysis. The experiments were performed with three biological repetitions per group. Statistical analysis was performed with one‐way ANOVA test; values are mean ± SEM from six determinations per group, *p < .05, **p < .01, ***p < .001 compared to WT MECs with the same treatment; ^#^ *p* < .05, ^##^ *p* < .01, ^###^ *p* < .001 compared to control group with the same genotype; ^&^ *p* < .05, ^&&^ *p* < .01, ^&&&^ *p* < .001 compared to Ang II‐treated group with the same genotype; ^@^ *p* < .05, ^@@^ *p* < .01, ^@@@^ *p* < .001 compared to Ang II & Rapa‐treated group with the same genotype

To verify if *Bmi‐1* deficiency caused disorders in GATA4‐selective autophagy and GATA4‐dependent cell hypertrophy, senescence and inflammation in cardiomyocytes, MECs were treated with Ang II to induce hypertrophy with rescue with metformin or rapamycin. Senescence‐associated β‐galactosidase (SA‐β‐gal) staining was performed and results showed that *Bmi‐1* deficiency induced cardiomyocyte hypertrophy and senescence compared with WT MECs. Ang II treatment promoted cardiomyocyte hypertrophy and senescence in both *Bmi‐1* deficient and WT MECs and was ameliorated by supplementation with metformin or rapamycin. Rescue effects of metformin or rapamycin on preventing cardiomyocyte hypertrophy and senescence were lower in *Bmi‐1^–/–^* mice than WT mice (Figure 4A–C). Autophagic lentivirus was used to infect WT and *Bmi‐1^–/–^* MECs treated with Ang II and/or supplemented with rapamycin or metformin. After Ang II treatment, autolysosomes and autophagosomes per cell, and the LC3‐II/LC3‐I ratio and LC3B‐II protein were lower, and p62 protein was higher and protein levels of ANP, BNP, GATA4, p16, NF‐κB‐p65 and p‐p65 (Ser536) were higher in *Bmi‐1*‐null‐MECs than WT MECs. After supplementing with metformin or rapamycin, autophagic flux and GATA4‐dependent myocardial hypertrophy, aging and inflammation were partially rescued. Rescue effects of metformin or rapamycin were lower in *Bmi‐1^–/–^* mice than in WT mice (Figure 4D–H).

To determine the impact of mTOR signaling on p62‐mediated GATA4‐autophagic degradation, we used ULK1 inhibitor SBI‐0206965 and/or rapamycin to treat Ang II‐induced‐WT MECs and found SBI‐0206965 inhibited p62‐mediated autophagic degradation of GATA4 induced by inhibition of mTOR (Figure S6).

### 3.6 *Bmi‐1* overexpression represses GATA4‐dependent SA‐PCH by promoting GATA4 autophagic degradation

**FIGURE 5:**
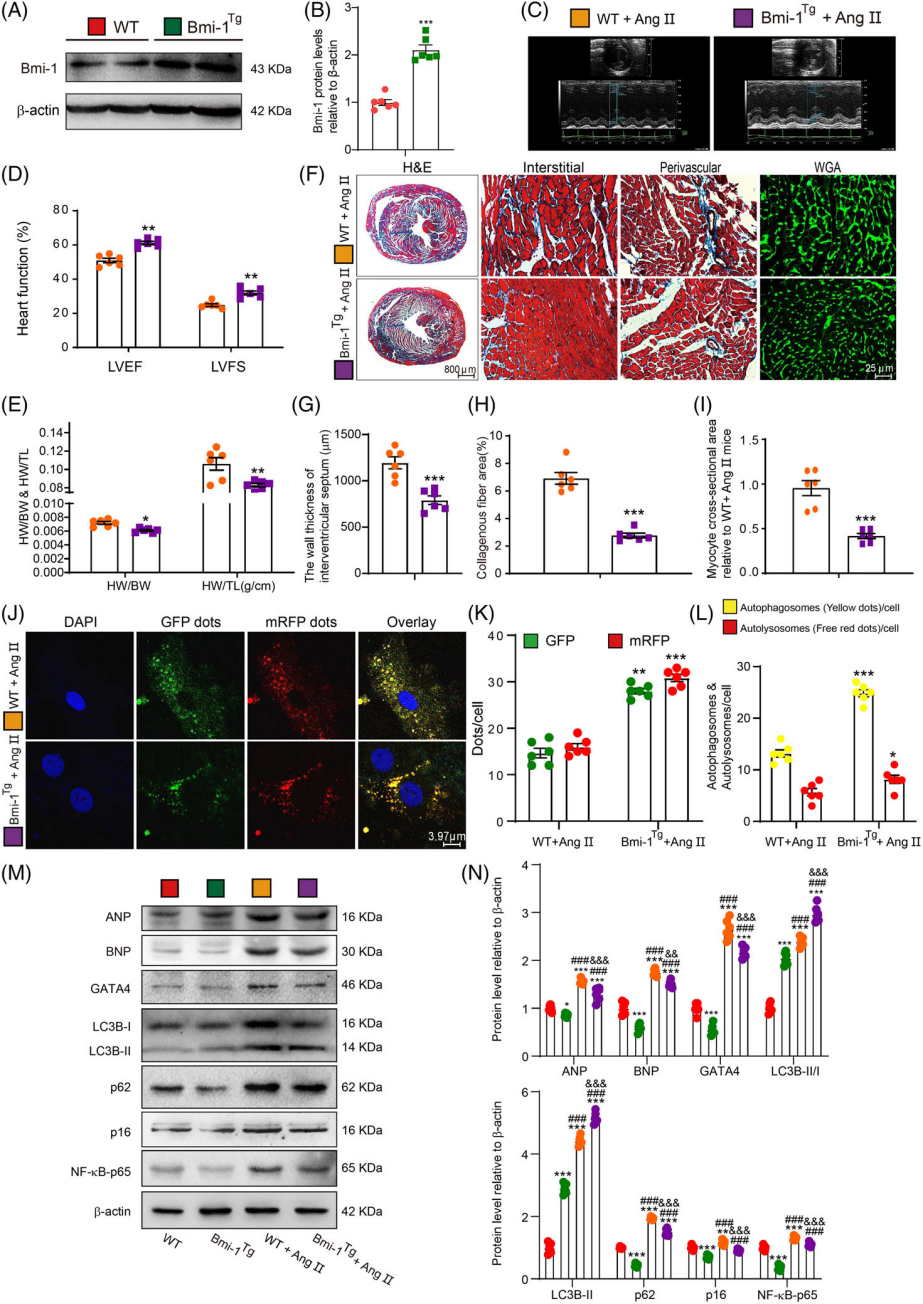
*Bmi‐1* overexpression in myocardial tissue represses GATA4‐dependent SA‐PCH by promoting GATA4 autophagic degradation. (A) Western blots for cardiac tissue extracts from 7‐week‐old WT and *Bmi‐1^Tg^* mice showing Bmi‐1 protein levels; β‐actin was the loading control. (B) Protein levels relative to β‐actin were assessed by densitometric analysis. (C–D) After treatment with Ang II (1.3 mg/kg/day) for 4 weeks, colour Doppler echocardiography was conducted for 8‐week‐old WT and *Bmi‐1^Tg^* mice. (E) Heart weight relative to body weight (HW/BW) and heart weight relative to tibia length (HW/TL). (F) Haematoxylin‐eosin (H&E), wheat germ agglutinin (WGA) and Masson's trichrome (Masson) staining. (G) The wall thickness of interventricular septum. (H) Collagenous fibre area. (I) Myocyte cross‐sectional area was calculated relative to Ang II‐treated WT mice. (J) Representative confocal micrographs of WT and *Bmi‐1^Tg^* MECs after autophagy lentivirus transfection for 24 h, with DAPI for nuclei. (K) Percentage of GFP‐ or mRFP‐positive dots relative to total cells. (L) The number of autophagosomes and autolysosomes per cell. Six mice per group were used for experiments. Cell experiments were performed with three biological repetitions per group. Statistical analysis was performed with unpaired Student's *t*‐test; values are mean ± SEM from six determinations per group, *p < .05, **p < .01, ***p < .001 compared with WT group. (M) Western blots for cardiac tissue extracts showing ANP, BNP, GATA4, LC3B, p62, p16, and NF‐κB‐p65; β‐actin was the loading control. (N) Protein levels relative to β‐actin were assessed by densitometric analysis. Statistical analysis was performed with one‐way ANOVA test. Six mice per group were used for experiments. Values are mean ± SEM from six determinations per group, *p < .05, **p < .01, ***p < .001 compared to WT group; ^###^ *p* < .001 compared to *Bmi‐1^Tg^* group; ^&&^ *p* < .01, ^&&&^ *p* < .001 compared to Ang II‐treated WT group

We further evaluated if *Bmi‐1* overexpression in cardiomyocytes rescued Ang II‐induced PCH in vivo. We generated a line of *Bmi‐1*‐overexpressing transgenic (*Bmi‐1^Tg^*) mice that specifically expressed from the mesenchymal origin *prx‐1* promoter. This line of *Bmi‐1^Tg^* mice had increased *Bmi‐1* protein in heart tissues (Figures 5A and B and S7A and B) and developed normal cardiac function and structure (Figure S7C and D).

To induce cardiac hypertrophy*, Bmi‐1^Tg^* mice and WT littermates were infused with Ang II for 4 weeks. Significant increases of LVEF and LVFS and decreases in HW/BW and HW/TL ratios, wall thickness of the interventricular septum, cardiac fibrosis and cardiomyocyte hypertrophy were observed in *Bmi‐1^Tg^* mice compared with WT mice (Figure 5C and 5I).

After Ang II treatment, in heart of *Bmi‐1^Tg^* mice compared to WT mice, autolysosomes and autophagosomes per cell, and the ratio of LC3‐II/LC3‐I and LC3B‐II showed more increases, and p62, ANP, BNP, GATA4, p16 and NF‐κB‐p65 expressions showed more decreases (Figure 5J–N). After Ang II treatment, compared with WT hearts, *Bmi‐1^–/–^* hearts showed significant increases in NF‐κB‐p65‐dependent SASP including IL‐1β, IL‐6 and TNF‐α; however, *Bmi‐1^Tg^* hearts showed significant decreases in NF‐κB‐p65‐dependent SASP (Figure S8A–C).

### 3.7 Bmi‐1 in human cardiomyocytes significantly inhibits Ang II‐induced GATA4‐dependent SA‐PCH through promoting GATA4 autophagic degradation

**FIGURE 6:**
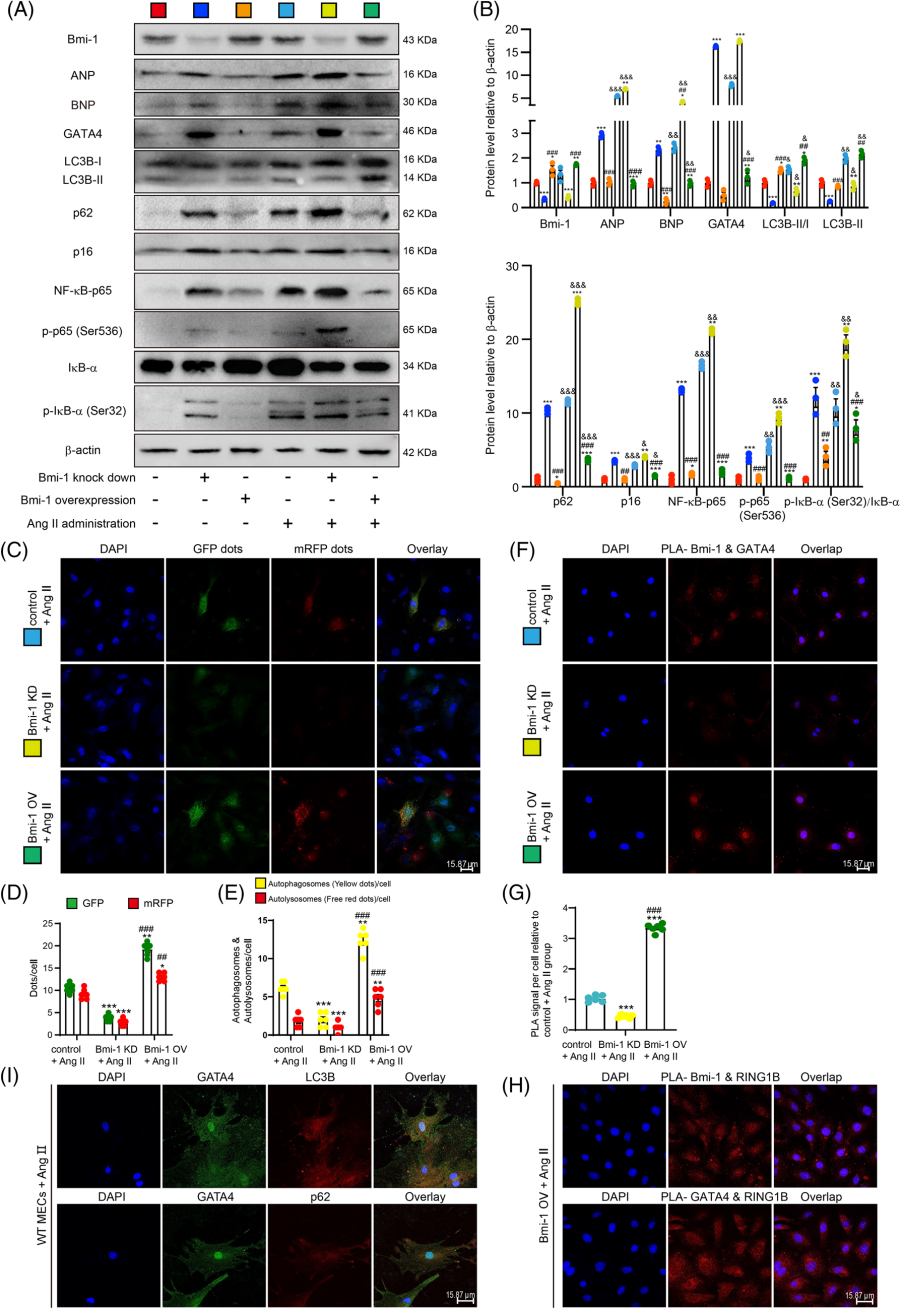
*Bmi‐1* in human cardiomyocytes significantly inhibits Ang II‐induced GATA4‐dependent SA‐PCH. The lentivirus with *Bmi‐1* overexpression or knockdown were constructed and transfected to human myocardial AC16 cells. AC16 cells were treated with Ang II (1 × 10^–5^ for 70 h) to induce hypertrophy. (A) Western blots of cell extracts showing Bmi‐1, ANP, BNP, GATA4, LC3B, p62, p16, NF‐κB‐p65, p‐p65 (Ser536), IκB‐α and p‐IκB‐α (Ser32) protein levels; β‐actin was the loading control. (B) Protein levels relative to β‐actin were assessed by densitometric analysis. The experiments were performed with three biological repetitions per group. Statistical analysis was performed with one‐way ANOVA test; values are mean ± SEM from three determinations per group, *p < .05, **p < .01, ***p < .001 compared with control group; ^##^ *p* < .01, ^###^ *p* < .001 compared to *Bmi‐1* knockdown group; ^&^ *p* < .05, ^&&^ *p* < .01, ^&&&^ *p* < .001 compared with the control group transfected with the same lentivirus. (C) Representative confocal micrographs of Ang II‐treated AC16 cells after transfecting with lentivirus of *Bmi‐1* overexpression or knockdown and then with autophagy lentivirus, with DAPI for nuclei. (D) Percentage of GFP‐ or mRFP‐positive dots relative to total cells. (E) The number of autophagosomes and autolysosomes per cell. The experiments were performed with three biological repetitions per group. AC16 cells treated with Ang II, (F) representative micrographs of Duolink Proximity Ligation Assay (PLA) for interaction between Bmi‐1 and GATA4, with DAPI for nuclei. (G) PLA signal per cell relative to the Ang II‐treated control group. Statistical analysis was performed with one‐way ANOVA test. Cell experiments were performed with three biological repetitions per group. Values are mean ± SEM from six determinations per group, *p < .05, **p < .01, ***p < .001 compared with Ang II‐treated control group; ^##^ *p* < .01, ^###^ *p* < .001 compared to Ang II‐treated *Bmi‐1* knockdown group. (H) Representative micrographs of PLA for interaction between Bmi‐1 and RING1B, or GATA4 and RING1B, with DAPI for nuclei. (I) Mouse embryonic cardiomyocytes (MECs) were isolated and cultured from 13.5‐day WT foetal hearts, and were treated with Ang II (5 × 10^–6^ mol/L for 70 h) to induce hypertrophy. Representative micrographs immunofluorescently labelled for LC3B or p62 and GATA4 in MECs, with DAPI for nuclei

To study the effect of *Bmi‐1* knockdown or overexpression on Ang II‐induced hypertrophy of human cardiomyocytes, a lentivirus for *Bmi‐1* knockdown or overexpression was constructed and transfected into human myocardial AC16 cells. The expression of Bmi‐1 was significantly decreased after *Bmi‐1* knockdown and increased after *Bmi‐1* overexpression. Compared to vehicle group, *Bmi‐1* knockdown downregulated the expression of LC3II/I ratio and LC3B‐II protein, autolysosomes and autophagosomes per cell, however, increased the expressions of p62, ANP, BNP, GATA4, p16, NF‐κB‐p65, p‐p65 (Ser536) and p‐IκB‐α (Ser32)/ IκB‐α; *Bmi‐1* overexpression increased the expression of LC3II/I ratio and LC3B‐II protein, autolysosomes and autophagosomes per cell, however, decreased the expressions of p62, ANP, BNP, GATA4, p16 and NF‐κB‐p65, p‐p65 (Ser536) and p‐IκB‐α (Ser32)/IκB‐α (Figure 6A–E). Thus, Bmi‐1 could alleviate Ang II‐induced GATA4‐dependent SA‐PCH; however, the effect of *Bmi‐1* overexpression was inhibited by autophagy inhibitor Bafilomycin A1(Baf‐A1) (Figure S9A and B).

To further confirm whether Bmi‐1 promotes GATA4 degradation by proteasome and/or autophagy, AC16 cells were stably infected with *Bmi‐1* overexpression lentivirus and pretreated with Ang II. GATA4 protein levels were assessed in control (CN) and *Bmi‐1* overexpressed (Bmi‐1 OV) groups cultured for 0, 1, 2, and 4 h in the presence of cycloheximide (CHX) to inhibit protein synthesis. Compared to CN group, GATA4 protein levels were dramatically decreased in *Bmi‐1* OV group, suggesting that *Bmi‐1* overexpression promoted GATA4 degradation (Figure S9C–H). Compared to Ang II‐ and CHX‐treated group, the addition of the autophagy inhibitor Baf‐A1 impeded GATA4 degradation more obviously than the addition of the proteasome inhibitor MG132 (Figure S9I). Thus, the degradation of GATA4 by Bmi‐1 mainly depended on autophagy rather than proteasome.

### 3.8 GATA4 interacted with Bmi‐1‐RING1B and autophagically degraded in the cytoplasm

In order to clarify the interaction between Bmi‐1‐RING1B and GATA4 and the subcellular localisation of GATA4 autophagy degradation, Duolink PLA in situ was used to confirm the interaction of Bmi‐1 and GATA4, Bmi‐1 and RING1B, and GATA4 and RING1B in Ang II‐induced AC16 cells. GATA4 and Bmi‐1 or Bmi‐1 and RING1B combined mainly in the nucleus; however, the combination of GATA4 and RING1B increased in the cytoplasm. *Bmi‐1* overexpression promoted the combination of Bmi‐1 and GATA4, and *Bmi‐1* knockdown reduced it (Figure 6F–H). To observe the subcellular localisation of GATA4 autophagic degradation, GATA4 was colocalised with LC3B or p62, showing the combination of GATA4 and LC3B or p62 in the cytoplasm, suggesting that GATA4 autophagic degradation happened in the cytoplasm (Figure 6I).

To determine whether p62‐mediated lysosomal degradation of GATA4 is chaperon‐mediated autophagy (CMA), the amino acid sequences of GATA4 protein and motif ‘KFERQ’ from mouse or human were aligned using online tools (Uniprot/Align: https://www.uniprot.org/align/) and showed negative results (Supplementary Information 8). We also used Duolink PLA in situ and protein Co‐immunoprecipitation (Co‐IP) to confirm the interaction between HSC70 and GATA4 and found no combination between them (Figure S10A–C). Thus, p62‐mediated lysosomal degradation of GATA4 was not HSC70‐mediated CMA.

### 3.9 GATA4 binds Bmi‐1‐RING1B and is ubiquitinated, recognised by p62 and degraded by selective autophagy

**FIGURE 7:**
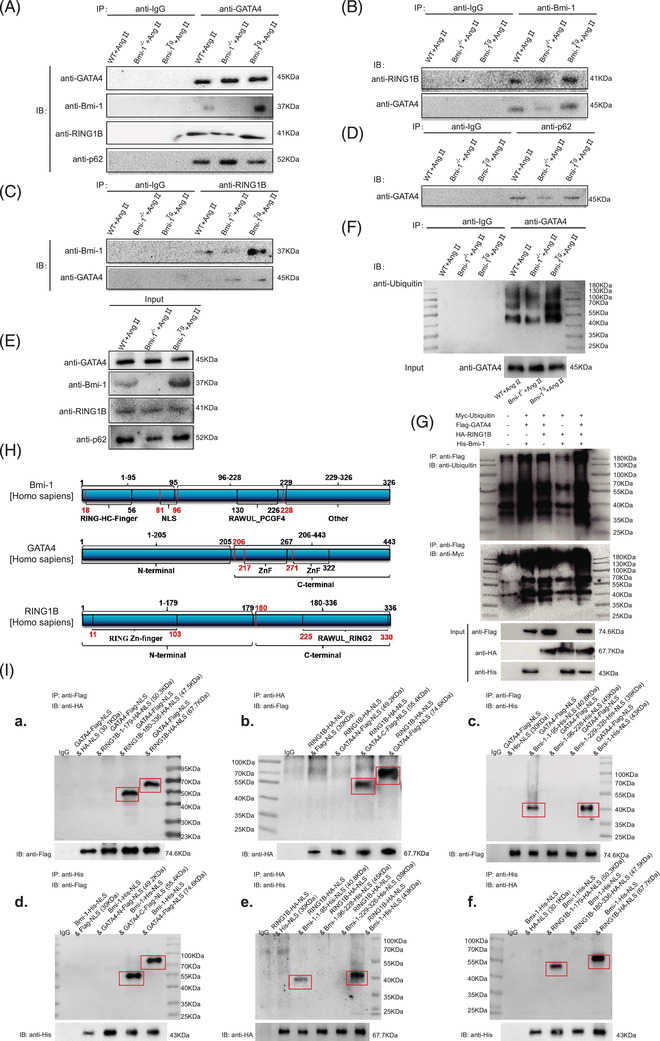
GATA4 binds Bmi‐1‐RING1B and is ubiquitinated, recognised by p62 and degraded by selective autophagy. (A) Myocardial tissue proteins from Ang II‐treated WT, *Bmi‐1^–/–^* and *Bmi‐1^Tg^* mice were extracted for anti‐GATA4 immunoprecipitation. Western blots were used for detecting GATA4, Bmi‐1, RING1B and p62. (B) The above myocardial tissue proteins were extracted for anti‐Bmi‐1 immunoprecipitation. Western blots were used for detecting RING1B and GATA4. (C) The above myocardial tissue proteins were extracted for anti‐RING1B immunoprecipitation. Western blots were used for detecting Bmi‐1 and GATA4. (D) The above myocardial tissue proteins were extracted for anti‐p62 immunoprecipitation. Western blots were used for detecting GATA4. (E) The above myocardial tissue proteins were extracted for input experiments. Western blots were used for detecting GATA4, Bmi‐1, RING1B and p62. (F) Mouse embryonic cardiomyocytes (MECs) from WT, *Bmi‐1^–/–^* and *Bmi‐1^Tg^* mice were isolated and cultured from 13.5‐day foetal hearts, and treated with Ang II (5 × 10^–6^ mol/L for 70 h) to induce hypertrophy, and were extracted for anti‐GATA4 immunoprecipitation. Western blots were used for detecting ubiquitin in immunoprecipitation samples and for detecting GATA4 in input samples. (G) The 293T cells were transfected with full‐length plasmids including Myc‐ubiquitin, Flag‐GATA4, HA‐RING1B and/or His‐Bmi‐1. Cell proteins were extracted for anti‐Flag‐tag immunoprecipitation and detected anti‐ubiquitin and ‐Myc‐tag with Western blots. The input proteins were detected anti‐Flag‐tag, ‐HA‐tag or ‐His‐tag with Western blots. (H) Plasmids of truncated fragments and full length from Bmi‐1 (labelled with His tag), RING1B (labelled with HA tag) and GATA4 (labelled with Flag tag) were co‐transfected in 293T cells. The above cells were extracted for anti‐Flag‐tag, ‐HA‐tag or ‐His‐tag immunoprecipitation. (I) Western blots were used for detecting anti‐HA‐tag, ‐Flag‐tag or ‐His‐tag in immunoprecipitation and input samples

To determine the molecular mechanism by which Bmi‐1 promoted autophagic degradation of GATA4, myocardial tissue proteins were extracted from WT, *Bmi‐1^–/–^* and *Bmi‐1^Tg^* mice subcutaneously infused with Ang II. Protein immunoprecipitation was performed, and GATA4, Bmi‐1, RING1B, and p62 antibodies were used to capture proteins that bound bait proteins. Antibody against ubiquitin was used to detect the ubiquitination levels of GATA4. Results showed that GATA4 bound to RING1B, Bmi‐1 and p62. Bmi‐1 bound to RING1B (Figure 7A–E). GATA4 ubiquitination levels in Ang II‐treated *Bmi‐1^–/–^* MECs were significantly lower than in Ang II‐treated WT and *Bmi‐1^Tg^* MECs (Figure 7F). With ubiquitination assay, Bmi‐1 and RING1B co‐overexpression obviously increased the level of ubiquitination for GATA4 (Figure 7G). These results suggested that the formation of PRC1 protein complex by Bmi‐1‐RING1B promoted the ubiquitination of GATA4, ubiquitinated GATA4 was recognised by p62, translocated to autophagosomes to form autophagolysosomes and degraded.

Analysis of protein sequence alignment showed that a similar domain was labelled as a zinc finger in GATA4 (residues 214–320), GATA3 (residues 261–367) and GATA6 (residues 382–488) in mice. A similar domain was labelled as a zinc finger in GATA4 (residues 215–321), GATA3 (residues 261–367) and GATA6 (residues 388–494) in humans (Supplementary Information 6 and 7). To determine the combined domains of Bmi‐1, RING1B and GATA4, plasmid construction and transfection of truncated fragments were conducted. We found that Bmi‐1 combined with RING1B (residues 1–179) and C‐terminus of GATA4 (residues 206–443 including zinc finger domains) through residues 1–95 including a RING‐HC‐finger. RING1B combined with C‐terminus of GATA4 (residues 206–443 including zinc finger domains) through the C‐terminus (residues 180–336) (Figure 7H and I).

To compare the effect of residues 1–95 and full length (FL) of Bmi‐1 in ubiquitination of GATA4, the overexpression plasmids of ubiquitin, GATA4, RING1B and Bmi‐1‐FL or Bmi‐1‐1‐95 residues were transfected into 293T cells. Results showed that Bmi‐1‐FL has a better ubiquitination modification effect on GATA4 than Bmi‐1‐1‐95 residues (Figure S11).

### 3.10 AAV9‐*CMV‐Bmi‐1‐RING1B* treatment significantly attenuates GATA4‐dependent SA‐PCH through promoting GATA4 autophagic degradation

**FIGURE 8:**
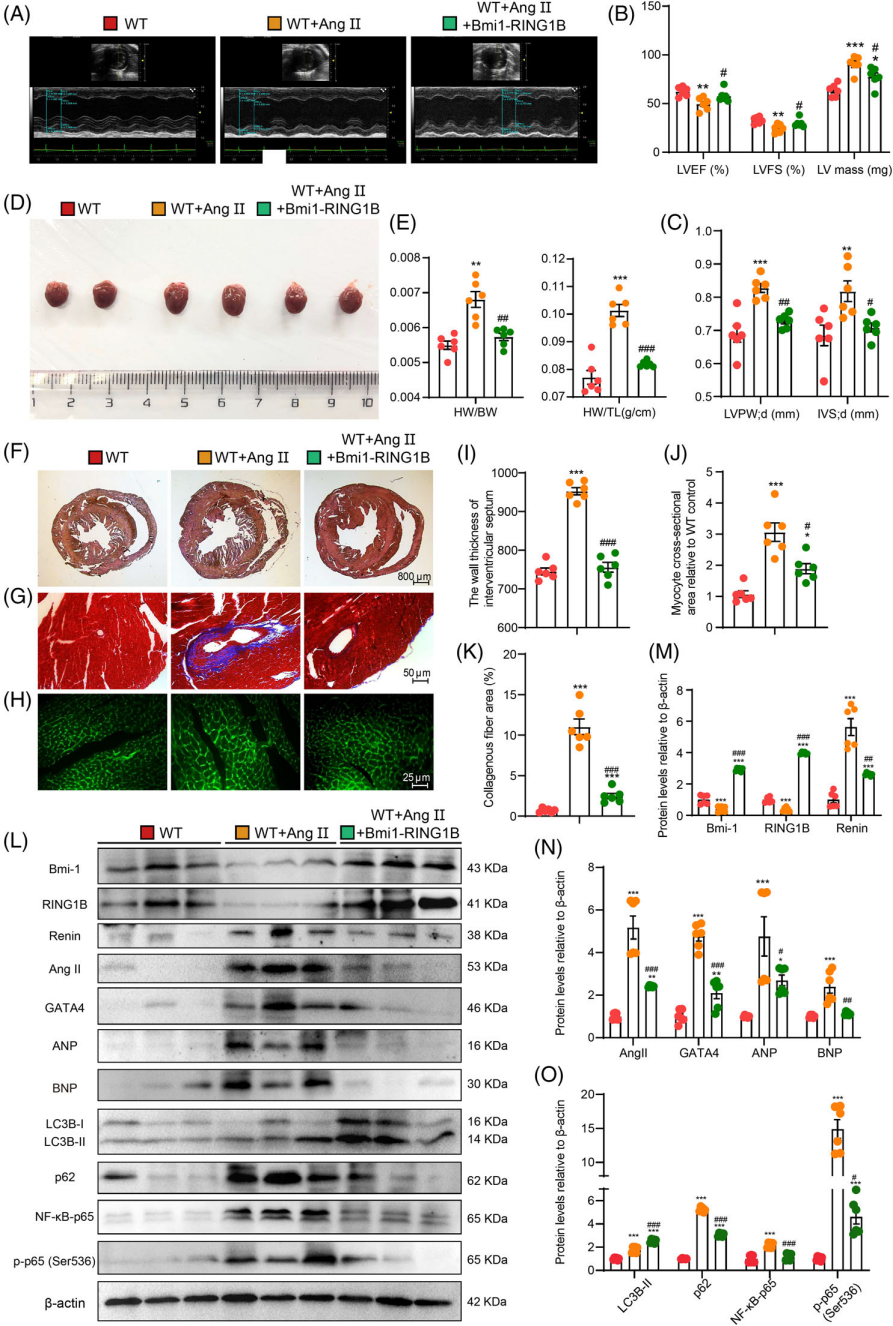
AAV9‐*CMV‐Bmi‐1‐RING1B* treatment significantly attenuates SA‐PCH. AAV9‐*CMV‐Bmi‐1‐RING1B* or the corresponding control virus was constructed and injected into 8‐week‐old mice for 2 weeks, then PCH was induced by Ang II (1.3 mg/kg/day) for 4 weeks. (A) Colour Doppler echocardiography for WT mice, AAV9‐*CMV‐Bmi‐1‐RING1B*‐ and Ang II‐treated WT (WT + Ang II + Bmi‐1‐RING1B) mice, and the corresponding control virus‐ and Ang II‐treated WT (WT + Ang II) mice. (B) Left ventricular ejection fraction (LVEF), left ventricular shortened fraction (LVFS) and left ventricular mass (LV mass). (C) Left ventricular posterior wall (LVPW) and interventricular septum (IVS). (D) General images of hearts for WT, WT + Ang II and WT + Ang II + Bmi‐1‐RING1B mice. (E) The heart weight‐to‐body weight (HW/BW) and heart weight‐to‐tibia length (HW/TL) ratio were calculated. (F–H) Haematoxylin‐eosin (H&E), Masson's trichrome (Masson) and wheat germ agglutinin (WGA) staining. (I) The wall thickness of interventricular septum. (J) Myocyte cross‐sectional area is relative to WT mice. (K) Collagenous fibre area. (L) Western blots for cardiac tissue extracts showing Bmi‐1, RING1B, Renin, Ang II, GATA4, ANP, BNP, LC3B, p62, p16, NF‐κB‐p65 and p‐p65 (Ser536); β‐actin was the loading control. (M–O) Protein levels relative to β‐actin were assessed by densitometric analysis. Statistical analysis was performed with one‐way ANOVA test. Values are mean ± SEM of six determinations per group, *p < .05, **p < .01, ***p < .001 compared with WT mice; ^#^ *p* < .05, ^##^ *p* < .01, ^###^ *p* < .001 compared to WT + Ang II mice

To investigate whether Bmi‐1‐RING1B co‐overexpression in heart could ameliorate Ang II‐induced SA‐PCH, mice were treated with Ang II after AAV9‐*CMV*‐*Bmi‐1‐RING1B* or the corresponding control virus pretreatment for 4 weeks. Compared with vehicle group, Ang II and the corresponding control virus treatment decreased LVEF and LVFS, however, increased left ventricular mass (LV), diastolic wall thickness of left ventricle (LVPW; d), diastolic ventricular septal thickness (IVS; d), the size of heart, HW/BW ratio, HW/TL ratio, collagenous fibre area and myocyte cross‐sectional area. Compared with Ang II‐treated group and the corresponding control virus‐treated group, Ang II‐ and AAV9‐*CMV*‐*Bmi‐1‐RING1B* treatment significantly attenuates the above disorder of heart function and structure induced by Ang II treatment (Figure 8A–K). We also found that in comparison with vehicle group, an obvious increase was observed in the protein level of renin, Ang II, GATA4, ANP, BNP, p62, NF‐κB‐p65 and p‐p65 (Ser536); however, a significant decrease was observed in the protein level of Bmi‐1, RING1B and LC3B‐II in Ang II‐treated group and the corresponding control virus‐treated group. Compared with Ang II‐treated group and the corresponding control virus‐treated group, an obvious decrease was observed in the protein level of renin, Ang II, GATA4, ANP, BNP, p62, NF‐κB‐p65 and p‐p65 (Ser536); however, a significant increase was observed in the protein level of Bmi‐1, RING1B and LC3B‐II in Ang II‐ and AAV9‐*CMV*‐*Bmi‐1‐RING1B*‐treated group (Figure 8L–O).

### 3.11 Bmi‐1‐RING1B prevented GATA4‐dependent SA‐PCH in human myocardium by promoting autophagic degradation

To determine if Bmi‐1‐RING1B prevented GATA4‐dependent SA‐PCH in the human myocardium by promoting autophagic degradation of GATA4, Bmi‐1, RING1B, p16, GATA4, ANP, p62 and LC3B protein levels in the human myocardium were detected by ELISA and were analysed for correlations. We found that p16 positively correlated with ANP, GATA4 and age, and negatively correlated with LC3B, Bmi‐1 and RING1B. GATA4 positively correlated with p62 and negatively correlated with Bmi‐1 and LC3B. Protein p62 positively correlated with ANP and negatively correlated with Bmi‐1 and LC3B. LC3B positively correlated with Bmi‐1 and RING1B and negatively correlated with ANP. Bmi‐1 positively correlated with RING1B and negatively correlated with ANP (Figure S12). These results indicated that accumulation of GATA4 and ANP in human senescent cardiomyocytes was caused by downregulated Bmi‐1‐RING1B‐dependent autophagic degradation.

To determine whether ANP‐, BNP‐, GATA4‐ and LC3B‐positive cells or areas in human myocardial tissues correlated with p16 protein levels, frozen tissue sections of human myocardial tissues were used. With increased p16 protein levels, ANP‐, BNP‐, GATA4‐positive cells or areas increased in the human myocardium while LC3B‐positive cells or areas decreased (Figure S13).

## 4 DISCUSSION

These data demonstrated that Bmi‐1 maintained cardiac function and prevented SA‐PCH by promoting selective autophagy for degrading GATA4. *Bmi‐1* deficiency promoted GATA4‐dependent SA‐PCH by increasing GATA4 protein and hypertrophy‐related molecules transcribed by GATA4 such as ANP and BNP. These changes led to cardiac dysfunction, cardiomyocyte hypertrophy, cell senescence and SASP. *Bmi‐1* overexpression repressed GATA4‐dependent SA‐PCH. Bmi‐1‐RING1B prevented GATA4‐dependent SA‐PCH in human cardiomyocytes by promoting autophagic degradation. GATA4 is ubiquitinated after combining with Bmi‐1‐RING1B, which is then recognised by p62, translocated to autophagosomes to form autophagolysosomes and degraded. Downregulated GATA4 ameliorated SA‐PCH and cardiac dysfunction. AAV9‐*CMV‐Bmi‐1‐RING1B* treatment could ameliorate GATA4‐dependent SA‐PCH.

Aging leads to progressive deterioration in cardiac structure and function.^3^ Cardiac aging is an important risk factor for hypertrophy, fibrosis, inflammation and contractile dysfunction.^3^, ^36^ Bmi‐1 is essential for maintaining mitochondrial function and preventing SIPS that is characterised by ROS accumulation, redox imbalance, DDR activation and cell senecence.^11^, ^12^, ^15^ Previous work from our group showed that *Bmi‐1* knockout causes dysfunction, premature senescence and associated proinflammation and profibrotic diseases in multiple organs.^11^, ^13^, ^16^, ^22^, ^23^ Ang II and ALD are elevated in plasma from *Bmi‐1* null mice compared to WT littermates.^11^ Mitochondrial dysfunction decreases ATP production and results in cardiac function decline and activation of RAAS.^37^ A previous study demonstrated that Bmi‐1 maintains mitochondrial function and ATP production in heart.^12^ Ang II, a key effector peptide of RAAS, is mainly triggered by excessive ROS and DDR, and it promotes oxidant production, destroys mitochondrial function and accelerates aging.^38^, ^39^ Several lines of evidence suggest that antioxidant administration inhibits RAAS activation through quenching ROS and preventing DDR.^11^, ^38^, ^40^ This study found that Bmi‐1 downregulated RAAS activation by inhibiting ROS accumulation and DDR in cardiovascular tissues. As a key molecule in physiological and pathological mechanisms of the heart, Ang II affects energy metabolism and the onset and the progression of senescence in cardiomyocytes.^2^ However, the functions of Bmi‐1 in preventing SA‐PCH are largely unknown. This study observed that *Bmi‐1* deficiency caused cardiac dysfunction, ultrastructure damage, aging and Ang II‐dependent PCH including hypertrophy, fibrosis and inflammation. Furthermore, Ang II exogenous supplementation stimulates the development of PCH observed in aged mice.^1^ Ang II inhibition protects end organs from damage.^2^, ^40^ This study also showed that *Bmi‐1* deficiency aggravated Ang II‐induced PCH and cardiac dysfunction. In contrast, *Bmi‐1* overexpression in the myocardium and cardiomyocytes ameliorated Ang II‐induced PCH and cardiac dysfunction. Thus, Bmi‐1 prevented the development of SA‐PCH and maintained cardiac function. However, this study has not clarified whether Bmi‐1 directly induces autophagy, which needs further study.

Growing evidence suggests that decreased autophagy leads to cardiac hypertrophy.^10^ When accompanied by aging, autophagy cannot perform its key function in preventing the decline of heart function during cardiac aging, due to the decline of autophagic degradation.^3^ Several reports reveal that Ang II increases autophagy in PCH mainly through mediating AT1R and AT2R, affecting oxidative stress, microRNAs and mammalian target of rapamycin (mTOR).^41^ Previous study finds that GATA4 protein is mainly degraded by p62‐mediated selective autophagy.^8^ However, there are also articles arguing that promoting degradation of GATA4 protein by carboxyl terminus of Hsp70‐interacting protein contributes to hyperglycemic cardiotoxicity.^42^ Excessive ROS promotes GATA4 protein for proteasome‐dependent degradation.^43^ This study found that the degradation of GATA4 by Bmi‐1 mainly depended on autophagy rather than proteasome. Although autophagic flux increased after Ang II treatment in WT and *Bmi‐1* deficiency hearts or MECs, GATA4 protein remained elevated. In *Bmi‐1* deficiency hearts or MECs, autophagic flux was lower than in WT. However, levels of GATA4 protein and transcription of its downstream molecules were higher than WT after induction with Ang II. *Bmi‐1* overexpression in the heart further promoted autophagic flux and GATA4 degradation and downregulated GATA4‐dependent downstream molecules. These results were confirmed in human cells. Thus, Bmi‐1 promoted the autophagic degradation of GATA4 protein in Ang II‐induced PCH. In CMA, the common KFERQ‐like motif in targeted proteins provided the ‘bait’ to identify HSC70 as the cytosolic chaperone that, upon binding that region, targeted proteins for lysosomal degradation.^44^ Our results demonstrated that p62‐mediated lysosomal degradation of GATA4 was not HSC70‐mediated CMA.

GATA4 is a transcription factor that transcribes several critical structural and regulatory foetal genes in embryonic and adult cardiomyocytes. It is also a novel marker of aging regulating SASP and senescence. Previous observations show that ATM and ATR, as DDR kinases, increase GATA4 protein during cellular senescence, suggesting that ATM and ATR might inhibit the selective autophagy of GATA4.^8, 21^ As a substrate of ATM and a deubiquitinase, USP28 is phosphorylated and activated after DNA damage. As a signal transducer, USP28 deubiquitinates GATA4 or its associated factors and prevents GATA4 degradation by p62‐mediated selective autophagy.^8^, ^21^, ^45^ Whether E3 ubiquitin ligases could be an opposite signal transducer, ubiquitinate GATA4 and promote GATA4 degradation by p62‐mediated selective autophagy is unknown. Bmi‐1 maintains mitochondrial function and redox homeostasis, preventing DNA damage and DDR to delay cell aging.^11^, ^12^, ^14^ Following DNA damage, Bmi‐1 tethers RING1B to damaged DNA for ubiquitination.^46^ Bmi‐1 is indispensable for increasing ubiquitination by E3 ubiquitin‐ligase RING1B.^19^, ^47^ A previous study demonstrated that the proteasomal degradation of Top2 is dependent on E3 ubiquitin‐ligase Bmi‐1‐RING1A.^18^ This study found that Bmi‐1‐RING1B combined with GATA4 and promoted the ubiquitination of GATA4 and GATA4 degradation by p62‐mediated selective autophagy. Thus, we propose that Bmi‐1‐RING1B is a signal transducer similar to USP28 in crosstalk between GATA4 and p62, because p62 primarily recognises ubiquitinated substrates. However, whether Bmi‐1 interacts with USP28 and how it regulates the balance between Bmi‐1‐RING1B and USP28 on GATA4 stability remains to be investigated. With ubiquitin assay, we found Bmi‐1 overexpression without RING1B overexpression promoted GATA4 ubiquitination. This effect was higher than RING1B overexpression without Bmi‐1 overexpression, but lower than Bmi‐1 and RING1B co‐overexpression. A recent study found that Bmi‐1 associated with the SCF ubiquitination complex through its N terminus and with phosphorylation by an IKKα/β‐dependent pathway, resulting in the ubiquitination of IkBα.^48^ It suggested that Bmi‐1 might form with other ubiquitination complexes such as SCF to promote the ubiquitination of GATA4, which needs further study to determine.

A previous observation finds that with aging, the protein expression levels of NF‐κB‐p65 and p‐p65 (Ser536) increase in rats.^49^ With aging, increased Ang II activates NF‐κB by phosphorylating IκB‐α and p65. Increased p‐p65 at Ser536 is mediated by the elevated phosphorylation of IκB‐α.^49^ Consistent with them, our results suggested that p‐p65 (Ser536) could be increased by the elevated phosphorylation of IκB‐α in aging hearts. Moreover, a previous study demonstrates that GATA4 activates NF‐κB and subsequently promotes phosphorylation of p65 through upregulating the mRNA levels of *TRAF3IP2*.^8^ Consistent with it, this study suggested that p‐p65 (Ser536) could be increased by the elevated GATA4 and *TRAF3IP2* in aging hearts. We also found the mRNA level of *p65* (*RelA*) was increased in hearts of aged mice compared to young mice. We will investigate whether *p65* (*RelA*) was transcriptionally activated by GATA4, and/or transcriptionally inhibited by Bmi‐1 and polycomb repressive complexes in the follow‐up study.

Aging and associated cardiac hypertrophy are regulated by several core metabolic sensors, including AMP‐activated protein kinase (AMPK) and mammalian target of rapamycin (mTOR).^1^ In this process, activation of AMPK by metformin and inhibition of mTOR by rapamycin protects the heart from cardiac hypertrophy by triggering autophagy induction.^1^, ^50^ To determine whether *Bmi‐1* deficiency causes disorders of GATA4‐selective autophagy in cardiomyocytes, and GATA4‐dependent cell hypertrophy, senescence and inflammation, mice and MECs were treated with Ang II to induce hypertrophy and rescued with metformin or rapamycin. We found metformin and rapamycin promoted p62‐mediated GATA4‐selective autophagy and ameliorated GATA4‐dependent SA‐PCH and SASP in WT and *Bmi‐1*‐deficient mice. However, the ameliorating effects of metformin and rapamycin were lower in *Bmi‐1*‐deficient mice than WT mice due to decreased GATA4 ubiquitination.

Several lines of evidence demonstrate that reduced autophagic activity correlates with aging in heart tissues.^36^, ^51^ Our studies on human myocardial tissues of different ages indicated that the accumulation of GATA4 and ANP in human hearts accompanied by cell senescence could be caused by downregulated Bmi‐1‐RING1B‐dependent autophagic degradation. Age does not completely determine the degree of cellular senescence;^16^, ^52^, ^53^ thus p16 protein level in human myocardial tissues was chosen as the main aging evaluation marker. P16 is transcriptionally activated and accumulates in irreversibly aging cells and leads to aging‐associated impaired function and diseases.^52^, ^53^ In this study, we found that p16 positively correlated with ANP, GATA4 and age, and along with increased p16 protein levels, ANP‐, BNP‐, GATA4‐positive cells or areas increased. However, p16 negatively correlated with LC3B, and LC3B‐positive cells or areas decreased in human myocardial tissues. The study of human myocardial samples has a limitation. The expression levels of ANP and BNP reflect the status of HF and are not attributed to aging‐induced hypertrophy. Because of the limitation of medical ethics, we could not get the physiological data including heart weight, left ventricular function, wall thickness and plasma samples of autopsied donors before their death for strengthening our opinions.

Previous observations demonstrated that Bmi‐1‐(1‐102) is sufficient for stably interacting with RING1B and stimulating the E3 ligase activity of RING1B.^19^ RING1B‐(5‐115) encompasses a region of the substrate and is required for mono‐ubiquitination E3 ligase activity.^19^ The N‐ and C‐terminal regions flanking the central ring motif (residues 50–103 of RING1B and 17–72 of Bmi‐1) interact and form a short four‐helix bundle involving only 1–2 turns.^18^ Bmi‐1 is a member of the PRC‐1 complex and includes N‐terminal ring finger and helix‐turn‐helix domains.^54^ Several lines of evidence support that Bmi‐1 combines with and regulates the ubiquitination of GATA3 or GATA6 via the N‐terminal ring finger domain (residues 17–56 of Bmi‐1 in mouse).^29^, ^54^ GATA6 combines with Bmi‐1 through a C‐terminal domain and/or zinc finger region in mice.^29^ We propose that the same domain is involved in interaction with Bmi‐1 with GATA4, GATA3 and GATA6. Our analysis found that a similar domain was labelled as a zinc finger GATA4 (residues 214–320), GATA3 (residues 261–367) and GATA6 (residues 382–488) in mice. A similar domain was also labelled as a zinc finger in GATA4 (residues 215–321), GATA3 (residues 261–367) and GATA6 (residues 388–494) in humans. Consistent with this analysis, Bmi‐1 combined with RING1B (residues 1–179) and C‐terminus of GATA4 (residues 206–443 including zinc finger domains) through residues 1–95 including a RING‐HC‐finger. RING1B combined with C‐terminus of GATA4 (residues 206–443 including zinc finger domains) through the C‐terminus (residues 180–336). We also found residues 1–95 of Bmi‐1 and full length of RING1B could promote ubiquitination and autophagy degradation of GATA4 for preventing SA‐PCH. However, Bmi‐1‐FL has a better ubiquitination modification effect on GATA4 than Bmi‐1‐1‐95 residues, so we constructed the full length of *Bmi‐1* and *RING1B* into AAV9. Whether there are other residues of Bmi‐1 that play a role in GATA4 ubiquitin modification still needs further study. The stapled peptides can stabilise the binding of protein and protein on the premise of identifying the key domain of protein–protein interaction. Because binding is a dynamic process, the stapled peptides' role is to make the binding state last longer and dissociation more difficult.^55^, ^56^ The combined domains between Bmi‐1‐RING1B and GATA4 in aging cardiomyocytes could be therapeutic targets for identifying stapled peptides to promote the combination of Bmi‐1‐RING1B with GATA4 and the ubiquitination of GATA4 to prevent SA‐PCH and HF. Previous reports considered AAV9‐*CMV* viral packaging as an efficient and safe tool for cardiac gene transfer due to its consistent transduction efficiency and established cardiac tropism.^30^, ^31^, ^32^ It has been used in multiple preclinical studies, for example, in the first‐in‐human cardiac gene therapy trial because of its robust expression.^33^, ^34^ Until now, no large animal study is to detect the effectiveness of rationally designed cardiac‐specific promoter *cTnT* for gene therapy.^34^ Because of the large base number of *Bmi‐1‐RING1B* complex, we used a universal promoter *CMV* to better express *Bmi‐1‐RING1B* complex rather than *cTnT*. Our results demonstrated that AAV9‐*CMV‐Bmi‐1‐RING1B* injection significantly increased the protein level of Bmi‐1 and RING1B in heart and prevented from SA‐PCH.

Autophagic degradation usually takes place in the cytoplasm.^8^ This study found GATA4 and Bmi‐1 or Bmi‐1 and RING1B combined mainly in the nucleus; however, the combination of GATA4 and RING1B increased in the cytoplasm. This study also showed GATA4 autophagic degradation happened in the cytoplasm. A line of evidence demonstrates that nuclear protein Lamin B1 undergoes autophagic degradation in the cytoplasm through a nucleus‐to‐cytoplasm transport process by nuclear membrane blebbing.^57^ Whether the same method is used for GATA4 transport and degradation needs further study.

Finally, our study demonstrated that Bmi‐1‐RING1B maintained cardiac function and prevented SA‐PCH by promoting selective autophagy for degrading GATA4. The combined domains between Bmi‐1‐RING1B and GATA4 in aging cardiomyocytes could be therapeutic targets for identifying small molecular peptides in clinical applications to promote the combination of Bmi‐1‐RING1B with GATA4 and the ubiquitination of GATA4 to prevent SA‐PCH and HF. AAV9‐*CMV*‐*Bmi‐1‐RING1B* treatment significantly attenuated GATA4‐dependent SA‐PCH through promoting GATA4 autophagic degradation, which could be used for translational gene therapy.

## CONFLICT OF INTERESTS

The authors declare no competing interests.

## Supporting information

Supplementary Information 1: Figures S1–S13

Supplementary Information 2: Figures S1–S13 Legends

Supplementary Information 4: Complete Materials and Methods

Supplementary Information 5: Table S1

Supplementary Information 6: Alignment of GATA3, GATA4 and GATA6 in Human

Supplementary Information 7: Alignment of GATA3, GATA4 and GATA6 in Mouse

Supplementary Information 8: Alignment of GATA4 Protein and motif‐KFERQ in Human or Mouse

Supplementary Information 9: Original Blots in Figures S14–S17

Supplementary Information 10: The Physiological Data of Autopsied Donors

## References

[1] Tang X , Chen XF , Wang NY , et al. SIRT2 acts as a cardioprotective deacetylase in pathological cardiac hypertrophy. Circulation. 2017;136:2051‐2067. 28947430 10.1161/CIRCULATIONAHA.117.028728 PMC5698109

[2] Conti S , Cassis P , Benigni A . Aging and the renin‐angiotensin system. Hypertension (Dallas, Tex: 1979). 2012;60:878‐883. 10.1161/HYPERTENSIONAHA.110.155895 22926952

[3] Miyamoto S . Autophagy and cardiac aging. Cell Death Differ. 2019;26:653‐664. 30692640 10.1038/s41418-019-0286-9 PMC6460392

[4] Zhou Y , Wang L , Vaseghi HR , et al. Bmi1 is a key epigenetic barrier to direct cardiac reprogramming. Cell Stem Cell. 2016;18:382‐395. 26942853 10.1016/j.stem.2016.02.003 PMC4779178

[5] Oka T , Maillet M , Watt AJ , et al. Cardiac‐specific deletion of Gata4 reveals its requirement for hypertrophy, compensation, and myocyte viability. Circ Res. 2006;98:837‐845. 16514068 10.1161/01.RES.0000215985.18538.c4

[6] Yamamura S , Izumiya Y , Araki S , et al. Cardiomyocyte Sirt (sirtuin) 7 ameliorates stress‐induced cardiac hypertrophy by interacting with and deacetylating GATA4. Hypertension (Dallas, Tex: 1979). 2020;75:98‐108. 10.1161/HYPERTENSIONAHA.119.13357 31735083

[7] Kim HN , Chang J , Shao L , et al. DNA damage and senescence in osteoprogenitors expressing Osx1 may cause their decrease with age. Aging Cell. 2017;16:693‐703. 28401730 10.1111/acel.12597 PMC5506444

[8] Kang C , Xu X , Martin TD , et al. The DNA damage response induces inflammation and senescence by inhibiting autophagy of GATA4. Science. 2015;349:aaa5612. 26404840 10.1126/science.aaa5612 PMC4942138

[9] Taneike M , Yamaguchi O , Nakai A , et al. Inhibition of autophagy in the heart induces age‐related cardiomyopathy. Autophagy. 2010;6:600‐606. 20431347 10.4161/auto.6.5.11947

[10] Xie X , Bi HL , Lai S , et al. The immunoproteasome catalytic β5i subunit regulates cardiac hypertrophy by targeting the autophagy protein ATG5 for degradation. Sci Adv. 2019;5:eaau0495. 31086810 10.1126/sciadv.aau0495 PMC6506244

[11] Jin J , Lv X , Chen L , et al. Bmi‐1 plays a critical role in protection from renal tubulointerstitial injury by maintaining redox balance. Aging Cell. 2014;13:797‐809. 24915841 10.1111/acel.12236 PMC4331754

[12] Liu J , Cao L , Chen J , et al. Bmi1 regulates mitochondrial function and the DNA damage response pathway. Nature. 2009;459:387‐392. 19404261 10.1038/nature08040 PMC4721521

[13] Zhang HW , Ding J , Jin JL , et al. Defects in mesenchymal stem cell self‐renewal and cell fate determination lead to an osteopenic phenotype in Bmi‐1 null mice. J Bone Miner Res. 2010;25:640‐652. 19653817 10.1359/jbmr.090812

[14] Xie C , Jin J , Lv X , Tao J , Wang R , Miao D . Anti‐aging effect of transplanted amniotic membrane mesenchymal stem cells in a premature aging model of Bmi‐1 deficiency. Sci Rep. 2015;5:13975. 26370922 10.1038/srep13975 PMC4570627

[15] Jin J , Tao J , Gu X , Yu Z , Wang R , Zuo G , Li Q , Lv X , Miao D . P16 INK4a Deletion Ameliorated Renal Tubulointerstitial Injury in a Stress‐induced Premature Senescence Model of Bmi‐1 Deficiency. Scientific Reports. 2017;7(1): 10.1038/s41598-017-06868-8 PMC5548892 28790310

[16] Chen H , Chen H , Liang J , et al. TGF‐β1/IL‐11/MEK/ERK signaling mediates senescence‐associated pulmonary fibrosis in a stress‐induced premature senescence model of Bmi‐1 deficiency. Exp Mol Med. 2020;52:130‐151. 31959867 10.1038/s12276-019-0371-7 PMC7000795

[17] Bentley ML , Corn JE , Dong KC , Phung Q , Cheung TK , Cochran AG . Recognition of UbcH5c and the nucleosome by the Bmi1/Ring1b ubiquitin ligase complex. EMBO J. 2011;30:3285‐3297. 21772249 10.1038/emboj.2011.243 PMC3160663

[18] Buchwald G , van der Stoop P , Weichenrieder O , Perrakis A , van Lohuizen M , Sixma TK . Structure and E3‐ligase activity of the Ring‐Ring complex of polycomb proteins Bmi1 and Ring1b. EMBO J. 2006;25:2465‐2474. 16710298 10.1038/sj.emboj.7601144 PMC1478191

[19] Li Z , Cao R , Wang M , Myers MP , Zhang Y , Xu RM . Structure of a Bmi‐1‐Ring1B polycomb group ubiquitin ligase complex. J Biol Chem. 2006;281:20643‐20649. 16714294 10.1074/jbc.M602461200

[20] Chan HL , Morey L . Emerging Roles for polycomb‐group proteins in stem cells and cancer. Trends Biochem Sci. 2019;44:688‐700. 31085088 10.1016/j.tibs.2019.04.005

[21] Kang C , Elledge SJ . How autophagy both activates and inhibits cellular senescence. Autophagy. 2016;12:898‐899. 27129029 10.1080/15548627.2015.1121361 PMC4854549

[22] Chen G , Zhang Y , Yu S , Sun W , Miao D . Bmi1 overexpression in mesenchymal stem cells exerts antiaging and antiosteoporosis effects by inactivating p16/p19 signaling and inhibiting oxidative stress. Stem Cells. 2019;37:1200‐1211. 30895687 10.1002/stem.3007 PMC6851636

[23] Sun H , Qiao W , Cui M , Yang C , Wang R , Goltzman D , Jin J , Miao D . The Polycomb Protein Bmi1 Plays a Crucial Role in the Prevention of 1,25(OH) 2 D Deficiency‐Induced Bone Loss. Journal of Bone and Mineral Research. 2020;35(3):583–595. 10.1002/jbmr.3921 31725940

[24] Palomer X , Alvarez‐Guardia D , Rodriguez‐Calvo R , et al. TNF‐alpha reduces PGC‐1 alpha expression through NF‐kappa B and p38 MAPK leading to increased glucose oxidation in a human cardiac cell model. Cardiovasc Res. 2009;81:703‐712. 19038972 10.1093/cvr/cvn327

[25] Davidson MM , Nesti C , Palenzuela L , et al. Novel cell lines derived from adult human ventricular cardiomyocytes. J Mol Cell Cardiol. 2005;39:133‐147. 15913645 10.1016/j.yjmcc.2005.03.003

[26] Jin JL , Zhao YM , Tan X , Guo C , Yang ZJ , Miao DS . An improved transplantation strategy for mouse mesenchymal stem cells in an acute myocardial infarction model. PLoS One. 2011;6. 10.1371/journal.pone.0021005 PMC3117862 21698117

[27] Yu T , Guo F , Yu Y , et al. Fusobacterium nucleatum promotes chemoresistance to colorectal cancer by modulating autophagy. Cell. 2017;170:548‐563. e516. 28753429 10.1016/j.cell.2017.07.008 PMC5767127

[28] Zhang H , Zhang Y , Zhu X , et al. DEAD box protein 5 inhibits liver tumorigenesis by stimulating autophagy via interaction with p62/SQSTM1. Hepatology. 2019;69:1046‐1063. 30281815 10.1002/hep.30300 PMC6411283

[29] Lavial F , Bessonnard S , Ohnishi Y , et al. Bmi1 facilitates primitive endoderm formation by stabilizing Gata6 during early mouse development. Genes Dev. 2012;26:1445‐1458. 22713603 10.1101/gad.188193.112 PMC3403013

[30] Chen BD , He CH , Chen XC , et al. Targeting transgene to the heart and liver with AAV9 by different promoters. Clin Exp Pharmacol Physiol. 2015;42:1108‐1117. 26173818 10.1111/1440-1681.12453

[31] Liu Y , Zhou K , Li J , et al. In mice subjected to chronic stress, exogenous cBIN1 preserves calcium‐handling machinery and cardiac function. JACC Basic Transl Sci. 2020;5:561‐578. 32613144 10.1016/j.jacbts.2020.03.006 PMC7315191

[32] Zhao Q , Liu F , Zhao Q , et al. Constitutive activation of ERK1/2 signaling protects against myocardial ischemia via inhibition of mitochondrial fragmentation in the aging heart. Ann Transl Med. 2021;9:479. 33850876 10.21037/atm-21-503 PMC8039677

[33] Jessup M , Greenberg B , Mancini D , et al. Calcium upregulation by percutaneous administration of gene therapy in cardiac disease (CUPID): a phase 2 trial of intracoronary gene therapy of sarcoplasmic reticulum Ca2+‐ATPase in patients with advanced heart failure. Circulation. 2011;124:304‐313. 21709064 10.1161/CIRCULATIONAHA.111.022889 PMC5843948

[34] Bezzerides VJ , Prondzynski M , Carrier L , Pu WT . Gene therapy for inherited arrhythmias. Cardiovasc Res. 2020;116:1635‐1650. 32321160 10.1093/cvr/cvaa107 PMC7341167

[35] Schafer S , Viswanathan S , Widjaja AA , et al. IL‐11 is a crucial determinant of cardiovascular fibrosis. Nature. 2017;552:110‐115. 29160304 10.1038/nature24676 PMC5807082

[36] Shirakabe A , Ikeda Y , Sciarretta S , Zablocki DK , Sadoshima J . Aging and autophagy in the heart. Circ Res. 2016;118:1563‐1576. 27174950 10.1161/CIRCRESAHA.116.307474 PMC4869999

[37] Keidar S , Kaplan M , Gamliel‐Lazarovich A . ACE2 of the heart: from angiotensin I to angiotensin (1‐7). Cardiovasc Res. 2007;73:463‐469. 17049503 10.1016/j.cardiores.2006.09.006

[38] de Cavanagh EM , Inserra F , Ferder L . Angiotensin II blockade: how its molecular targets may signal to mitochondria and slow aging. Coincidences with calorie restriction and mTOR inhibition. Am J Physiol Heart Circ Physiol. 2015;309:H15‐44. 25934099 10.1152/ajpheart.00459.2014

[39] Impellizzeri D , Esposito E , Attley J , Cuzzocrea S . Targeting inflammation: new therapeutic approaches in chronic kidney disease (CKD). Pharmacol Res. 2014;81:91‐102. 24602801 10.1016/j.phrs.2014.02.007

[40] Oudit GY , Kassiri Z , Patel MP , et al. Angiotensin II‐mediated oxidative stress and inflammation mediate the age‐dependent cardiomyopathy in ACE2 null mice. Cardiovasc Res. 2007;75:29‐39. 17499227 10.1016/j.cardiores.2007.04.007

[41] Zhou L , Ma B , Han X . The role of autophagy in angiotensin II‐induced pathological cardiac hypertrophy. J Mol Endocrinol. 2016;57:R143‐R152. 27620875 10.1530/JME-16-0086

[42] Kobayashi S , Mao K , Zheng H , et al. Diminished GATA4 protein levels contribute to hyperglycemia‐induced cardiomyocyte injury. J Biol Chem. 2007;282:21945‐21952. 17525155 10.1074/jbc.M703048200

[43] Li T , Zhang X , Jiang K , Liu J , Liu Z . Dural effects of oxidative stress on cardiomyogenesis via Gata4 transcription and protein ubiquitination. Cell Death Dis. 2018;9:246. 29445146 10.1038/s41419-018-0281-y PMC5833852

[44] Kaushik S , Cuervo AM . The coming of age of chaperone‐mediated autophagy. Nat Rev Mol Cell Biol. 2018;19:365‐381. 29626215 10.1038/s41580-018-0001-6 PMC6399518

[45] Mazzucco AE , Smogorzewska A , Kang C , et al. Genetic interrogation of replicative senescence uncovers a dual role for USP28 in coordinating the p53 and GATA4 branches of the senescence program. Genes Dev. 2017;31:1933‐1938. 29089421 10.1101/gad.304857.117 PMC5710139

[46] Bhattacharya R , Mustafi SB , Street M , Dey A , Dwivedi SK . Bmi‐1: at the crossroads of physiological and pathological biology. Genes Dis. 2015;2:225‐239. 26448339 10.1016/j.gendis.2015.04.001 PMC4593320

[47] Alchanati I , Teicher C , Cohen G , et al. The E3 ubiquitin‐ligase Bmi1/Ring1A controls the proteasomal degradation of Top2alpha cleavage complex – a potentially new drug target. PLoS One. 2009;4:e8104. 19956605 10.1371/journal.pone.0008104 PMC2779455

[48] Okuyama Y , Tanaka Y , Jiang JJ , et al. Bmi1 regulates IκBα degradation via association with the SCF complex. J Immunol. 2018;201:2264‐2272. 30209188 10.4049/jimmunol.1701223

[49] Kim JM , Heo HS , Ha YM , et al. Mechanism of Ang II involvement in activation of NF‐kappaB through phosphorylation of p65 during aging. Age. 2012;34:11‐25. 21318332 10.1007/s11357-011-9207-7 PMC3260361

[50] Li Z , Song Y , Liu L , et al. miR‐199a impairs autophagy and induces cardiac hypertrophy through mTOR activation. Cell Death Differ. 2017;24:1205‐1213. 26160071 10.1038/cdd.2015.95 PMC5520159

[51] Ren J , Zhang Y . Targeting autophagy in aging and aging‐related cardiovascular diseases. Trends Pharmacol Sci. 2018;39:1064‐1076. 30458935 10.1016/j.tips.2018.10.005 PMC6251315

[52] Baker DJ , Childs BG , Durik M , et al. Naturally occurring p16(Ink4a)‐positive cells shorten healthy lifespan. Nature. 2016;530:184‐189. 26840489 10.1038/nature16932 PMC4845101

[53] Naylor RM , Baker DJ , van Deursen JM . Senescent cells: a novel therapeutic target for aging and age‐related diseases. Clin Pharmacol Ther. 2013;93:105‐116. 23212104 10.1038/clpt.2012.193 PMC4051295

[54] Hosokawa H , Kimura MY , Shinnakasu R , et al. Regulation of Th2 cell development by polycomb group gene bmi‐1 through the stabilization of GATA3. J Immunol. 2006;177:7656‐7664. 17114435 10.4049/jimmunol.177.11.7656

[55] Tan YS , Lane DP , Verma CS . Stapled peptide design: principles and roles of computation. Drug Discov Today. 2016;21:1642‐1653. 27326912 10.1016/j.drudis.2016.06.012

[56] Moiola M , Memeo MG , Quadrelli P . Stapled peptides – a useful improvement for peptide‐based drugs. Molecules. 2019;24. 10.3390/molecules24203654 PMC6832507 31658723

[57] Dou Z , Xu C , Donahue G , et al. Autophagy mediates degradation of nuclear lamina. Nature. 2015;527:105‐109. 26524528 10.1038/nature15548 PMC4824414

