# Corrigendum to ‘Bmi‐1‐RING1B prevents GATA4‐dependent senescence‐associated pathological cardiac hypertrophy by promoting autophagic degradation of GATA4’

**DOI:** 10.1002/ctm2.1748

**Published:** 2024-06-25

Chen H, Zhou J, Chen H, et al. Bmi‐1‐RING1B prevents GATA4‐dependent senescence‐associated pathological cardiac hypertrophy by promoting autophagic degradation of GATA4. *Clin Transl Med*. 2022;12(4):e574. https://doi.org/10.1002/ctm2.574

In this article, the authors have just realized the wrong usage of WGA image in the group of *Bmi‐1^−/−^* mice administered with Ang II and MF, using instead the image of WGA from the *Bmi‐1^−/−^* group in Figure 3F. The corrected Figure 3 is as follows.

**FIGURE 3:**
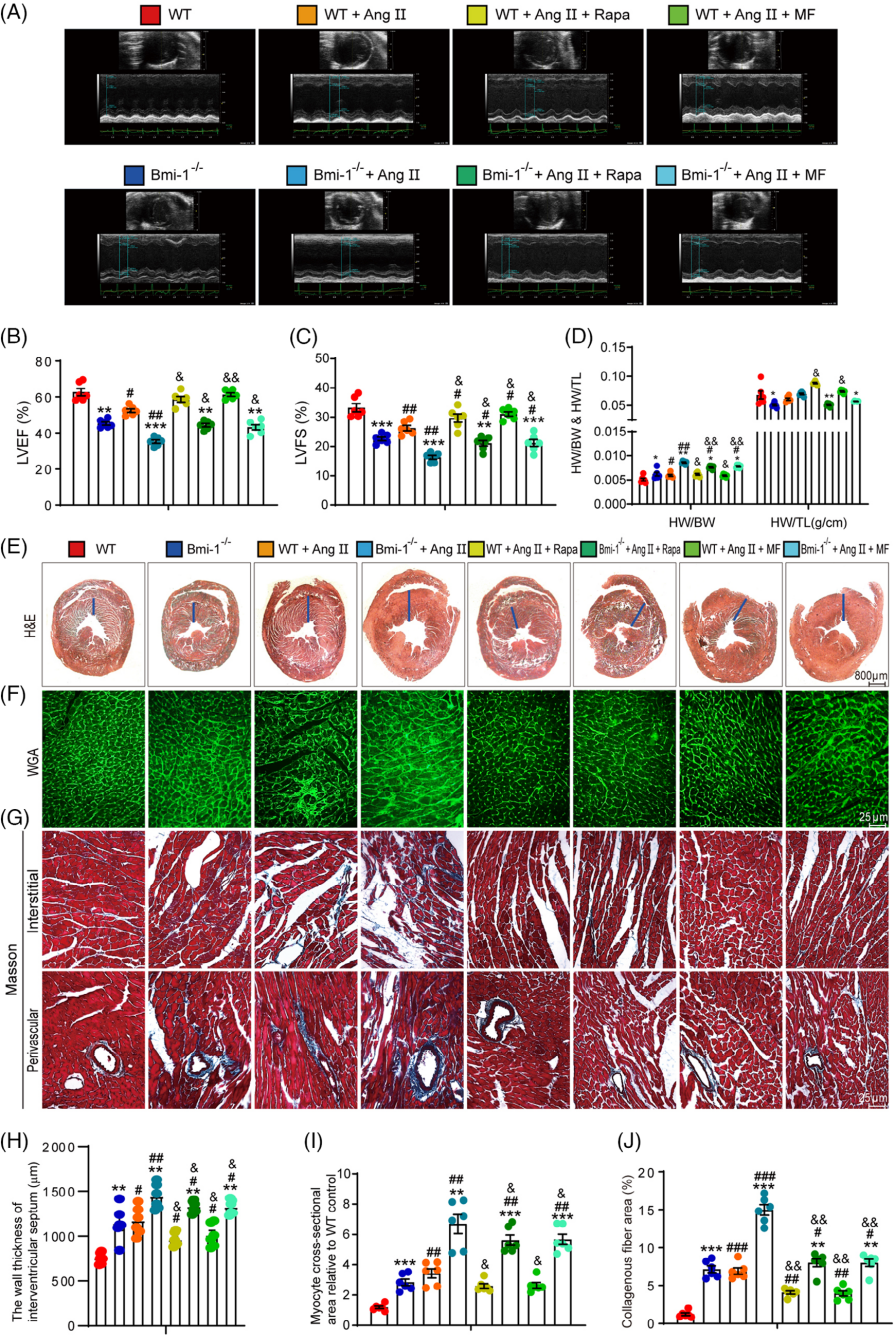


Moreover, the authors have just realized the wrong usage of H&E image in the group of WT mice administered with Ang II in Figure 5F, using instead the slices from the *Bmi‐1^−/−^* group in Figure 3E. The updated Figure 5 is provided.

**FIGURE 5:**
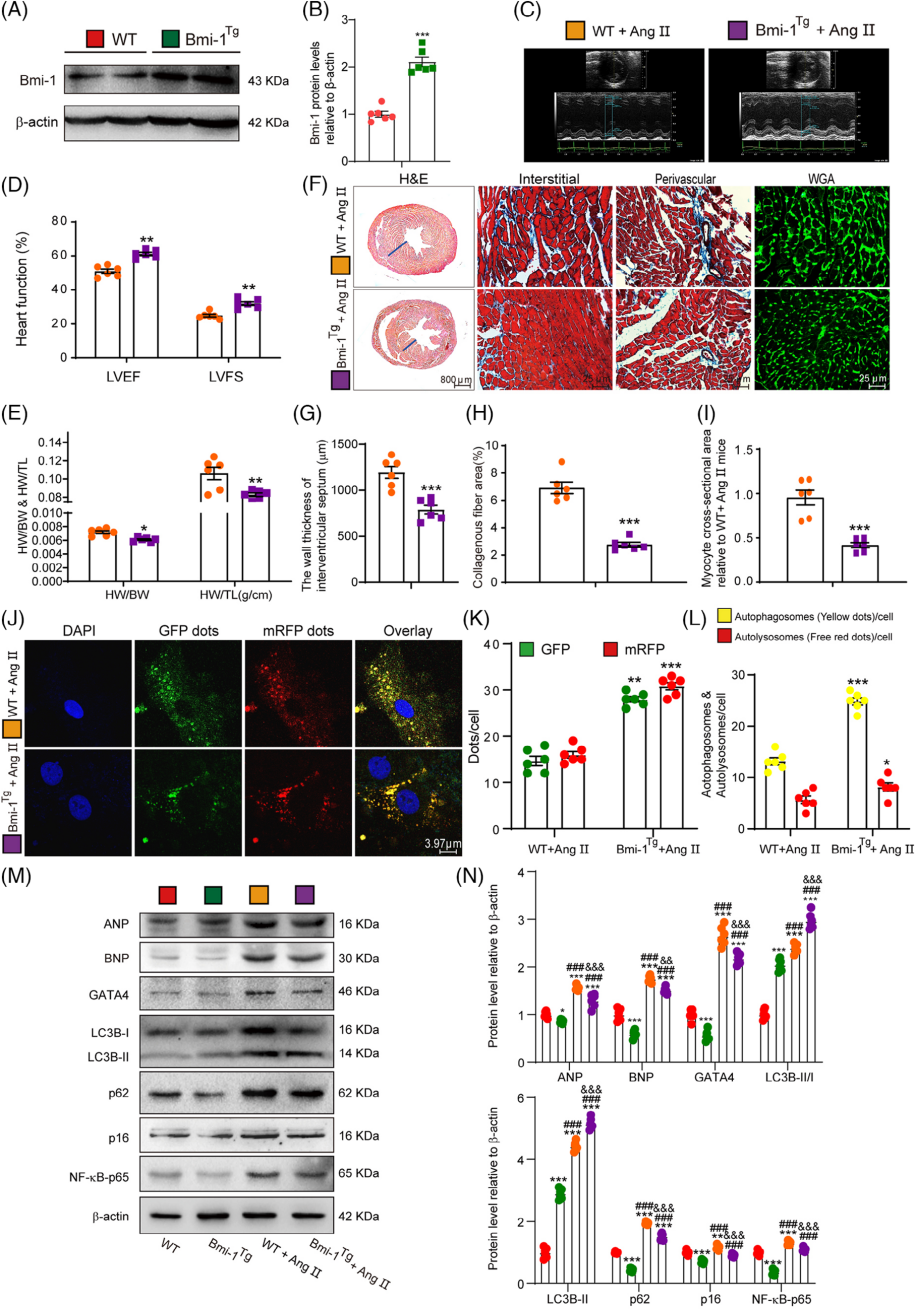


The authors apologize for these errors and for any inconvenience caused and appreciate your understanding and support. The corrections have no impact on the experimental outcome or conclusions.

