# Supplementary material for: Bmi‐1‐RING1B prevents GATA4‐dependent senescence‐associated pathological cardiac hypertrophy by promoting autophagic degradation of GATA4
Source: Clin Transl Med. 2022 Apr 7;12(4):e574. doi: 10.1002/ctm2.574 (PMC8989148; doi:10.1002/ctm2.574)
Supplement: Supplementary file 1 — Supplementary Information 1: Figures S1–S13 [file CTM2-12-e574-s006.pdf]

Figure S1

A.

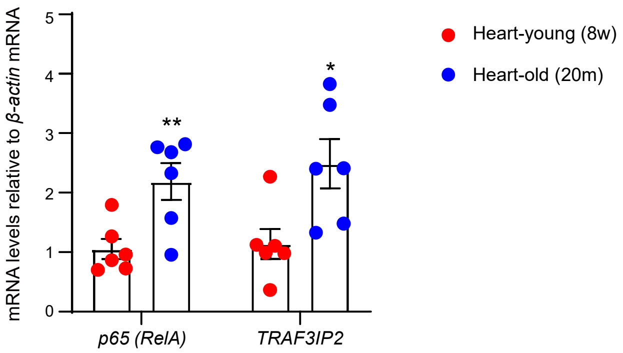

mRNA levels relative to β-actin mRNA
5
4
3
2
1
0
**
*
p65 (RelA)
TRAF3IP2
Heart-young (8w)
Heart-old (20m)

Figure S2

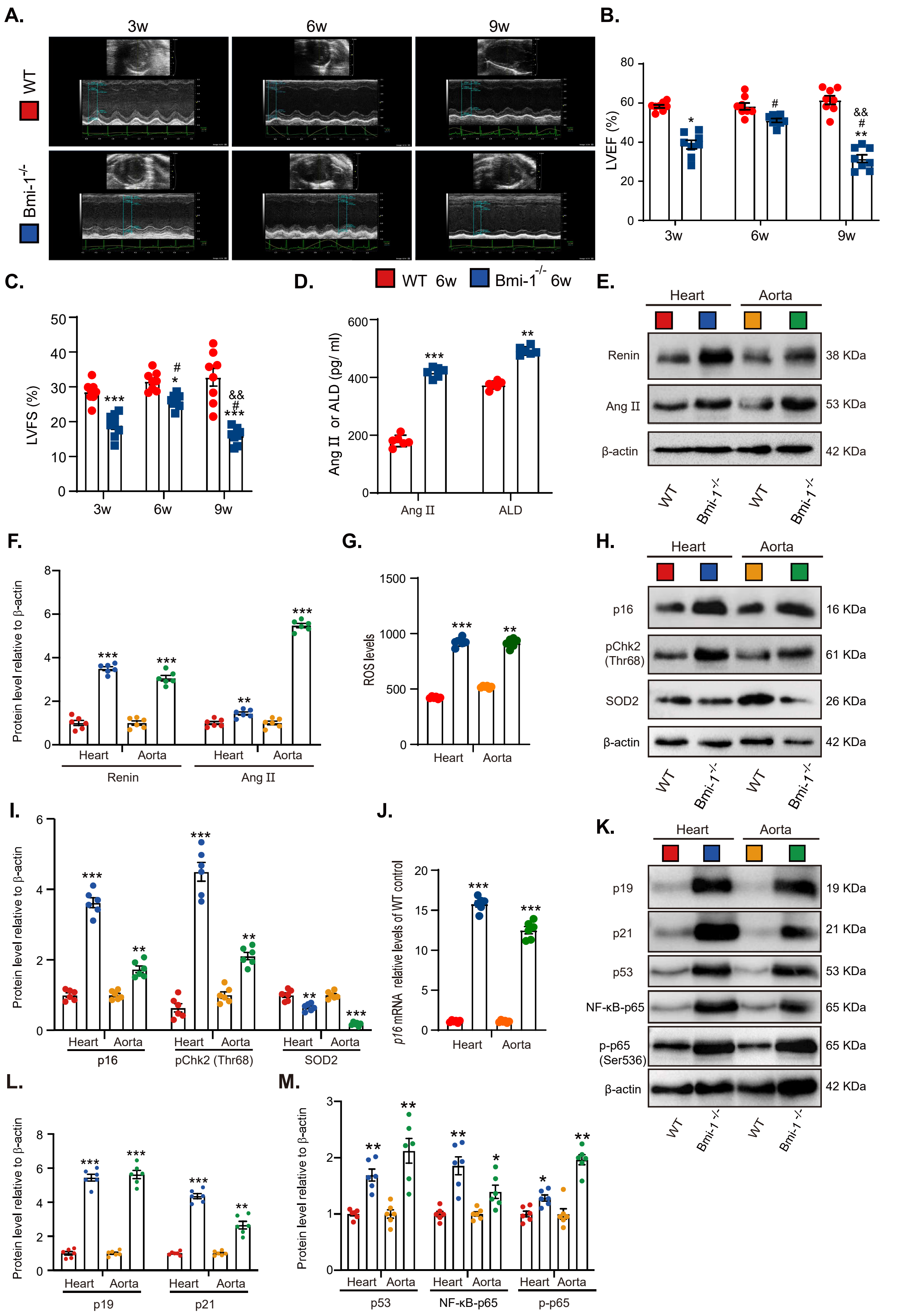

A.
3w
6w
9w
WT
Bmi-1-/-
B.
LVEF (%)
3w
6w
9w
C.
LVFS (%)
3w
6w
9w
D.
WT 6w
Bmi-1-/- 6w
Ang II or ALD (pg/ ml)
Ang II
ALD
E.
Heart
Aorta
Renin
38 KDa
Ang II
53 KDa
β-actin
42 KDa
WT
Bmi-1-/-
F.
Protein level relative to β-actin
Heart
Aorta
Renin
Ang II
G.
ROS levels
Heart
Aorta
H.
p16
16 KDa
pChk2 (Thr68)
61 KDa
SOD2
26 KDa
β-actin
42 KDa
I.
p16
pChk2 (Thr68)
SOD2
J.
p16 mRNA relative levels of WT control
K.
p19
19 KDa
p21
21 KDa
p53
53 KDa
NF-κB-p65
65 KDa
p-p65 (Ser536)
65 KDa
β-actin
42 KDa
L.
p19
p21
M.
p53
NF-κB-p65
p-p65

Figure S3

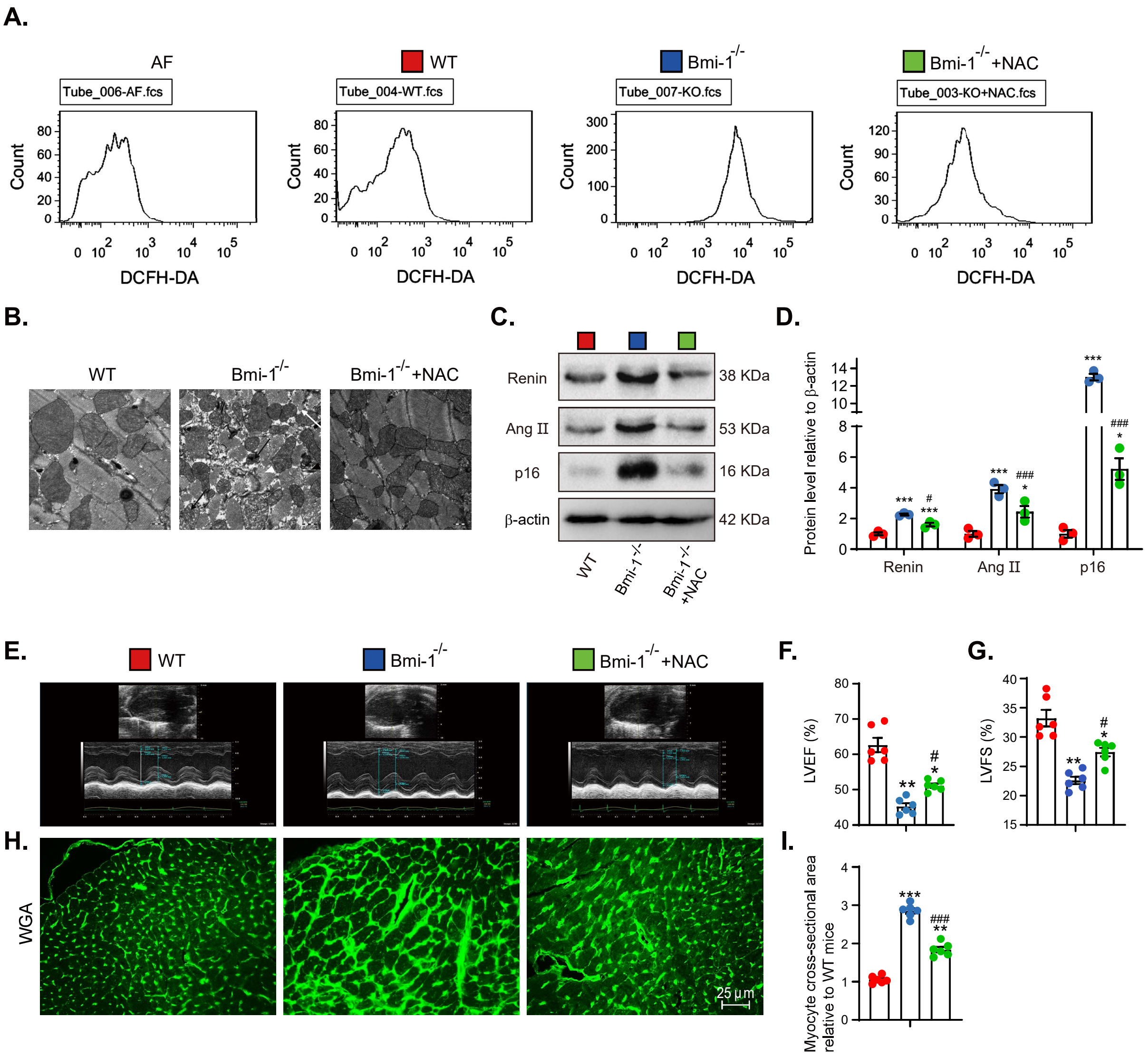
A.
AF
WT
Bmi-1-/-
Bmi-1-/- +NAC
Tube_006-AF.fcs
Tube_004-WT.fcs
Tube_007-KO.fcs
Tube_003-KO+NAC.fcs
Count
DCFH-DA
B.
WT
Bmi-1-/-
Bmi-1-/- +NAC
C.
Renin
Ang II
p16
β-actin
38 KDa
53 KDa
16 KDa
42 KDa
WT
Bmi-1-/-
Bmi-1-/- +NAC
D.
Protein level relative to β-actin
Renin
Ang II
p16
E.
WT
Bmi-1-/-
Bmi-1-/- +NAC
F.
LVEF (%)
G.
LVFS (%)
H.
WGA
25 μm
I.
Myocyte cross-sectional area relative to WT mice

Figure S4

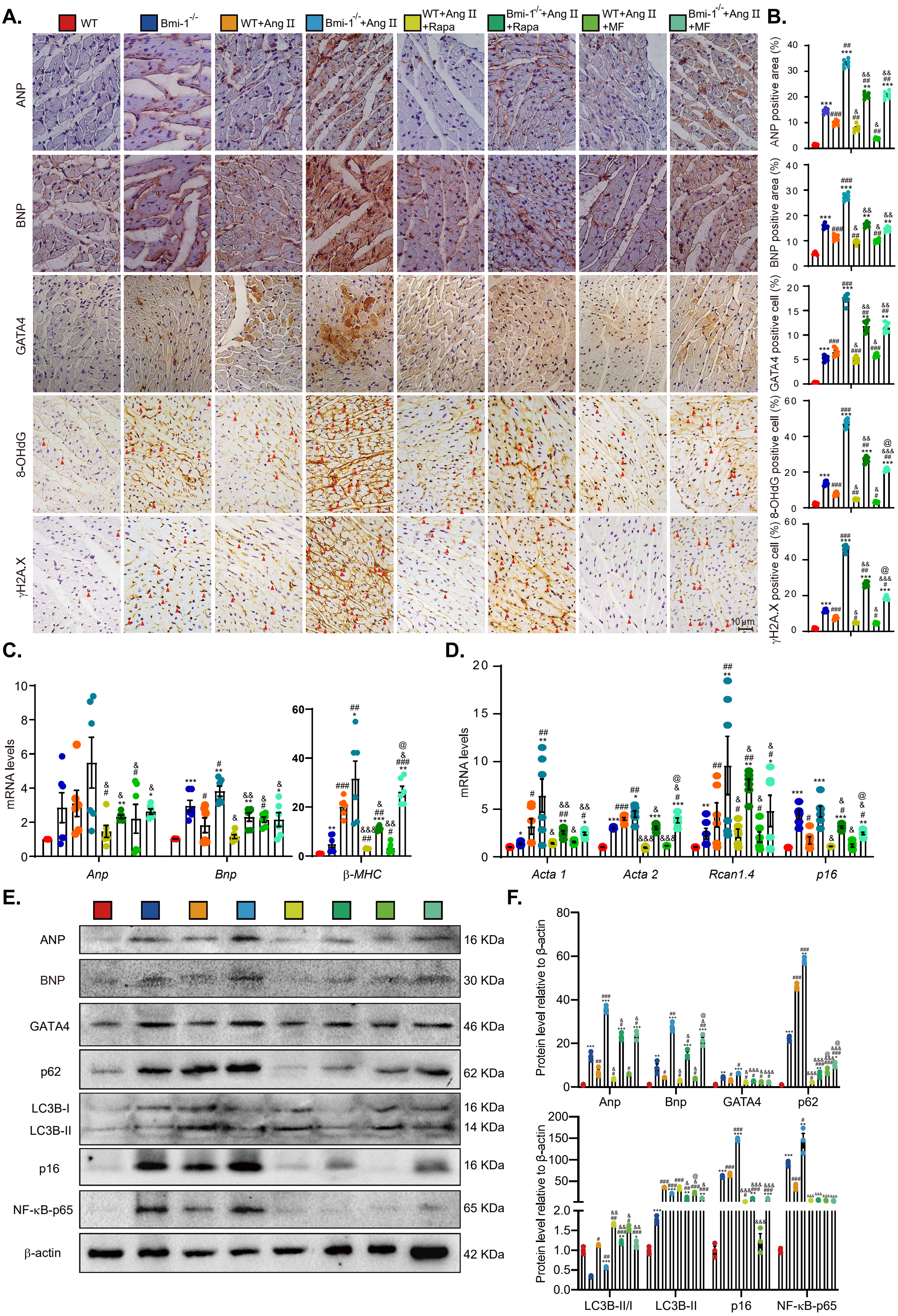
A.
WT
Bmi-1-/-
WT+Ang II
Bmi-1-/-+Ang II
WT+Ang II +Rapa
Bmi-1-/-+Ang II +Rapa
WT+Ang II +MF
Bmi-1-/-+Ang II +MF
ANP
BNP
GATA4
8-OHdG
γH2A.X
10μm
B.
ANP positive area (%)
BNP positive area (%)
GATA4 positive cell (%)
8-OHdG positive cell (%)
γH2A.X positive cell (%)
C.
mRNA levels
Anp
Bnp
β-MHC
D.
mRNA levels
Acta 1
Acta 2
Rcan1.4
p16
E.
ANP 16 KDa
BNP 30 KDa
GATA4 46 KDa
p62 62 KDa
LC3B-I 16 KDa
LC3B-II 14 KDa
p16 16 KDa
NF-κB-p65 65 KDa
β-actin 42 KDa
F.
Protein level relative to β-actin
Anp
Bnp
GATA4
p62
LC3B-II/I
LC3B-II
p16
NF-κB-p65

Figure S4-2

G.

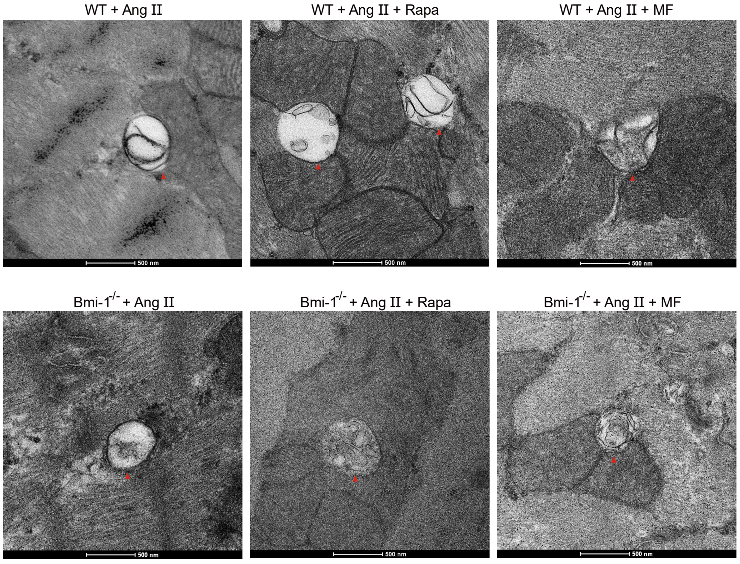
WT + Ang II
WT + Ang II + Rapa
WT + Ang II + MF
$Bmi\text{-}1^{-/-}$ + Ang II
$Bmi\text{-}1^{-/-}$ + Ang II + Rapa
$Bmi\text{-}1^{-/-}$ + Ang II + MF
500 nm

Figure S5

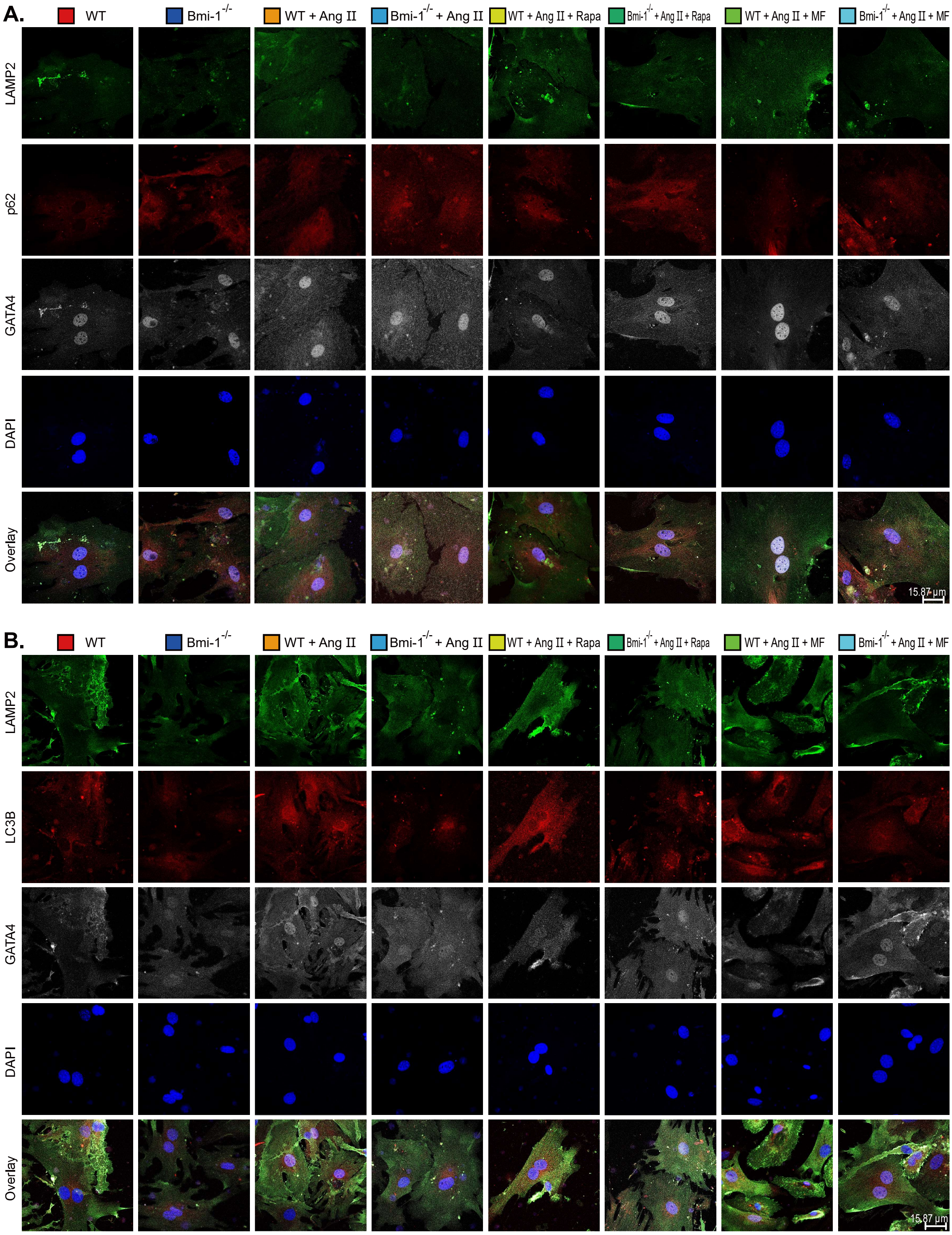
A.
WT
Bmi-1-/-
WT + Ang II
Bmi-1-/- + Ang II
WT + Ang II + Rapa
Bmi-1-/- + Ang II + Rapa
WT + Ang II + MF
Bmi-1-/- + Ang II + MF
LAMP2
p62
GATA4
DAPI
Overlay
15.87 μm
B.
WT
Bmi-1-/-
WT + Ang II
Bmi-1-/- + Ang II
WT + Ang II + Rapa
Bmi-1-/- + Ang II + Rapa
WT + Ang II + MF
Bmi-1-/- + Ang II + MF
LAMP2
LC3B
GATA4
DAPI
Overlay
15.87 μm

Figure S6

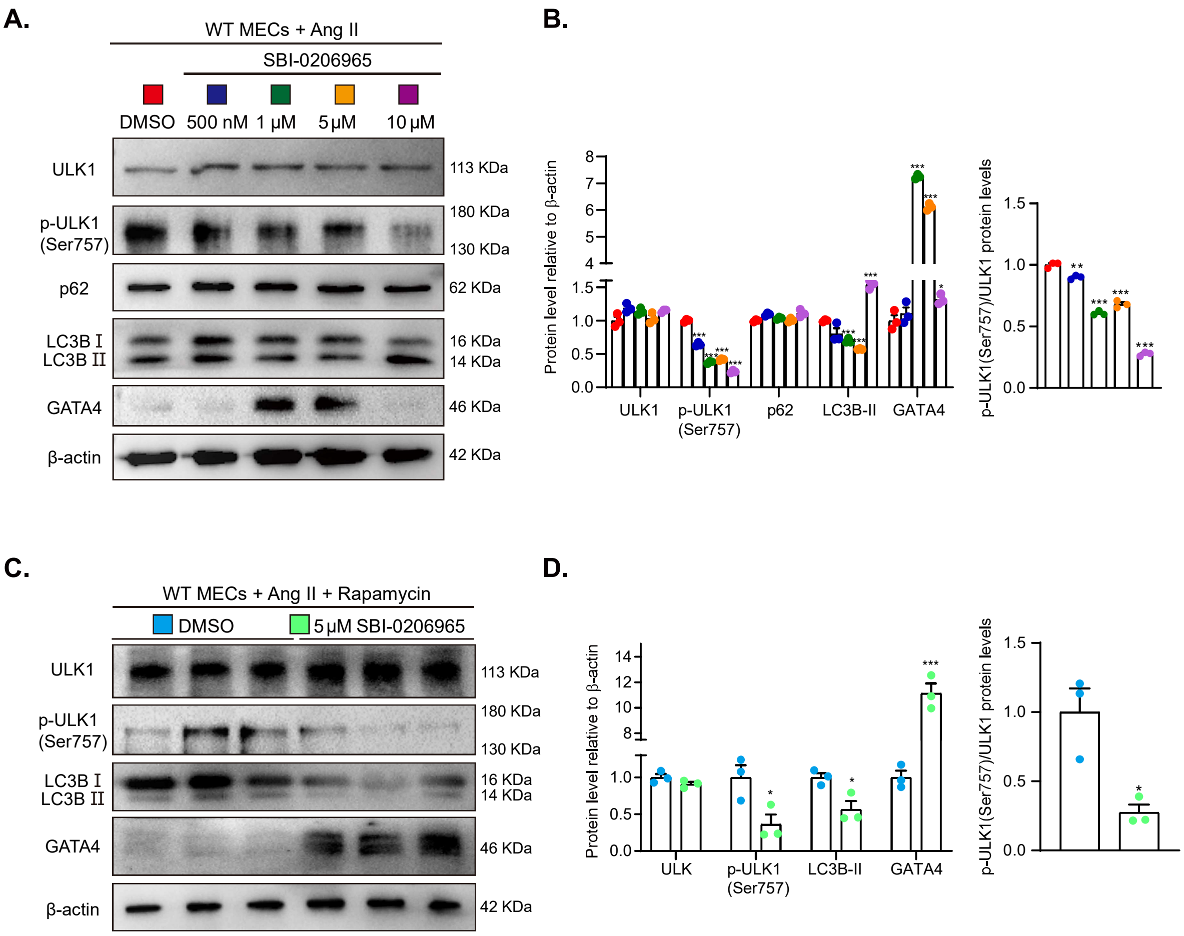
A.
WT MECs + Ang II
SBI-0206965
DMSO
500 nM
1 μM
5 μM
10 μM
ULK1
p-ULK1 (Ser757)
p62
LC3B I
LC3B II
GATA4
β-actin
113 KDa
180 KDa
130 KDa
62 KDa
16 KDa
14 KDa
46 KDa
42 KDa
B.
Protein level relative to β-actin
ULK1
p-ULK1 (Ser757)
p62
LC3B-II
GATA4
p-ULK1(Ser757)/ULK1 protein levels
C.
WT MECs + Ang II + Rapamycin
DMSO
5 μM SBI-0206965
ULK1
p-ULK1 (Ser757)
LC3B I
LC3B II
GATA4
β-actin
113 KDa
180 KDa
130 KDa
16 KDa
14 KDa
46 KDa
42 KDa
D.
Protein level relative to β-actin
ULK
p-ULK1 (Ser757)
LC3B-II
GATA4
p-ULK1(Ser757)/ULK1 protein levels

Figure S7

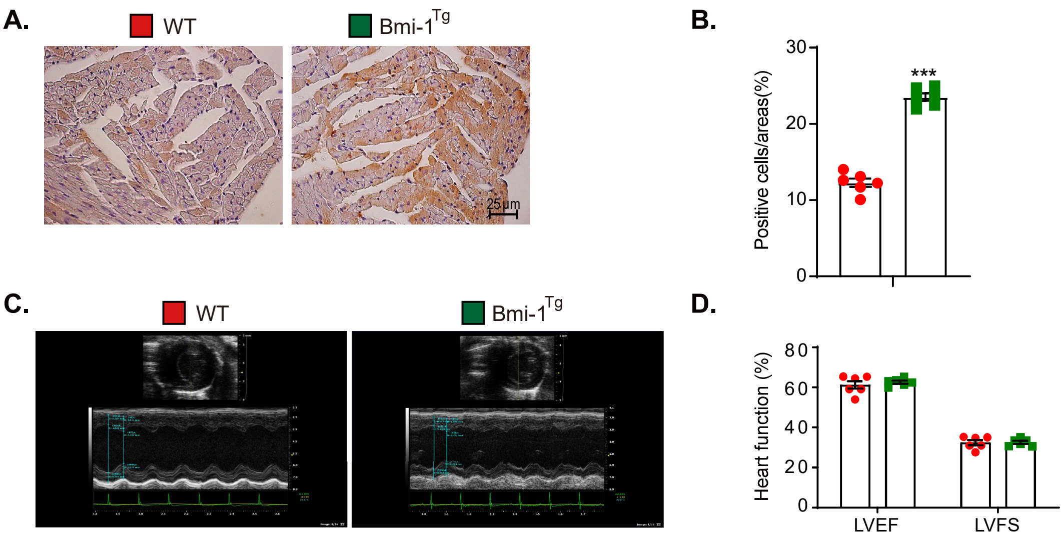

A.
WT
Bmi-1Tg
25 μm
B.
Positive cells/areas(%)
30
20
10
0
***
C.
WT
Bmi-1Tg
D.
Heart function (%)
80
60
40
20
0
LVEF
LVFS

Figure S8

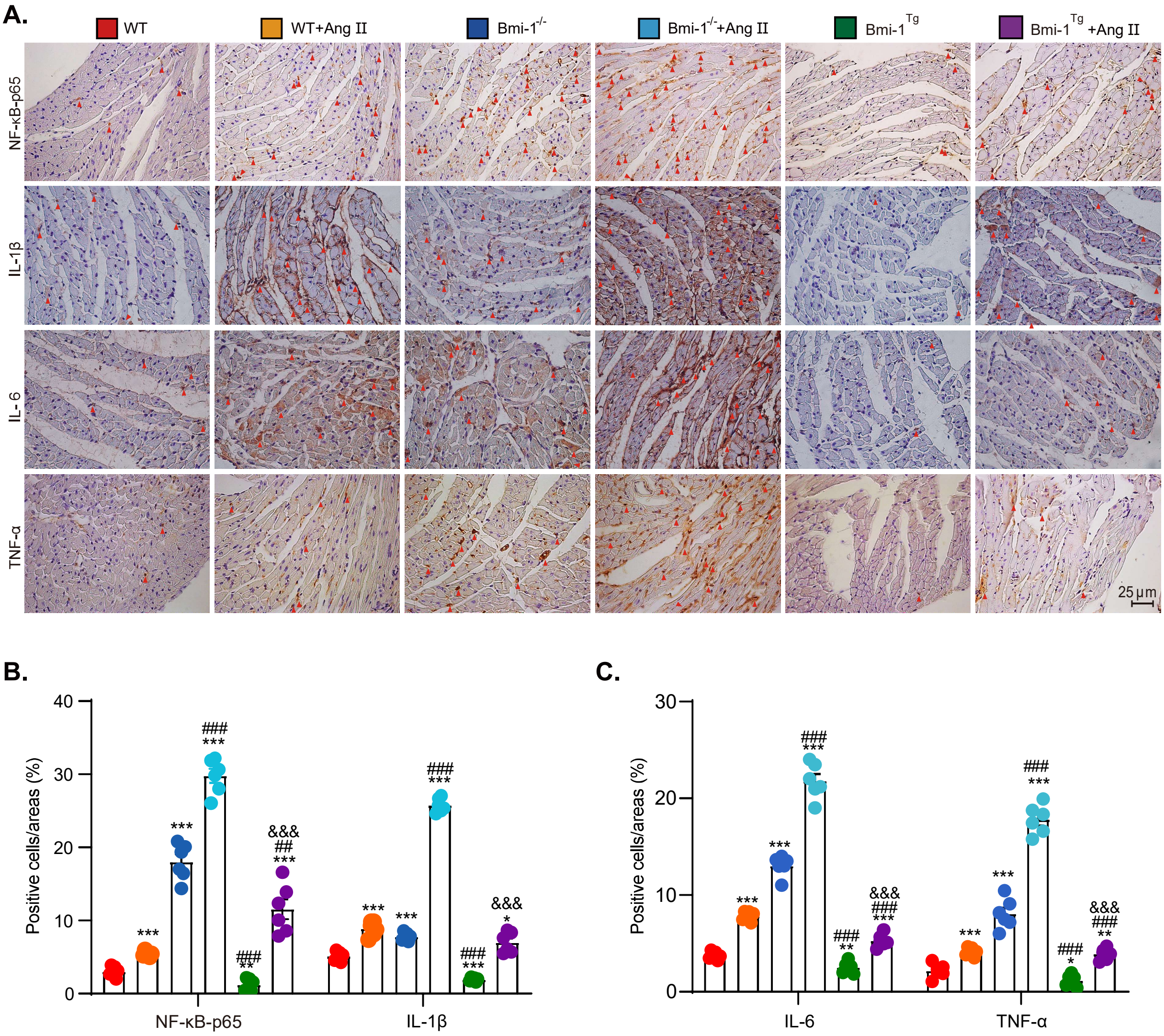
A.
WT
WT+Ang II
Bmi-1-/-
Bmi-1-/-+Ang II
Bmi-1Tg
Bmi-1Tg+Ang II
NF-κB-p65
IL-1β
IL-6
TNF-α
25 μm
B.
Positive cells/areas (%)
NF-κB-p65
IL-1β
C.
Positive cells/areas (%)
IL-6
TNF-α

Figure S9

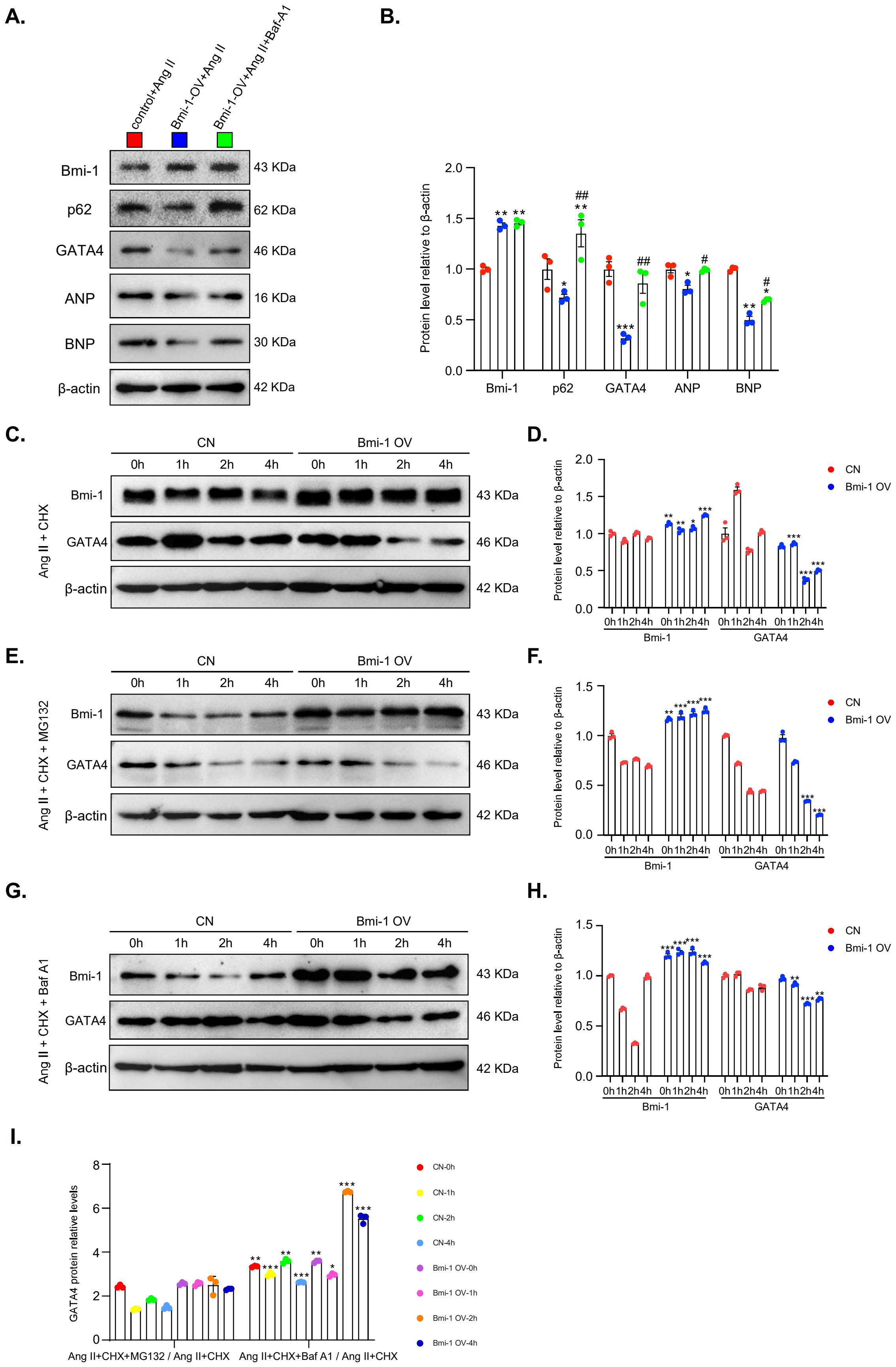

A.
B.
C.
D.
E.
F.
G.
H.
I.
control+Ang II
Bmi-1-OV+Ang II
Bmi-1-OV+Ang II+Baf-A1
Bmi-1
p62
GATA4
ANP
BNP
β-actin
43 KDa
62 KDa
46 KDa
16 KDa
30 KDa
42 KDa
Protein level relative to β-actin
CN
Bmi-1 OV
0h
1h
2h
4h
Ang II + CHX
Ang II + CHX + MG132
Ang II + CHX + Baf A1
GATA4 protein relative levels
CN-0h
CN-1h
CN-2h
CN-4h
Bmi-1 OV-0h
Bmi-1 OV-1h
Bmi-1 OV-2h
Bmi-1 OV-4h
Ang II+CHX+MG132 / Ang II+CHX
Ang II+CHX+Baf A1 / Ang II+CHX

Figure S10

A.

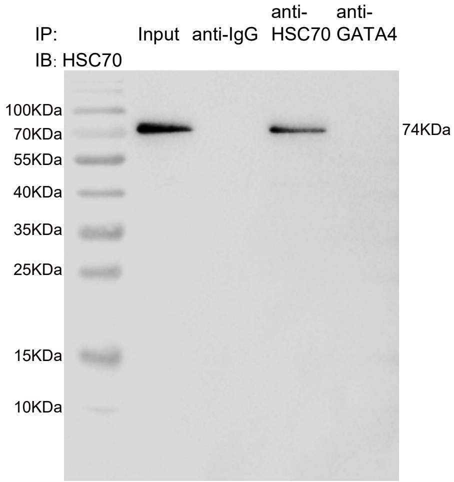

B.

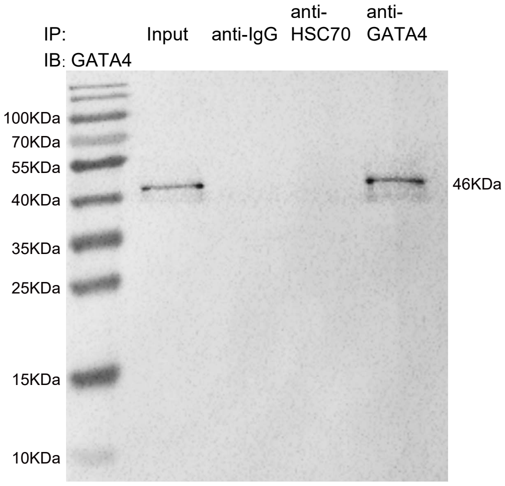

C.

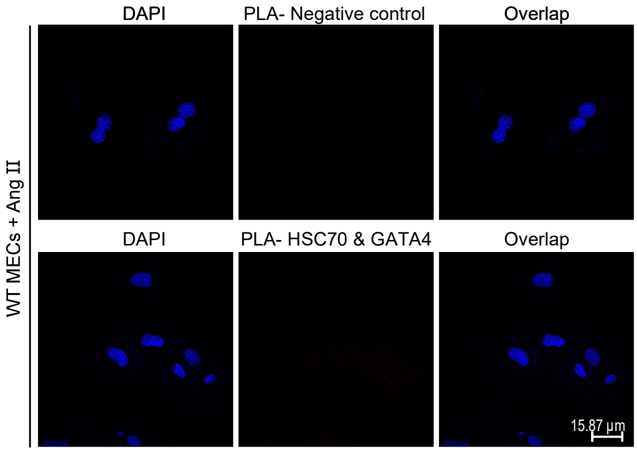

Figure S11

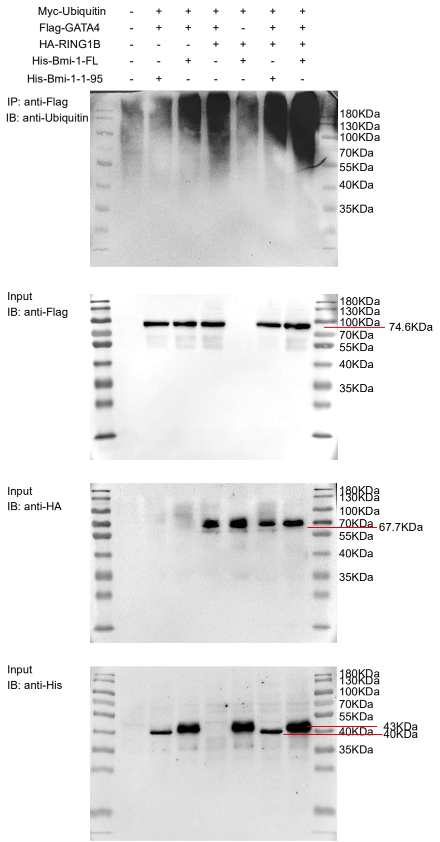

Myc-Ubiquitin - + + + + + +
Flag-GATA4 - + + + - + +
HA-RING1B - - - + + + +
His-Bmi-1-FL - - + - + - +
His-Bmi-1-1-95 - + - - - + -
IP: anti-Flag
IB: anti-Ubiquitin
180KDa
130KDa
100KDa
70KDa
55KDa
40KDa
35KDa
Input
IB: anti-Flag
180KDa
130KDa
100KDa
70KDa
55KDa
40KDa
35KDa
74.6KDa
Input
IB: anti-HA
180KDa
130KDa
100KDa
70KDa
55KDa
40KDa
35KDa
67.7KDa
Input
IB: anti-His
180KDa
130KDa
100KDa
70KDa
55KDa
40KDa
35KDa
43KDa
40KDa

Figure S12

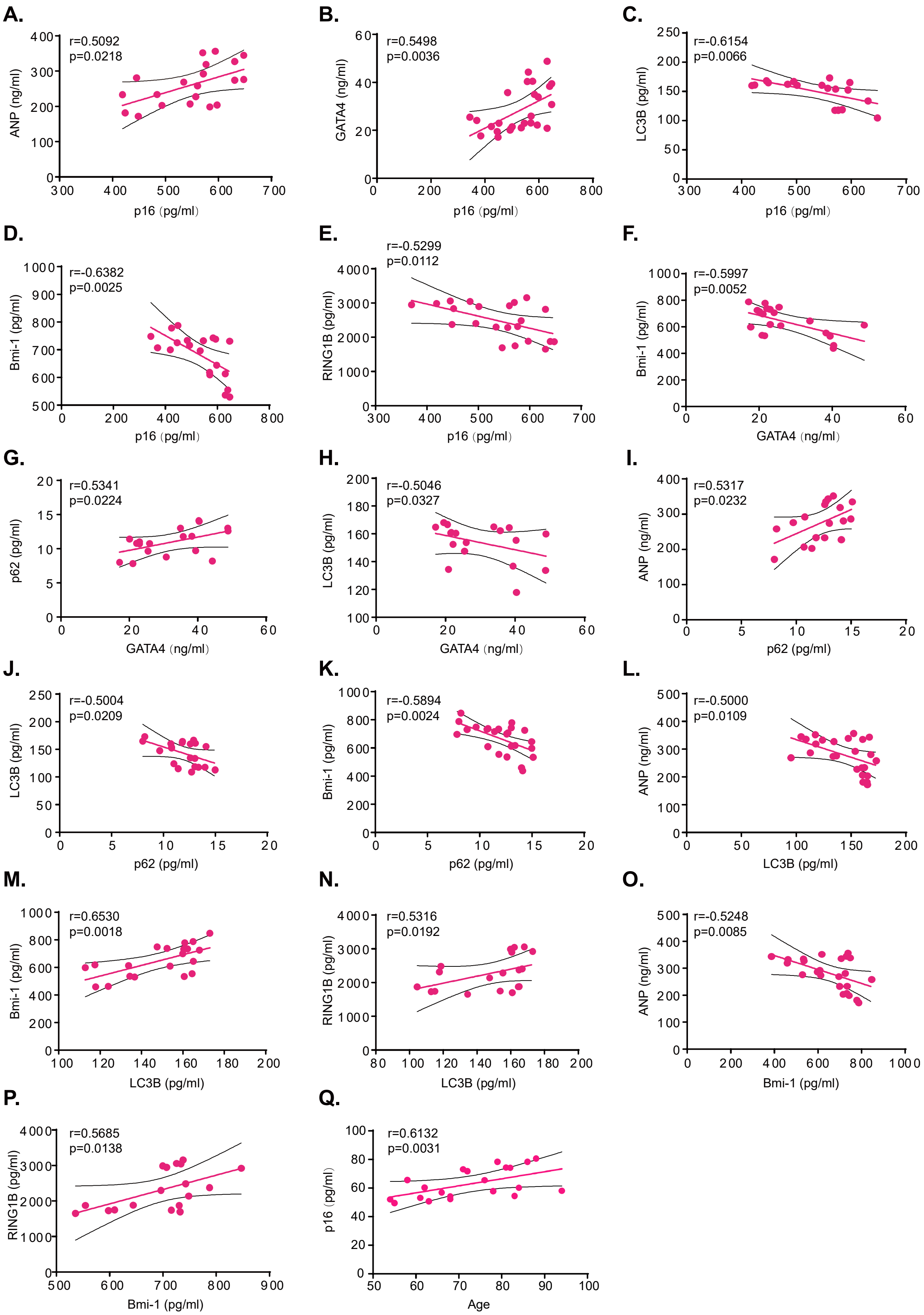

A.
r=0.5092
p=0.0218
ANP (ng/ml)
p16 (pg/ml)
B.
r=0.5498
p=0.0036
GATA4 (ng/ml)
p16 (pg/ml)
C.
r=-0.6154
p=0.0066
LC3B (pg/ml)
p16 (pg/ml)
D.
r=-0.6382
p=0.0025
Bmi-1 (pg/ml)
p16 (pg/ml)
E.
r=-0.5299
p=0.0112
RING1B (pg/ml)
p16 (pg/ml)
F.
r=-0.5997
p=0.0052
Bmi-1 (pg/ml)
GATA4 (ng/ml)
G.
r=0.5341
p=0.0224
p62 (pg/ml)
GATA4 (ng/ml)
H.
r=-0.5046
p=0.0327
LC3B (pg/ml)
GATA4 (ng/ml)
I.
r=0.5317
p=0.0232
ANP (ng/ml)
p62 (pg/ml)
J.
r=-0.5004
p=0.0209
LC3B (pg/ml)
p62 (pg/ml)
K.
r=-0.5894
p=0.0024
Bmi-1 (pg/ml)
p62 (pg/ml)
L.
r=-0.5000
p=0.0109
ANP (ng/ml)
LC3B (pg/ml)
M.
r=0.6530
p=0.0018
Bmi-1 (pg/ml)
LC3B (pg/ml)
N.
r=0.5316
p=0.0192
RING1B (pg/ml)
LC3B (pg/ml)
O.
r=-0.5248
p=0.0085
ANP (ng/ml)
Bmi-1 (pg/ml)
P.
r=0.5685
p=0.0138
RING1B (pg/ml)
Bmi-1 (pg/ml)
Q.
r=0.6132
p=0.0031
p16 (pg/ml)
Age

Figure S13

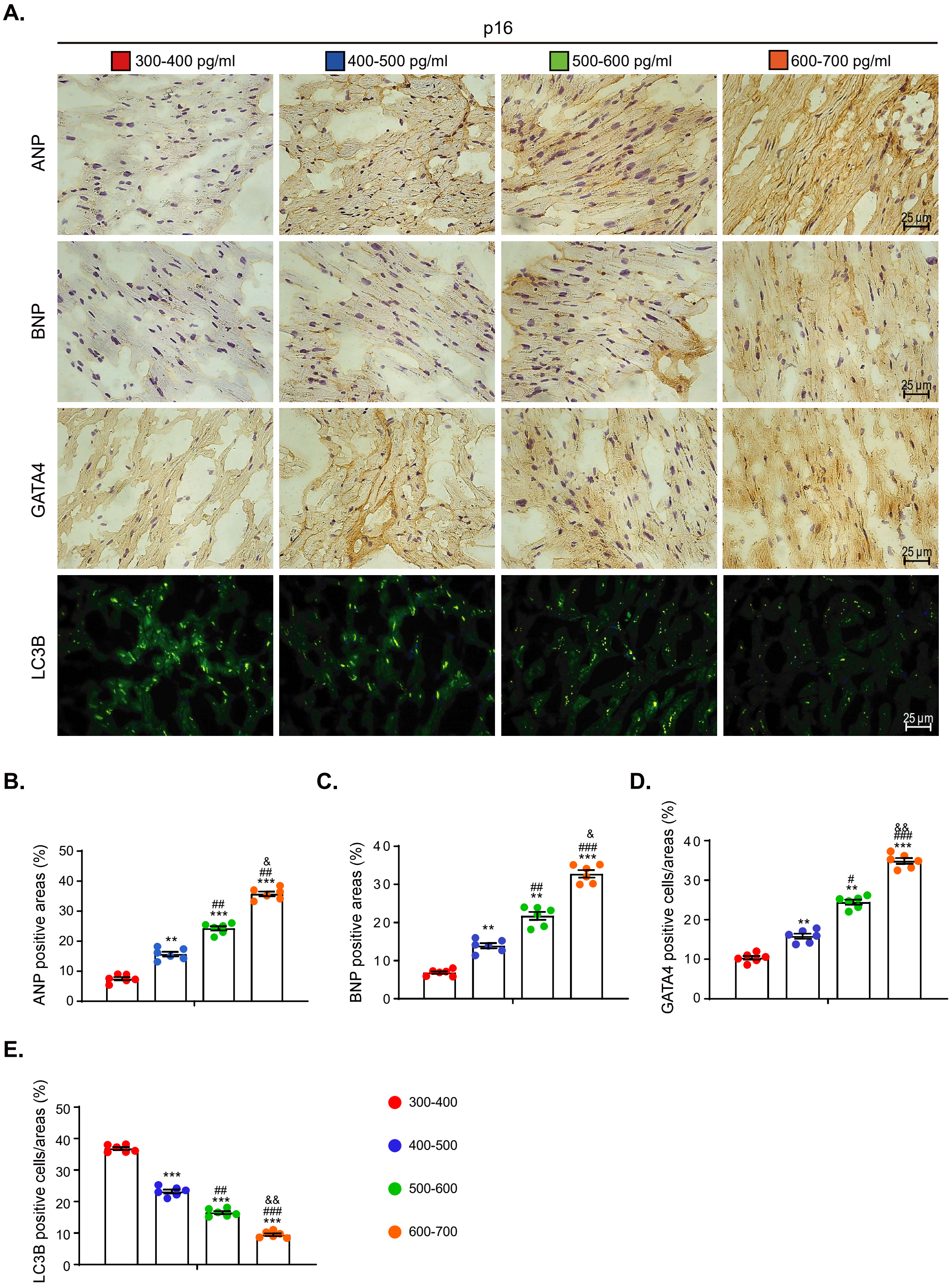

A.
p16
300-400 pg/ml
400-500 pg/ml
500-600 pg/ml
600-700 pg/ml
ANP
BNP
GATA4
LC3B
25 μm
B.
ANP positive areas (%)
C.
BNP positive areas (%)
D.
GATA4 positive cells/areas (%)
E.
LC3B positive cells/areas (%)
300-400
400-500
500-600
600-700
