# Supplementary material for: Bmi‐1‐RING1B prevents GATA4‐dependent senescence‐associated pathological cardiac hypertrophy by promoting autophagic degradation of GATA4
Source: Clin Transl Med. 2022 Apr 7;12(4):e574. doi: 10.1002/ctm2.574 (PMC8989148; doi:10.1002/ctm2.574)
Supplement: Supplementary file 2 — Supplementary Information 2: Figures S1–S13 Legends [file CTM2-12-e574-s003.docx]

**Figures S1-13 Legends**

**Figure S1 Aging induces *p65* (*RelA*) and *TRAF3IP2* upregulation in heart**

The mRNA levels of *p65* (*RelA*) and *TRAF3IP2* were detected in myocardial tissues of young (8-week-old) and aging (20-month-old) mice, and calculated as ratio to *β-actin* mRNA and expressed relative to young mice. Six mice per group were used for experiments. Values are mean ± SEM of six determinations per group, *P <0.05, **P <0.01 compared with the WT young mice; Unpaired t-test for bar graphs.

**Figure S2 *Bmi-1* deficiency induces myocardial dysfunction, RAAS activation and senescence**

(A) Color Doppler echocardiography for three-, six- and nine-week-old mice. (B) Left ventricular ejection fraction (LVEF). (C) Left ventricular shortened fraction (LVFS); values are mean ± SEM of six determinations per group, *P <0.05, **P <0.01, ***P <0.001 compared with the WT mice; ^#^P<0.05 compared with three-week-old isotype mice; ^&&^P <0.01 compared with six-week-old isotype mice. All the following detection were from six-week-old WT and *Bmi-1^-/-^* mice. (D) Ang II and ALD levels were detected in plasma by radioimmunoassay. (E) Western blots of aortic and cardiac extracts showing Renin and Ang II, β-actin was the loading control. (F) Protein levels relative to β-actin were assessed by densitometric analysis. (G) ROS in heart or aorta was detected with DCFDA by flow cytometry. (H) Western blots of cardiac and aortic extracts showing p16, pChk2 (Thr68) and SOD2. β-actin was the loading control. (I) Protein levels relative to β-actin were assessed by densitometric analysis. (J) *P16* mRNA levels in heart and aorta were detected by real-time RT-PCR, calculated as ratio to *β-actin* mRNA and expressed relative to WT. (K) Western blots of cardiac or Aorta extracts showing p19, p21, p53, NF-κB-p65 and p-p65 (Ser536). β-actin was the loading control. (L-M) Protein levels relative to β-actin were assessed by densitometric analysis. Six mice per group were used for experiments. Values are mean ± SEM from six determinations per group, *P <0.05, **P <0.01, ***P <0.001 compared with WT group. One-way ANOVA test (B-D), unpaired Student’s t-test (F, G, I, J, L and M).

**Figure S3 RAAS activation, impaired mitochondrial structure, and cardiac hypertrophy and dysfunction were ameliorated by NAC treatment in *Bmi-1^-/-^* mice**

(A) ROS in hearts from the three groups of six-week-old mice by flow cytometry (AF: Auto Fluorescence). (B) Representative electron micrographs of cardiac sections. Black arrowheads: vacuolized mitochondria (serious swelling); White arrowheads: reduced and disordered myofibrils; Black arrows: mitochondria swelling and cristae vague; White arrows: lipid droplets. (C) Western blots of cardiac extracts showing Renin, Ang II and p16. β-actin was the loading control. (D) Protein levels relative to β-actin were assessed by densitometric analysis. (E) Color Doppler echocardiography for six-week-old mice. (F) Left ventricular ejection fraction (LVEF). (G) Left ventricular shortened fraction (LVFS). (H) Representative micrographs of paraffin-embedded heart ventricular wall sections for wheat germ agglutinin (WGA) staining. (I) Myocyte cross-sectional area is relative to WT mice. Six mice per group were used for experiments. Values are mean ± SEM from six determinations per group, *P <0.05, ** P <0.01, *** P <0.001 compared with WT group; ^#^P <0.05, ^###^P <0.001 compared with *Bmi-1^-/-^* group. One-way ANOVA test (D, F, G and I).

**Figure S4 Autophagy agonist supplementation improves GATA4-dependent SA-PCH in WT mice more than *Bmi-1*^-/-^ mice**

Autophagy agonist Metformin (as a water supplement, 1 mg/ml) or Rapamycin (intraperitoneal injection, 2 mg/kg/day) was administered to four-week-old WT and *Bmi-1^-/-^* mice that were treated with Ang II (1.3 mg/kg/day) for 4 weeks. (A) Representative micrographs of paraffin-embedded heart sections stained immunohistochemically for ANP, BNP, GATA4, 8-OHdG and γH2A.X (indicated by red arrowhead). (B) The percentage of cells positive for ANP, BNP, GATA4, 8-OHdG and γH2A.X or positive area relative to total cells or area. (C) *Anp*, *Bnp* and *β-MHC* mRNA levels in hearts by real-time RT-PCR, calculated as a ratio to *β-actin* mRNA and expressed relative to WT. (D) *Acta 1*, *Acta 2*, *Rcan1.4*, and *p16* mRNA levels in hearts by real-time RT-PCR, calculated as a ratio to *β-actin* mRNA and expressed relative to WT. (E) Western blots of cardiac tissue extracts showing ANP, BNP, GATA4, LC3B, p62, p16 and NF-κB-p65, β-actin was the loading control. (F) Protein levels relative to β-actin were assessed by densitometric analysis. The experiments were performed with six mice per group. Statistical analysis was performed with One-way ANOVA test, values are mean ± SEM from six determinations per group, *P <0.05, **P <0.01, ***P <0.001 compared to WT group with the same treatment; ^#^P <0.05, ^##^P <0.01, ^###^P <0.001 compared to control group with the same genotype; ^&^P <0.05, ^&&^P <0.01, ^&&&^P <0.001 compared to Ang II-treated group with the same genotype; ^@^P <0.05 compared to Ang II & Rapa-treated group with the same genotype. (G) Representative electron micrographs of cardiac sections. Red arrowheads: autophagic vacuoles.

**Figure S5 Autophagy agonist supplementation increases autolysosomes in WT mice more than *Bmi-1*^-/-^ mice**

Mouse embryonic cardiomyocytes (MECs) were isolated and cultured from 13.5-day fetal hearts, and were treated with Ang II (5 × 10^-6^ mol/L for 70h) to induce hypertrophy and were rescued with metformin (MF) (2.5 mM for 70h) or rapamycin (Rapa) (100 nM for 70h). (A-B) Representative micrographs of MECs for Lamp2, p62 and GATA4, or Lamp2, LC3B and GATA4.

**Figure S6 SBI-0206965 inhibited p62 mediated autophagic degradation of GATA4 induced by inhibition of mTOR**

Mouse embryonic cardiomyocytes (MECs) from WT mice were isolated and cultured from 13.5-day fetal hearts, and were treated with Ang II (5 × 10^-6^ mol/L for 70h) to induce hypertrophy and/or treated with rapamycin (100 nM for 70h). Cells were treated with ULK1 inhibitor SBI-0206965 at 500 nM, 1 µM, 5 µM or 10 µM for 70 h. (A) Western blot was used to detected ULK1, p-ULK1 (Ser757), p62, LC3B, GATA4 and p-ULK1 (Ser757)/ ULK1, β-actin was the loading control. (B) Protein levels relative to β-actin were assessed by densitometric analysis. (C) Western blot was used to detected ULK1, p-ULK1 (Ser757), LC3B, GATA4 and p-ULK1 (Ser757)/ ULK1, and β-actin was the loading control. (D) Protein levels relative to β-actin were assessed by densitometric analysis. Values are mean ± SEM of three determinations per group, *P <0.05, **P <0.01, ***P <0.001 compared with the DMSO group; unpaired Student’s t-test.

**Figure S7 *Bmi-1^Tg^* mice had increased *Bmi-1* protein in heart tissues and developed normally without structural or functional defects**

All the following detection were from seven-week-old WT and *Bmi-1^Tg^* mice (A) Representative micrographs of paraffin-embedded heart sections immunohistochemical staining for Bmi-1. (B) The percentage of Bmi-1 positive cells or areas relative to total cells or areas. (C) Color Doppler echocardiography for seven-week-old WT and *Bmi-1^Tg^* mice. (D) Left ventricular ejection fraction (LVEF) and left ventricular shortened fraction (LVFS). Statistical analysis was performed with unpaired Student’s t-test, values are mean ± SEM from six determinations per group, ***P <0.001 compared to WT group.

**Figure S8 *Bmi-1* overexpression in myocardial tissue represses** **NF-κB-p65-dependent SASP in SA-PCH**

Four-week-old WT, *Bmi-1^-/-^* and *Bmi-1^Tg^* mice were treated with Ang II (1.3 mg/kg/day) for 4 weeks. (A) Representative micrographs of paraffin-embedded heart sections immunohistochemical staining for NF-κB-p65, IL-1β, IL-6 and TNF-α (indicated by red arrowhead). (B) The percentage of cells positive for NF-κB-p65, IL-1β, IL-6 and TNF-α positive cells or areas relative to total cells or area. The experiments were performed with six mice per group. Statistical analysis was performed with One-way ANOVA test, values are mean ± SEM from six determinations per group, *P <0.05, ** P <0.01, *** P <0.001 compared with WT group; ^##^P <0.01, ^###^P <0.001 compared with *Bmi-1^-/-^* group; ^&&&^P <0.001 compared with *Bmi-1^Tg^* group.

**Figure S9 GATA4 degraded by Bmi-1 was mainly dependent on autophagy rather than proteasome**

The lentivirus with *Bmi-1* overexpression (OV) was constructed and transfected to human myocardial AC16 cells. AC16 cells were treated with Ang II (1 × 10^-5^ mol/L for 70 h) to induce hypertrophy and/or treated with Bafilomycin A1 (Baf-A1) (100 nM for 12h). (A) Western blots of cell extracts showing Bmi-1, p62, GATA4, ANP and BNP, β-actin was the loading control. (B) Protein levels relative to β-actin were assessed by densitometric analysis. Statistical analysis was performed with One-way ANOVA test. Values are mean ± SEM of three determinations per group, *P <0.05, **P <0.01, ***P <0.001 compared with control + Ang II group; ^#^P <0.05, ^##^P <0.01 compared with Ang II-treated Bmi-1-OV group. (C) AC16 cells were treated with Ang II (1 × 10^-5^ mol/L for 70h) and Cycloheximide (CHX) (100 μM for 0, 1, 2 or 4h), and Western blot was performed to detect the protein levels including Bmi-1 and GATA4, β-actin was the loading control. (D) Protein levels relative to β-actin were assessed by densitometric analysis. (E) AC16 cells were treated with Ang II (1 × 10^-5^ mol/L for 70h) and MG132 (5 μM for 12h) and CHX (100 μM for 0, 1, 2 or 4h), and Western blot was performed to detect the protein levels including Bmi-1 and GATA4, β-actin was the loading control. (F) Protein levels relative to β-actin were assessed by densitometric analysis. (G) AC16 cells were treated with Ang II (1 × 10^-5^ mol/L for 70h) and Baf-A1 (100 nM for 12h) and CHX (100 μM for 0, 1, 2 or 4h), and Western blot was performed to detect the protein levels including Bmi-1 and GATA4, β-actin was the loading control. (H) Protein levels relative to β-actin were assessed by densitometric analysis. Statistical analysis was performed with unpaired Student’s t-test, values are mean ± SEM from three determinations per group, *P <0.05, **P <0.01, ***P <0.001 compared with the CN group at the same time point. (I) GATA4 protein relative levels, the Ang II- and CHX-treated group was the control. Statistical analysis was performed with unpaired Student’s t-test, values are mean ± SEM from three determinations per group, *P <0.05, **P <0.01, ***P <0.001 compared with the ratio of GATA4 relative level in the Ang II- and CHX- and MG132-treated group relative to the Ang II- and CHX-treated group at the same time point.

**Figure S10 P62 mediated lysosomal degradation of GATA4 was not HSC70-mediated CMA**

Mouse embryonic cardiomyocytes (MECs) from WT mice were isolated and cultured from 13.5-day fetal hearts, and treated with Ang II (5 × 10^-6^ mol/L for 70h) to induce hypertrophy. (A-B) Proteins extracted from cells were used for anti-HSC70 and anti-GATA4 immunoprecipitation. Western blots were used for detecting HSC70 and GATA4. (C) Representative micrographs of Duolink Proximity Ligation Assay (PLA) for interaction between HSC70 and GATA4, with DAPI for nuclei.

**Figure S11** **Effect of full length (FL) of Bmi-1 in ubiquitination of GATA4 superior to residues 1-95**

The overexpression plasmids of Myc-Ubiquitin, Flag-GATA4, HA-RING1B, and His-Bmi-1-FL or His-Bmi-1-1-95 residues were transfected into 293T cells. The level of ubiquitination of GATA4 and fusion proteins were detected. Cell proteins were extracted for anti-Flag-tag immunoprecipitation and were detected anti-Ubiquitin with Western blots. The input proteins were detected for anti-Flag-tag, -HA-tag or -His-tag with Western blots.

**Figure S12 Bmi-1-RING1B prevented GATA4-dependent SA-PCH in human** **myocardium by promoting autophagic degradation**

Human myocardial tissues were examined for Bmi-1, RING1B, p16, GATA4, ANP, p62 and LC3B protein levels by ELISA and analyzed for correlations. (A) ANP and p16; (B) GATA4 and p16; (C) LC3B and p16; (D) Bmi-1 and p16; (E) RING1B and p16; (F) Bmi-1 and GATA4; (G) p62 and GATA4; (H) LC3B and GATA4; (I) ANP and p62; (J) LC3B and p62; (K) Bmi-1 and p62; (L) ANP and LC-3B; (M) Bmi-1 and LC-3B; (N) RING1B and LC3B; (O) ANP and Bmi-1; (P) RING1B and Bmi-1; (Q) P16 and age. Gaussian distributed data were analyzed by Pearson’s r and non-Gaussian distributed data were analyzed by Spearman’s r. P-values were two-sided and values less than 0.05 was considered statistically significant.

**Figure S13 Bmi-1-RING1B prevented GATA4-dependent SA-PCH in human** **myocardium by promoting autophagic degradation**

Human myocardial tissues examined for p16 protein levels by ELISA. (A) The frozen tissue sections of human myocardial tissues were detected the ANP, BNP, GATA4 and LC3B immunohistological expression according to the p16 protein levels. (B-E) Percentage of cells or areas positive for ANP, BNP, GATA4 and LC3B. Statistical analysis was performed with One-way ANOVA test, values are mean ± SEM from 4 determinations per group. **P < 0.01, ***P < 0.001 compared with 300-400 pg/ml group; ^#^P < 0.05, ^##^P < 0.01, ^###^ P < 0.001 compared with 400-500 pg/ml group; ^&^P < 0.05, ^&&^P < 0.01 compared with 500-600 pg/ml group.
