# Supplementary material for: Bmi‐1‐RING1B prevents GATA4‐dependent senescence‐associated pathological cardiac hypertrophy by promoting autophagic degradation of GATA4
Source: Clin Transl Med. 2022 Apr 7;12(4):e574. doi: 10.1002/ctm2.574 (PMC8989148; doi:10.1002/ctm2.574)
Supplement: Supplementary file 3 — Supplementary Information 4: Complete Materials and Methods [file CTM2-12-e574-s001.docx]

**Complete Materials and Methods**

**High throughput sequencing analysis**

The gene expression profile data comparing heart tissue from young and aging mice were downloaded from Gene Expression Omnibus (GEO, https://www.ncbi.nlm.nih.gov/geo/) database. GSE161078 contains the mRNA expression data of whole hearts from 14-week-old, 12-month-old and 18-month-old mice. Before analyzing differentially expressed genes (DEGs) using R (version 4.0.3), unrelated samples were excluded. The correlation between genes was calculated by Pearson correlation analysis. Following data reprocessing and the identification of DEGs, Gene Ontology (GO) analysis and Kyoto Encyclopedia of Genes and Genomes (KEGG) analyses were performed. The correlation coefficients between each gene and *Bmi-1* were calculated for further gene set enrichment analysis (GSEA).

**Mice and genotyping**

Two mouse models were used in this study: The *Bmi-1* heterozygous (*Bmi-1*^+/−^) mouse line with a C57BL/6J background was from McGill University[^1^](#_ENREF_1). The *Bmi-1* allele was detected using the *Bmi-1* forward primer 5′-CAGTTAGGCAGTATGTAGTTTTC-3′ and *Bmi-1* reverse primer 5′-GTTGTGGTGGAGTGTAAGAGTGT-3′. The null *Bmi-1* allele was detected using the Neo forward primer 5′-AAGATGTTGGCGACCTCGTATTGG-3′ and the Neo reverse primer 5′-GCAAGACCTGCCTGAAACCGAACT-3′. *Bmi-1* overexpression in mice with a C57BL/6J background under the control of the 2.4-kb *Prx1* promoter (*Bmi1^Tg^*) was generated at Nanjing Medical University (Nanjing, China). Previous report demonstrated that Paired-related homeobox transcription factors PRX1 was verified to play essential roles in limb, heart, and craniofacial development by analyses of knockout animals[^2^](#_ENREF_2). In previous report from our lab, *Bmi-1* overexpression in mice with a C57BL/6J background under the control of the 2,476 bp *Prx1* promoter (*Bmi-1^Tg^*) carrying restriction site was generated. Two founder lines (7#, 8#) have been generated and characterized[^3^](#_ENREF_3). The 1,001 bp fragment corresponding to the *Bmi-1* transcript was amplified[^3^](#_ENREF_3). The *Bmi-1^Tg^* mice used in the experiments had single copy gene in the genome, which were produced by backcrossing *Bmi-1^Tg^* mice with WT mice. Genotype was determined using forward primer 5'-AATGCTGGAGAGCTGGAAAG-3' and reverse primer 5'-GTCAAGAAGACAGGGCCAGG-3' as previously described[^4^](#_ENREF_4)^,^ [^5^](#_ENREF_5). Mice were housed in the SPF Laboratory Animal Center of Nanjing Medical University. All experiments were conducted in accordance with the guidelines for the Care and Use of Laboratory Animals published by the US National Institutes of Health (NIH Publication, 8th Edition, 2011) and uses of mice were approved by the Institutional Animal Care and Use Committee of Nanjing Medical University (Permit Number: IACUC-1706001).

For chronic induction of cardiac hypertrophy, eight-week-old male mice were anesthetized with 0.02 g/ml pentobarbital sodium (50 mg/kg, intraperitoneal injection) and implanted with ALZET® Micro-Osmotic Pumps (Model 1004) (#10370-16, DURECT Corporation, CA, USA) in the subcutaneous skin on the back for infusing angiotensin II (Ang II, #A9525, Sigma-Aldrich, St. Louis, MO, USA) at a dose of 1.3 mg/kg/day for 4 weeks. For tissue collection, mice were anaesthetized using overdose anesthesia (50 mg/kg body weight, intraperitoneal injection) and then sacrificed by cervical dislocation and hearts were used for *ex vivo* experiments.

**Samples of human myocardium tissues**

Human myocardium samples were obtained from 25 autopsied donors at the Department of Human Anatomy at Nanjing Medical University. Anatomical methods and all experimental protocols^[6](#_ENREF_6)^ were approved by the Committee on the Ethics of Nanjing Medical University (Permit Number: 2019-902). Donors were 39–94 years old and had no tumors, acquired immune deficiency syndrome, autoimmune disease, respiratory chronic infections or inflammatory disease before they died[^6^](#_ENREF_6).

**Mouse embryonic cardiomyocytes (MECs) cultures**

Female mice that were 13.5 days pregnant were anesthetized with 3% pentobarbital sodium (40 mg/kg), and fetal mouse heart tissues were separated with blunt dissection aseptically and rinsed three times in PBS (0.01 mM PO_4_^3-^, pH 7.4) containing 200 U/ml penicillin and 200 μg/ml streptomycin (Gibco, Grand Island, NY, USA). Hearts were minced and digested for 30 min in digestive solution with 1 mg/ml collagenase D (0.30 U/mg lyophilisate, Roche Diagnostics GmbH, Mannheim, BW, Germany) and 2% fetal bovine serum (FBS) (v/v) (Gibco) in normal culture medium Dulbecco's Modified Eagle Medium (DMEM)/F12 (Gibco) on a constant temperature shaker at 37°C at 175 rpm. Samples were centrifuged at 1500 rpm for 5 min [^6^](#_ENREF_6)^,^ [^7^](#_ENREF_7). Supernatants were discarded and pellets were washed with PBS three times and centrifuged. Pellets of cardiomyocytes were resuspended in 2 ml DMEM/F12 containing 10% (v/v) FBS, 100 U/ml penicillin and 100 μg/ml streptomycin and placed in six-well plates and kept in a humidified 5% CO_2_ incubator at 37°C. Primary cells were observed beating rhythmically at the bottoms of dishes. Cells were harvested after 4–6 days. Primary or second-passage MECs were used.

**Cell line from adult human ventricular cardiomyocytes**

Human AC16 cells (BeNa Culture Collection, Beijing, China) were cultured in DMEM/F12 supplemented with 100 U/ml penicillin, 100 µg/ml streptomycin and 10% FBS (Gibco) until 80% confluent as previously described[^8^](#_ENREF_8)^,^ [^9^](#_ENREF_9). To study the effect of *Bmi-1* knockdown or overexpression on Ang II-induced hypertrophy of human cardiomyocytes, a lentivirus for *Bmi-1* knockdown or overexpression was constructed and transfected into human myocardial AC16 cells. To determine the combined domains of Bmi-1, RING1B, and GATA4, plasmids including truncated and full-length fragments were also transfected into AC16 cells. Previous observations demonstrated that AC16 cells expressed the heart-specific transcription factors GATA4, MYCD and NFATc4, contractile proteins α- and β-MHC, α-cardiac actin, peptide hormones, ANP, BNP and the L-type Ca channel, CACN α1C, and were useful *in vitro* models to study developmental regulation of cardiomyocytes in normal and pathological processes especially for cardiac hypertrophy and heart failure[^9-12^](#_ENREF_9).

**Administration of drugs or reagents**

***Ang II treatment in vivo or in vitro***

For *in vivo* experiments, cardiac hypertrophy mouse models were induced by chronic subcutaneous infusion of Ang II (#A9525, Sigma-Aldrich, USA) in three-week-old mice at 1.3 mg/kg/day using subcutaneous implantable ALZET Micro-Osmotic Pumps (Model 1004) (#10370-16, DURECT Corporation, CA, USA) for four weeks[^13^](#_ENREF_13). Vehicle mice were infused with saline for four weeks. For *in vitro* experiments, MECs or AC16 cells were treated with Ang II at 5 × 10^-6^ mol/L or 1 × 10^-5^ for 70 h[^13^](#_ENREF_13)^,^ [^14^](#_ENREF_14).

***Metformin and rapamycin treatment in vivo or in vitro***

For *in vivo* administration, mice were treated with metformin (#PHR1084, Sigma-Aldrich, USA) in drinking water (1 mg/ml) for four weeks[^15^](#_ENREF_15) and with rapamycin (#553210, Sigma-Aldrich, USA) by intraperitoneal injection (2 mg/kg/day)[^16^](#_ENREF_16) for two weeks. For *in vitro* treatment, cells were treated with metformin at 2.5 mM[^17^](#_ENREF_17) for 70 h or with rapamycin at 100 nM^[18](#_ENREF_18)^ for 70 h.

***ULK1 inhibitor SBI-0206965 treatment in vitro***

For *in vitro* treatment, cells were treated with SBI-0206965^[19](#_ENREF_19)^ (S7885, Selleck Chemicals, TX, USA) at 500 nM, 1 µM, 5 µM or 10 µM for 70 h.

***N-acetylcysteine***

Three-week-old *Bmi-1^-/-^* mice were randomized to water containing N-acetylcysteine (NAC) at 1 mg/ml or ordinary drinking water as previously described[^6^](#_ENREF_6)^,^ [^20^](#_ENREF_20)^,^ [^21^](#_ENREF_21).

**Color Doppler echocardiography**

Mice were depilated at the chest and abdomen and anesthetized with 3% sodium pentobarbital at 40 mg/kg. A high-frequency ultrasound imaging system (Vevo 2100, Visual Sonics, Toronto, Canada) was used to measure left ventricular end-diastolic diameter (LVEDD), left ventricular end-systolic diameter (LVESD), left ventricular end-diastolic volume (LVEDV), and left ventricular end-systolic volume (LVESV), to obtain left ventricular shortened fraction (LVFS) = (LVEDD-LVESD)100%/LVEDD, and left ventricular ejection fraction (LVEF) = (LVEDV-LVESV) 100% / LVEDV[^22^](#_ENREF_22).

**Plasma Ang II and Aldosterone**

Plasma Ang II and aldosterone (ALD) concentrations from six-week-old mice were determined using commercial radioimmunoassay kits according to the manufacturer’s instructions (Beijing North Institute of Biological Technology, Beijing, China) as previously described[^23^](#_ENREF_23).

**Western blots**

Total protein of tissue/cell samples was extracted, and immunoblotting was performed as previously described[^24^](#_ENREF_24). Primary antibodies were against p16 (ab211542, Abcam, Cambridge, MA, USA), renin (#5250, Cell Signaling Technology, Beverly, MA, USA), Ang II (#NBP1-31127, Novus Biologicals, CO, USA), ANP (#AB5490, Millipore, MA, USA; sc-515701, Santa Cruz Biotechnology Inc., Dallas, TX, USA), BNP (#DF6902, Affinity Biosciences, OH, USA; ab19645, Abcam, USA), GATA4 (#19530, Proteintech, IL, USA; sc-25310, Santa Cruz Biotechnology Inc., USA), LC3B (#NB600-1384, Novus Biologicals), p62 (#39749, Cell Signaling Technology, USA), Bmi-1 (#5856, Cell Signaling Technology, USA; 66161-1-Ig, Proteintech, USA), RING1B (#5694, Cell Signaling Technology, USA) and NF-κB-p65 (sc-8008, Santa Cruz Biotechnology Inc., USA; #8242, Cell Signaling Technology, USA), p19 (NB200-106, Novus Biologicals, USA), p21 (#2947, Cell Signaling Technology, USA), p53 (#2424, Cell Signaling Technology, USA), p-p65 (Ser536) (ab76302, Abcam, USA), IκB-α (AF1282, Beyotime Biotechnology, Shanghai, China), p-IκB-α (Ser32) (sc-8404, Santa Cruz Biotechnology Inc., USA), p-Chk2 (Thr68) (PA5-104715, Invitrogen Inc. CA, USA), SOD-2 (NB100-1992, Novus Biologicals, USA), HSC70 (10654-1-AP, Proteintech, USA), p-ULK1 (Ser757) (#14202, Cell Signaling Technology, USA) and ULK1 (sc-390904, Santa Cruz Biotechnology, USA). β-actin (AP0060, Bioworld Technology Inc., MN, USA) was control for total protein.

**RNA extraction and real-time RT-PCR**

Total RNA was extracted from hearts or cells using TRIzol reagent (#15596, Invitrogen Inc., USA) and reverse transcribed with PrimeScript RT Master Mix (Perfect Real Time, Takara Bio Inc., Japan) following the manufacturers’ protocols. Quantitative real-time RT-PCR amplifications were performed as described[^25^](#_ENREF_25). Primers are listed in Table S1 in Supplemental Information (SI) 5.

**Histology**

Heart samples were fixed in periodate-lysine-paraformaldehyde (PLP) solution overnight at 4°C[^23^](#_ENREF_23)^,^ [^26^](#_ENREF_26), embedded in paraffin, and cut into 5-μm samples along the coronal plane using a rotary microtome (Leica Biosystems Nussloch GmbH, Nussloch, Germany). Sections were hydrated in a series of descent graded ethanol solutions and ddH_2_O for the staining by hematoxylin and eosin (H&E), immunohistochemistry, Masson's trichrome (Masson) and wheat germ agglutinin (WGA) after paraffin embedding.

***Immunohistochemical staining***

Staining was as previously described[^7^](#_ENREF_7)^,^ [^23^](#_ENREF_23)^,^ [^26^](#_ENREF_26). Primary antibodies were against ANP (#AB5490, Millipore, MA, USA; sc-515701, Santa Cruz Biotechnology Inc., USA), BNP (#DF6902, Affinity Biosciences, OH, USA; ab19645, Abcam, USA), GATA4 (#19530, Proteintech, USA), 8-OHdG (ab62623, Abcam, USA), γH2A.X (#9718, Cell Signaling Technology, USA), NF-κB-p65 (#8242, Cell Signaling Technology, USA), IL-1β (sc-52012, Santa Cruz Biotechnology Inc., USA), IL-6 (sc-1265, Santa Cruz Biotechnology Inc., USA) or TNF-α (NBP1-19532, Novus Biologicals, USA). Secondary antibody (biotinylated IgG; Sigma-Aldrich, USA) was bound to the first antibody before washing and processing using Vectastain ABC-HRP kits (Vector Laboratories Inc., Burlingame, CA, USA) to show positive staining according to the manufacturer’s protocol.

***Immunofluorescent staining***

Staining was as previously described[^6^](#_ENREF_6)^,^ [^26^](#_ENREF_26). Primary antibodies were against GATA4 (#19530, Proteintech, USA), GATA4 (sc-25310, Santa Cruz Biotechnology Inc., USA), LC3B (#NB600-1384, Novus Biologicals, USA) p62 (#39749, Cell Signaling Technology, USA) or LAMP2 (sc-20004, Santa Cruz Biotechnology Inc., USA). Affinity-purified Alexa Fluor Dylight488-conjugated secondary antibody (goat anti-mouse IgG, GAM4882, Multi Sciences Biotech, Co., Ltd., Hangzhou, Zhejiang, China), Dylight594-conjugated secondary antibody (goat anti-rabbit IgG, GAR5942, Multi Sciences Biotech, Co., Ltd., China) and Dylight649-conjugated secondary antibody (goat anti-rat IgG, #A23640, Abbkine Scientific Co., Ltd., Wuhan, Hubei, China) were used. Nuclei were labeled with DAPI (Sigma-Aldrich, USA) and mounted with medium to prevent quenching (Vector Laboratories Inc., USA) as previously described[^6^](#_ENREF_6)^,^ [^7^](#_ENREF_7)^,^ [^26^](#_ENREF_26).

***Masson’s trichrome staining***

After deparaffinization and hydration, the paraffin sections were stained with hematoxylin for 10 min and rinsed with tap water, and dyed with composite staining solution for 5 min and rinsed with tap water (2-3 seconds to rinse off dye). One drop phosphomolybdic acid was added for 1 min, and shaken off while keeping the myocardial slices wet. One drop of bright green staining solution was added for 5 min and rinsed with tap water (2-3 seconds), dried and sealed with neutral balsam. All steps were according to instructions from the kit supplier (#KGMST-8003, KeyGen Biotech. Co. Ltd., Nanjing, Jiangsu, China)[^6^](#_ENREF_6).

***Wheat germ agglutinin staining (WGA)***

After paraffin sections were dewaxed and hydrated, WGA conjugated to Alexa Fluor 488 (MP00831, Molecular Probes Inc., OR, USA) was dissolved in PBS as 1.0 mg/mL stock solution according to the manufacturer. Stock solution was diluted 1:100 with PBS containing 0.3% Triton before incubation at 37°C for 4 h in a constant temperature incubator, and 4°C overnight[^13^](#_ENREF_13). Paraffin sections were processed to capture green fluorescence images after washing three times for 5 min with PBS.

***SA-β-gal staining***

Cells were seeded in 6-well plates and treatment with Ang II (5 × 10^-6^ mol/L), metformin (2.5 mM for 24 h) or rapamycin (100 nM for 24 h) at a density of 70–80%. Cells were fixed and stained using a cell senescence β-galactosidase staining kit (#C0602, Beyotime Institute of Biotechnology, Shanghai, China) according to the manufacturer’s instructions[^6^](#_ENREF_6).

**Autophagy detection lentivirus**

***Autophagy lentivirus infection***

Autophagy lentivirus (#HB-LP210 0001) was from HanBio Technology Co., Ltd. In Shanghai of China. Its fusion protein elements included mCherry red fluorescent protein (mRFP) (λ-excitation 587 nm, λ-emission 610 nm), green fluorescent protein (GFP) (λ-excitation 488 nm, λ-emission 507 nm) and autophagy marker protein LC3B (mRFP-GFP-LC3) to monitor formation of autophagosomes and autolysosomes as previously described[^27^](#_ENREF_27).

Viral inoculum containing 1 × 10^8^ Transduction Units (TU)/ml was diluted with OPTI-MEM into MOI = 100 to transfect into MECs or AC16 cells with incubation with virus solution for 24 h in a humidified 5% CO_2_ incubator at 37°C. Medium was replaced with conventional medium after 24 h and culturing continued for 40–48 h. Cells were fixed with PLP and stained with DAPI.

***Overexpression and knockdown lentivirus infection***

The *Bmi-1* overexpression lentivirus and knockdown lentivirus were designed and synthesized by Genechem Co., Ltd. in Shanghai of China. Lentivirus was diluted with OPTI-MEM to MOI = 10 to transfect AC16 cells in 24-well plates. Incubation was with OPTI-MEM for 24 h in a humidified 5% CO_2_ incubator at 37°C. Medium was replaced with α-MEM with 10% FBS after 24 h and culturing continued for 40–48 h in a humidified 5% CO_2_ incubator at 37°C. Selective culturing with puromycin dihydrochloride (#ST551, Beyotime Institute of Biotechnology, Shanghai, China) obtained transfected cells. Cells were treated with Ang II for Western blots.

**Intracellular reactive oxygen species analysis**

To analyze intracellular reactive oxygen species (ROS) levels, hearts from six-week-old mice were dissected and digested to a single-cell suspension and labeled with 5 mM 2’, 7’-Dichlorofluorescein diacetates (DCFDA) (D399, Invitrogen Inc., USA) in the dark for 30 min in a 37°C water bath. Cells were detected with a FACS caliber ﬂow cytometer (Becton Dickinson, Heidelberg, Germany)[^21^](#_ENREF_21)^,^ [^23^](#_ENREF_23).

**Enzyme-linked immunosorbent assay**

Enzyme-linked immunosorbent assay **(**ELISA) kits (Yifeixue Biotechnology, Nanjing, China) were used to detect concentrations of human-derived Bmi-1 (#H00115), RING1B/RNF2 (#H00515), p16 (#H01273), GATA4 (#H00555), ANP (#H0470), p62/SQSTM1 (#H00597) and LC3B (#H00815) in human myocardial tissue as previously described [^6^](#_ENREF_6).

**Protein sequence alignment**

The amino acid sequences of GATA3, GATA4 and GATA6 protein from mouse or human were aligned using online tools (Uniprot/Align: <https://www.uniprot.org/align/>) (SI6-7 Alignment of GATA3, GATA4 and GATA6 in Human or Mouse).

The amino acid sequences of GATA4 protein and motif “KFERQ**”** from mouse or human were aligned using online tools (Uniprot/Align: <https://www.uniprot.org/align/>) (SI8 Alignment of GATA4 protein and motif- KFERQ in Human or Mouse).

**Duolink Proximity Ligation Assay**

AC16 cells were treated with Ang II, then fixed and mixed with antibodies against GATA4 ([sc-25310](https://www.scbt.com/zh/p/gata-4-antibody-g-4?requestFrom=search), Santa Cruz Biotechnology Inc., USA), Bmi-1 (#5856, Cell Signaling Technology, USA), Bmi-1 (AM1930b, ABCEPTA Inc., CA, USA), RING1B (#5694, Cell Signaling Technology, USA) or HSC70 (10654-1-AP, Proteintech, USA). PLA *in situ* fluorescence (Sigma-Aldrich, USA) was performed according to the manufacturer’s instructions with Duolink *in situ* probe anti-mouse PLUS (#DUO92001), Duolink in situ PLA probe anti-rabbit MINUS (#DUO92005), Duolink *in situ* detection reagents Red (#DUO92008) and Duolink *in situ* wash buffers-fluorescence (#DUO82049). The PLA signal (λex 594 nm, λem 624 nm; Texas Red) was analyzed[^6^](#_ENREF_6).

**Plasmid construction and transfection of truncated and full-length fragments**

Based on the structural features of Bmi-1, we generated three truncated fragments containing a RING-HC-finger, WD40-associated ubiquitin-like (RAWUL) domain and carboxy-terminal domain of human Bmi-1, all with a His-tag in pmCherry-C1 vector plasmid. We generated a *Bmi-1* full-length overexpression plasmid carrying a His-tag in the pcDNA3.1 plasmid.

GATA4 is composed of an amino-terminal GATA-type transcription activator domain and a conserved carboxy-terminal zinc finger DNA-binding domain. We generated two truncated fragments and a full-length *GATA4* carrying a Flag-tag in a PEGFP-C1 vector.

RING1B was divided into an amino-terminal conserved protein containing a RING Zn-finger, a carboxy-terminal RAWUL domain, two truncated fragments and a full-length *RING1B*. All had an HA-tag in the PEGFP-C1 vector.

*Ubiquitin* full-length overexpression plasmid was generated carrying a Myc-tag in the pcDNA3.1 vector.

Production of plasmids was by TranSheep Bio Co. Ltd, Shanghai, China. Plasmids were co-transfected into 293T cells at 60–70% confluence using Lipofectamine 2000 (#11668-019, Invitrogen Inc., USA). The 293T cells were incubated in complete medium after transfection mixture for 6 h, and were harvested after 48 h. We followed standard protocols for Lipofectamine 2000.

**Protein immunoprecipitation**

Total proteins extracted from animal heart tissues, MECs or 293T cells transfected with plasmids were used for immunoprecipitation using Pierce™ Crosslink Magnetic IP/Co-IP kits (#88805, Thermo Scientific Pierce™ Crosslink Magnetic IP/Co-IP Kit, Thermo Fisher Scientific, IL, USA) as recommended by the supplier. Proteins were mixed with 5 μg antibody and prewashed protein A/G and incubated overnight with anti-IgG antibody as a control. Bound antigens were eluted from beads by boiling for 10 min. Eluted samples were used for SDS-PAGE. Immunoblotting was as previously described[^28^](#_ENREF_28), Clean-Blot™ IP Detection Reagent (HRP, horseradish peroxidase) (#21230, Thermo Scientific, USA) was used as second antibody to eliminate IgG bands. Primary antibodies against Bmi-1 (#5856, Cell Signaling Technology, USA), RING1B (#5694, Cell Signaling Technology), GATA4 (#19530, Proteintech, USA), p62 (#39749, Cell Signaling Technology, USA), anti-Ubiquitin (#91112, Cell Signaling Technology, USA), HSC70 (10654-1-AP, Proteintech, USA), DYKDDDDK Tag (binds the same epitope as Sigma's Anti-FLAG M2 antibody, #14793, Cell Signaling Technology, USA), His-Tag (#12698, Cell Signaling Technology, USA) and HA-Tag (#3724, Cell Signaling Technology, USA) were used. Immunoreactive bands were visualized by ECL chemiluminescence (Amersham Pharmacia Biotech, NJ, USA) and analyzed by a ChemiDoc™ XRS+ System with Image Lab™ Software (Bio-Rad laboratories Inc., CA, USA).

**Protein stability**

*Bmi-1* overexpressed AC16 cells or controlled AC16 cells were planted into 10-cm plates, and treated with Ang II (1 × 10^-5^ for 70 h) to induce hypertrophy. Cells were cultured for 12 h in the presence of 5 μM^[6](#_ENREF_6)^ MG132 (#474787, Sigma-Aldrich, USA) or 100 nM^[29](#_ENREF_29)^ Bafilomycin A1(Baf-A1, #S1413, Sigma-Aldrich, USA), then treated with 100 μM^[30](#_ENREF_30)^ Cycloheximide (CHX, #5.08739, Sigma-Aldrich, USA) plus 5 μM^[6](#_ENREF_6)^ MG132, or CHX plus 100 nM^[29](#_ENREF_29)^ Baf-A1 for indicated times. Then proteins were extracted to detect the target proteins with Western blots.

**Ubiquitination assay**

As previously described method[^30^](#_ENREF_30), the 293T cells were transfected with plasmids including Myc-Ubiquitin, Flag-GATA4, HA-RING1B and/or His-Bmi-1, and lysed in the presence of deubiquitination inhibitor N-Ethylmaleimide (E3876, Sigma-Aldrich, USA), sonicated and subjected to immunoprecipitation with anti-DYKDDDDK Tag (binds the same epitope as Sigma's Anti-FLAG M2 antibody, #14793, Cell Signaling Technology, USA) antibody. The levels of ubiquitination were subsequently detected by Western blot with anti-Ubiquitin (#91112, Cell Signaling Technology, USA) antibody.

**Adeno-Associated Virus carrying *Bmi-1-RING1B***

Serotype 9 adeno-associated virus (AAV9) carrying the specific sequence that overexpressing Bmi-1-RING1B or the corresponding control virus was designed and produced (HanBio Technology Co., Ltd., China). Previous reports indicated that AAV9- *cytomegalovirus (CMV)* viral packaging was an efficient and safe tool for cardiac gene transfer[^31-33^](#_ENREF_31). Furthermore, the *CMV* promoter and its derivatives have been used in multiple preclinical studies and even in the first-in-human cardiac gene therapy trial because of its robust expression[^34^](#_ENREF_34)^,^ [^35^](#_ENREF_35). In this study, the complex *Bmi-1* (975 bp)–*RING1B* (1, 011 bp) was needed to constructed into AAV9. Because of the large base number of *Bmi-1–RING1B* complex, we used a universal promoter *CMV* to better express *Bmi-1–RING1B* complex. Mice were injected with 100μl AAV9-*CMV*-*Bmi-1-RING1B* (1×10^12^ v.g/ml) by caudal vein. After 2 weeks of AAV9-*CMV*-*Bmi-1-RING1B* treatment, cardiac hypertrophy was induced by chronic subcutaneous infusion of angiotensin II (Ang II, #A9525, Sigma-Aldrich, USA) at a dose of 1.3 mg/kg/day using the ALZET® Micro-Osmotic Pumps (Model 1004) (#10370-16, DURECT Corporation, CA, USA) for 4 weeks.

**Statistical analysis**

As previously described, all analyses used GraphPad Prism software (Version 6.07; GraphPad Software Inc., San Diego, CA, USA)[^36^](#_ENREF_36). Measurement data were described as mean ± SEM fold-change over a control group and analyzed by Student’s *t*-test and one-way ANOVA to compare the differences among the groups[^6^](#_ENREF_6). Qualitative data were shown as percentages and analyzed using chi-squared tests as indicated[^6^](#_ENREF_6)^,^ [^7^](#_ENREF_7)^,^ [^20^](#_ENREF_20). Correlations of Gaussian-distributed data were analyzed by Pearson’s *r*; non-Gaussian distributed data were analyzed by Spearman’s rank correlation coefficient[^6^](#_ENREF_6). P-values were two-sided and values less than 0.05 were considered statistically significant[^6^](#_ENREF_6)^,^ [^7^](#_ENREF_7)^,^ [^20^](#_ENREF_20).

**References**

1. Zhang HW, Ding J, Jin JL*, et al.* Defects in mesenchymal stem cell self-renewal and cell fate determination lead to an osteopenic phenotype in Bmi-1 null mice. *J Bone Miner Res* 2010; **25**: 640-652.

2. Higuchi M, Kato T, Chen M*, et al.* Temporospatial gene expression of Prx1 and Prx2 is involved in morphogenesis of cranial placode-derived tissues through epithelio-mesenchymal interaction during rat embryogenesis. *Cell Tissue Res* 2013; **353**: 27-40.

3. Chen G, Zhang Y, Yu S, Sun W, Miao D. Bmi1 Overexpression in Mesenchymal Stem Cells Exerts Antiaging and Antiosteoporosis Effects by Inactivating p16/p19 Signaling and Inhibiting Oxidative Stress. *Stem Cells* 2019; **37**: 1200-1211.

4. Chen G, Zhang Y, Yu S, Sun W, Miao D. Bmi1 Overexpression in Mesenchymal Stem Cells Exerts Antiaging and Antiosteoporosis Effects by Inactivating p16/p19 Signaling and Inhibiting Oxidative Stress. *Stem cells (Dayton, Ohio)* 2019; **37**: 1200-1211.

5. Sun H, Qiao W, Cui M*, et al.* The Polycomb Protein Bmi1 Plays a Crucial Role in the Prevention of 1,25(OH)2 D Deficiency-Induced Bone Loss. *J Bone Miner Res* 2019.

6. H C, H C, J L*, et al.* TGF-β1/IL-11/MEK/ERK signaling mediates senescence-associated pulmonary fibrosis in a stress-induced premature senescence model of Bmi-1 deficiency. *Experimental & molecular medicine* 2020; **52**: 130-151.

7. Jin J, Tao J, Gu X*, et al.* P16 (INK4a) Deletion Ameliorated Renal Tubulointerstitial Injury in a Stress-induced Premature Senescence Model of Bmi-1 Deficiency. *Sci Rep* 2017; **7**: 7502.

8. Palomer X, Alvarez-Guardia D, Rodriguez-Calvo R*, et al.* TNF-alpha reduces PGC-1 alpha expression through NF-kappa B and p38 MAPK leading to increased glucose oxidation in a human cardiac cell model. *Cardiovasc Res* 2009; **81**: 703-712.

9. Davidson MM, Nesti C, Palenzuela L*, et al.* Novel cell lines derived from adult human ventricular cardiomyocytes. *Journal of molecular and cellular cardiology* 2005; **39**: 133-147.

10. Mahmoodzadeh S, Pham TH, Kuehne A*, et al.* 17beta-Estradiol-induced interaction of ERalpha with NPPA regulates gene expression in cardiomyocytes. *Cardiovasc Res* 2012; **96**: 411-421.

11. Zhao Q, Song W, Huang J, Wang D, Xu C. Metformin decreased myocardial fibrosis and apoptosis in hyperhomocysteinemia -induced cardiac hypertrophy. *Current research in translational medicine* 2021; **69**: 103270.

12. Palomer X, Roman-Azcona MS, Pizarro-Delgado J*, et al.* SIRT3-mediated inhibition of FOS through histone H3 deacetylation prevents cardiac fibrosis and inflammation. *Signal transduction and targeted therapy* 2020; **5**: 14.

13. X T, XF C, NY W*, et al.* SIRT2 Acts as a Cardioprotective Deacetylase in Pathological Cardiac Hypertrophy. *Circulation* 2017; **136**: 2051-2067.

14. Shoieb SM, El-Kadi AOS. S-Enantiomer of 19-Hydroxyeicosatetraenoic Acid Preferentially Protects Against Angiotensin II-Induced Cardiac Hypertrophy. *Drug Metab Dispos* 2018; **46**: 1157-1168.

15. Novelle MG, Ali A, Dieguez C, Bernier M, de Cabo R. Metformin: A Hopeful Promise in Aging Research. *Cold Spring Harbor Perspectives in Medicine* 2016; **6**.

16. Dou XW, Sun Y, Li JZ*, et al.* Short-term rapamycin treatment increases ovarian lifespan in young and middle-aged female mice. *Aging Cell* 2017; **16**: 825-836.

17. Moiseeva O, Deschenes-Simard X, St-Germain E*, et al.* Metformin inhibits the senescence-associated secretory phenotype by interfering with IKK/NF-B activation. *Aging Cell* 2013; **12**: 489-498.

18. Dhingra R, Gang HY, Wang Y*, et al.* Bidirectional Regulation of Nuclear Factor-kappa B and Mammalian Target of Rapamycin Signaling Functionally Links Bnip3 Gene Repression and Cell Survival of Ventricular Myocytes. *Circulation-Heart Failure* 2013; **6**: 335-343.

19. Egan DF, Chun MG, Vamos M*, et al.* Small Molecule Inhibition of the Autophagy Kinase ULK1 and Identification of ULK1 Substrates. *Molecular cell* 2015; **59**: 285-297.

20. Jin J, Lv X, Chen L*, et al.* Bmi-1 plays a critical role in protection from renal tubulointerstitial injury by maintaining redox balance. *Aging cell* 2014; **13**: 797-809.

21. Liu J, Cao L, Chen J*, et al.* Bmi1 regulates mitochondrial function and the DNA damage response pathway. *Nature* 2009; **459**: 387-392.

22. Jin JL, Zhao YM, Tan X, Guo C, Yang ZJ, Miao DS. An Improved Transplantation Strategy for Mouse Mesenchymal Stem Cells in an Acute Myocardial Infarction Model. *PloS one* 2011; **6**.

23. J J, X L, L C*, et al.* Bmi-1 plays a critical role in protection from renal tubulointerstitial injury by maintaining redox balance. *Aging cell* 2014; **13**: 797-809.

24. Dai X, Zhang Q, Yu Z, Sun W, Wang R, Miao D. Bmi1 Deficient Mice Exhibit Male Infertility. *Int J Biol Sci* 2018; **14**: 358-368.

25. Jin J, Zhao Y, Tan X, Guo C, Yang Z, Miao D. An improved transplantation strategy for mouse mesenchymal stem cells in an acute myocardial infarction model. *PloS one* 2011; **6**: e21005.

26. Xie C, Jin J, Lv X, Tao J, Wang R, Miao D. Anti-aging Effect of Transplanted Amniotic Membrane Mesenchymal Stem Cells in a Premature Aging Model of Bmi-1 Deficiency. *Sci Rep* 2015; **5**: 13975.

27. Yu T, Guo F, Yu Y*, et al.* Fusobacterium nucleatum Promotes Chemoresistance to Colorectal Cancer by Modulating Autophagy. *Cell* 2017; **170**: 548-563 e516.

28. Chen L, Yang R, Qiao W*, et al.* 1,25-Dihydroxy vitamin D prevents tumorigenesis by inhibiting oxidative stress and inducing tumor cellular senescence in mice. *Int J Cancer* 2018; **143**: 368-382.

29. Zhang H, Zhang Y, Zhu X*, et al.* DEAD Box Protein 5 Inhibits Liver Tumorigenesis by Stimulating Autophagy via Interaction with p62/SQSTM1. *Hepatology* 2019; **69**: 1046-1063.

30. Lavial F, Bessonnard S, Ohnishi Y*, et al.* Bmi1 facilitates primitive endoderm formation by stabilizing Gata6 during early mouse development. *Genes Dev* 2012; **26**: 1445-1458.

31. Chen BD, He CH, Chen XC*, et al.* Targeting transgene to the heart and liver with AAV9 by different promoters. *Clinical and experimental pharmacology & physiology* 2015; **42**: 1108-1117.

32. Liu Y, Zhou K, Li J*, et al.* In Mice Subjected to Chronic Stress, Exogenous cBIN1 Preserves Calcium-Handling Machinery and Cardiac Function. *JACC Basic to translational science* 2020; **5**: 561-578.

33. Zhao Q, Liu F, Zhao Q*, et al.* Constitutive activation of ERK1/2 signaling protects against myocardial ischemia via inhibition of mitochondrial fragmentation in the aging heart. *Annals of translational medicine* 2021; **9**: 479.

34. Jessup M, Greenberg B, Mancini D*, et al.* Calcium Upregulation by Percutaneous Administration of Gene Therapy in Cardiac Disease (CUPID): a phase 2 trial of intracoronary gene therapy of sarcoplasmic reticulum Ca2+-ATPase in patients with advanced heart failure. *Circulation* 2011; **124**: 304-313.

35. Bezzerides VJ, Prondzynski M, Carrier L, Pu WT. Gene therapy for inherited arrhythmias. *Cardiovasc Res* 2020; **116**: 1635-1650.

36. Schafer S, Viswanathan S, Widjaja AA*, et al.* IL-11 is a crucial determinant of cardiovascular fibrosis. *Nature* 2017; **552**: 110-115.
