# Supplementary material for: Bmi‐1‐RING1B prevents GATA4‐dependent senescence‐associated pathological cardiac hypertrophy by promoting autophagic degradation of GATA4
Source: Clin Transl Med. 2022 Apr 7;12(4):e574. doi: 10.1002/ctm2.574 (PMC8989148; doi:10.1002/ctm2.574)
Supplement: Supplementary file 4 — Supplementary Information 5: Table S1 [file CTM2-12-e574-s008.docx]

**Table S1︱Primers for Real Time RT-PCR**

| Name | S/AS | Sequence | Primer(bp) | Species | Tm  (°C) | Length  (bp) |
| --- | --- | --- | --- | --- | --- | --- |
| *AT1* | S | 5’-TGCCATGCCCATAACC-3’ | 16 | mouse | 55 | 147 |
|  | AS | 5’-GACAGGCTTGAGTGCGACTTG-3’ | 21 |  |  |  |
| *AT2* | S | 5’-ATGCTTGTATTATGGCTTTCCCA-3’ | 23 | mouse | 60 | 182 |
|  | AS | 5’-TTGGTCACGGGTAATTCTGTTC-3’ | 22 |  |  |  |
| *Renin* | S | 5’-ATCCTTTATCTCGGCTCCTAC-3’ | 21 | mouse | 55 | 108 |
|  | AS | 5’-GTGGGCACCTGGCTACAGTTC-3’ | 21 |  |  |  |
| *Aogen* | S | 5’-CCTCTTCCCACGCTCTCTG-3’ | 19 | mouse | 55 | 108 |
|  | AS | 5’-TCCAGTGGCAAGTTCATCTTC-3’ | 21 |  |  |  |
| *P16* | S | 5’-CCCGATTCAGGTGATGATGAT-3’ | 21 | mouse | 55 | 100 |
|  | AS | 5’-GCGGGAGAAGGTAGTGG-3’ | 17 |  |  |  |
| *P19* | S | 5’-GGCGACGTGCAAGAGGTCC-3’ | 19 | mouse | 60 | 258 |
|  | AS | 5’-GTCCAGGGCATTGACATCAGC-3’ | 21 |  |  |  |
| *ANP* | S | 5’-ACCTGCTAGACCACCTGGAG-3’ | 20 | mouse | 60 | 347 |
|  | AS | 5’-CCTTGGCTGTTATCTTCGGTACCGG-3’ | 25 |  |  |  |
| *BNP* | S | 5’-GAGGTCACTCCTATCCTCTGG-3’ | 21 | mouse | 58 | 100 |
|  | AS | 5’-GCCATTTCCTCCGACTTTTCTC-3’ | 22 |  |  |  |
| *β-MHC* | S | 5’-CCGAGTCCCAGGTCAACAA-3’ | 19 | mouse | 60 | 107 |
|  | AS | 5’-CTTCACGGGCACCCTTGGA-3’ | 19 |  |  |  |
| *Acta1* | S | 5’-CCAAAGCTAACCGGGAGAAG-3’ | 20 | mouse | 58 | 88 |
|  | AS | 5’-GACAGCACCGCCTGGATAG-3’ | 19 |  |  |  |
| *Acta2* | S | 5’-GTCCCAGACATCAGGGAGTAA-3’ | 21 | mouse | 60 | 102 |
|  | AS | 5’-TCGGATACTTCAGCGTCAGGA-3’ | 21 |  |  |  |
| *Rcan1.4* | S | 5’-TTGTGTGGCAAACGATGATGT-3’ | 21 | mouse | 55 | 189 |
|  | AS | 5’-CCCAGGAACTCGGTCTTGT-3’ | 19 |  |  |  |
| *p65 (RelA)* | S | 5’-AGGCTTCTGGGCCTTATGTG-3’ | 20 | mouse | 56 | 111 |
|  | AS | 5’-TGCTTCTCTCGCCAGGAATAC-3’ | 21 |  |  |  |
| *TRAF3IP2* | S | 5’-TCCCGTGGAGGTTGATGAATC-3’ | 21 | mouse | 55 | 249 |
|  | AS | 5’-TCAGGGTGCCTTCTAAAGAAACT-3’ | 23 |  |  |  |
| *β-Actin* | S | 5’-GGCTGTATTCCCCTCCATCG-3’ | 20 | mouse | 60 | 154 |
|  | AS | 5’-CCAGTTGGTAACAATGCCATGT-3’ | 22 |  |  |  |

S, sense; AS, antisense, sequence; Tm, annealing temperature; length, amplicon
