# Supplementary material for: Bmi‐1‐RING1B prevents GATA4‐dependent senescence‐associated pathological cardiac hypertrophy by promoting autophagic degradation of GATA4
Source: Clin Transl Med. 2022 Apr 7;12(4):e574. doi: 10.1002/ctm2.574 (PMC8989148; doi:10.1002/ctm2.574)
Supplement: Supplementary file 5 — Supplementary Information 6: Alignment of GATA3, GATA4 and GATA6 in Human [file CTM2-12-e574-s009.docx]

**Alignment of GATA3, GATA4 and GATA6 in human**


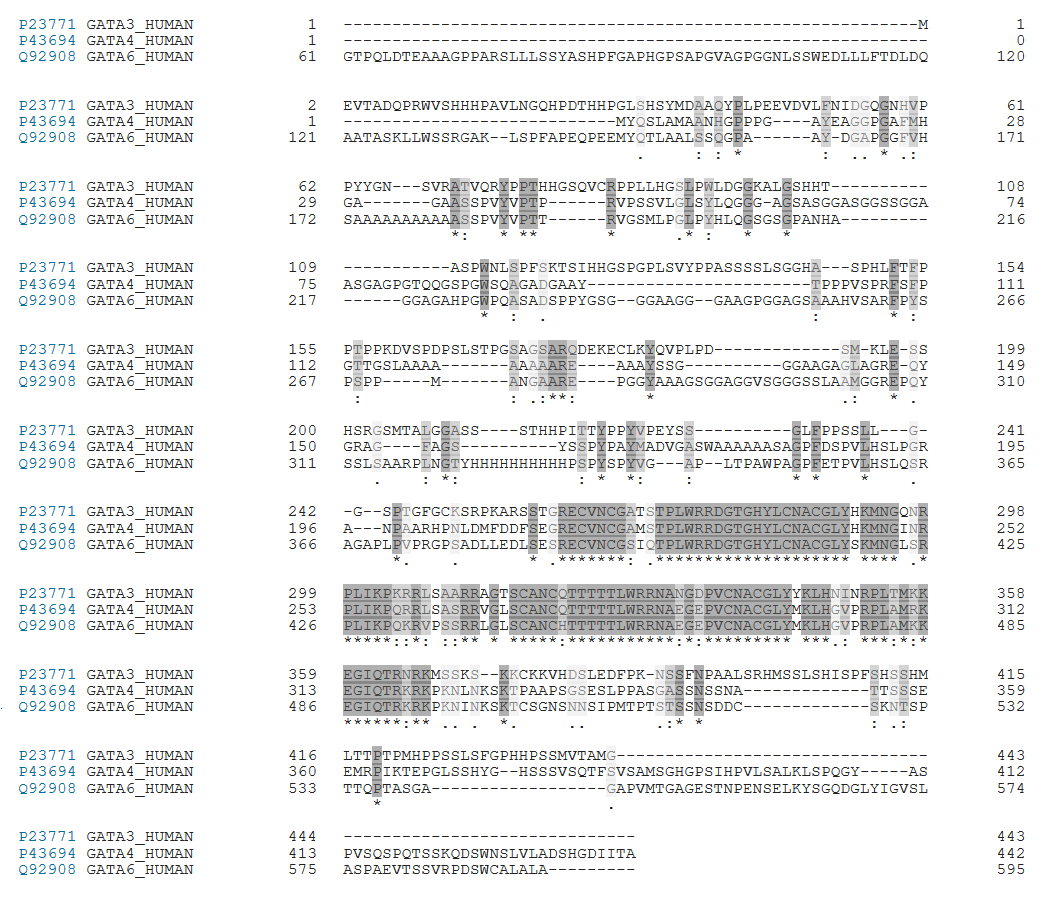


“*” indicating a single and fully conserved residue, “:” indicating residue with very similar properties, “.” indicating residue that is weakly similar.
