# Supplementary material for: Bmi‐1‐RING1B prevents GATA4‐dependent senescence‐associated pathological cardiac hypertrophy by promoting autophagic degradation of GATA4
Source: Clin Transl Med. 2022 Apr 7;12(4):e574. doi: 10.1002/ctm2.574 (PMC8989148; doi:10.1002/ctm2.574)
Supplement: Supplementary file 7 — Supplementary Information 8: Alignment of GATA4 Protein and motif‐KFERQ in Human or Mouse [file CTM2-12-e574-s004.doc]

**SI8 Alignment of GATA4 protein and motif- KFERQ in Human or Mouse**

1. **GATA4-human**

GATA4 MYQSLAMAANHGPPPGAYEAGGPGAFMHGAGAASSPVYVPTPRVPSSVLGLSYLQGGGAG

------------------------------------------------------------

GATA4 SASGGASGGSSGGAASGAGPGTQQGSPGWSQAGADGAAYTPPPVSPRFSFPGTTGSLAAA

------------------------------------------------------------

GATA4 AAAAAAREAAAYSSGGGAAGAGLAGREQYGRAGFAGSYSSPYPAYMADVGASWAAAAAAS

------------------------------------------------------------

GATA4 AGPFDSPVLHSLPGRANPAARHPNLDMFDDFSEGRECVNCGAMSTPLWRRDGTGHYLCNA

------------------------------------------------------------

GATA4 CGLYHKMNGINRPLIKPQRRLSASRRVGLSCANCQTTTTTLWRRNAEGEPVCNACGLYMK

---------------KFERQ----------------------------------------

* :*:

GATA4 LHGVPRPLAMRKEGIQTRKRKPKNLNKSKTPAAPSGSESLPPASGASSNSSNATTSSSEE

------------------------------------------------------------

GATA4 MRPIKTEPGLSSHYGHSSSVSQTFSVSAMSGHGPSIHPVLSALKLSPQGYASPVSQSPQT

------------------------------------------------------------

GATA4 SSKQDSWNSLVLADSHGDIITA

----------------------

1. **GATA4-mouse**

GATA4 MYQSLAMAANHGPPPGAYEAGGPGAFMHSAGAASSPVYVPTPRVPSSVLGLSYLQGGGSA

------------------------------------------------------------

GATA4 AAAGTTSGGSSGAGPSGAGPGTQQGSPGWSQAGAEGAAYTPPPVSPRFSFPGTTGSLAAA

------------------------------------------------------------

GATA4 AAAAAAREAAAYGSGGGAAGAGLAGREQYGRPGFAGSYSSPYPAYMADVGASWAAAAAAS

------------------------------------------------------------

GATA4 AGPFDSPVLHSLPGRANPGRHPNLDMFDDFSEGRECVNCGAMSTPLWRRDGTGHYLCNAC

------------------------------------------------------------

GATA4 GLYHKMNGINRPLIKPQRRLSASRRVGLSCANCQTTTTTLWRRNAEGEPVCNACGLYMKL

--------------KFERQ-----------------------------------------

* :*:

GATA4 HGVPRPLAMRKEGIQTRKRKPKNLNKSKTPAGPAGETLPPSSGASSGNSSNATSSSSSSE

------------------------------------------------------------

GATA4 EMRPIKTEPGLSSHYGHSSSMSQTFSTVSGHGPSIHPVLSALKLSPQGYASPVTQTSQAS

------------------------------------------------------------

GATA4 SKQDSWNSLVLADSHGDIITA

---------------------

“*” indicating a single and fully conserved residue, “:” indicating residue with very similar properties.
