# Supplementary material for: Bmi‐1‐RING1B prevents GATA4‐dependent senescence‐associated pathological cardiac hypertrophy by promoting autophagic degradation of GATA4
Source: Clin Transl Med. 2022 Apr 7;12(4):e574. doi: 10.1002/ctm2.574 (PMC8989148; doi:10.1002/ctm2.574)
Supplement: Supplementary file 8 — Supplementary Information 9: Original Blots in Figures S14–S17 [file CTM2-12-e574-s007.pdf]

Figure S14

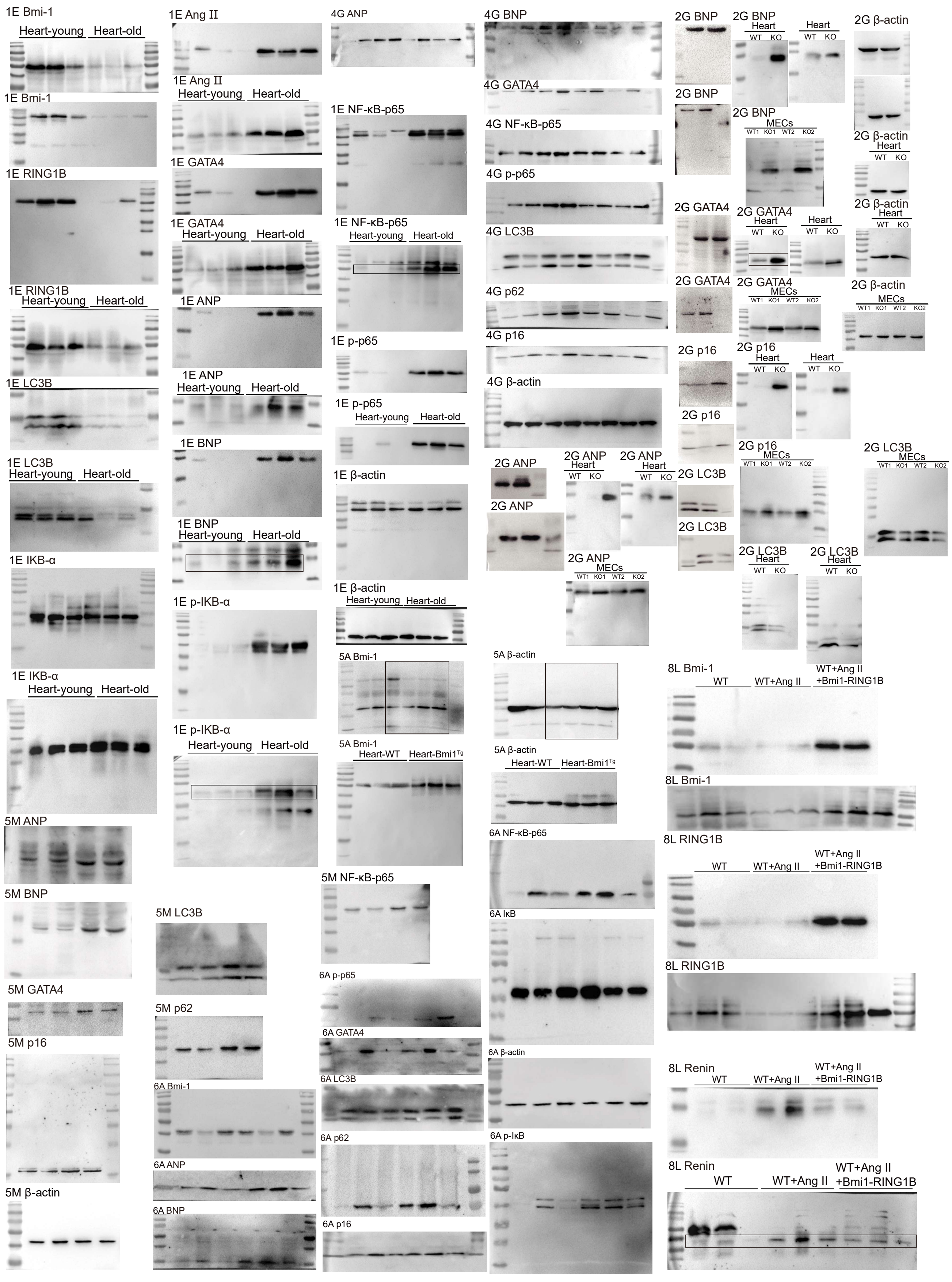
1E Bmi-1
Heart-young Heart-old
1E Bmi-1
1E RING1B
1E RING1B
Heart-young Heart-old
1E LC3B
1E LC3B
Heart-young Heart-old
1E IKB-α
1E IKB-α
Heart-young Heart-old
5M ANP
5M BNP
5M GATA4
5M p16
5M β-actin
1E Ang II
1E Ang II
Heart-young Heart-old
1E GATA4
1E GATA4
Heart-young Heart-old
1E ANP
1E ANP
Heart-young Heart-old
1E BNP
1E BNP
Heart-young Heart-old
1E p-IKB-α
1E p-IKB-α
Heart-young Heart-old
5M LC3B
5M p62
6A Bmi-1
6A ANP
6A BNP
4G ANP
1E NF-κB-p65
1E NF-κB-p65
Heart-young Heart-old
1E p-p65
1E p-p65
Heart-young Heart-old
1E β-actin
1E β-actin
Heart-young Heart-old
5A Bmi-1
5A Bmi-1
Heart-WT Heart-Bmi1Tg
5M NF-κB-p65
6A p-p65
6A GATA4
6A LC3B
6A p62
6A p16
4G BNP
4G GATA4
4G NF-κB-p65
4G p-p65
4G LC3B
4G p62
4G p16
4G β-actin
2G ANP
2G ANP
2G ANP
Heart
WT KO
2G ANP
Heart
WT KO
2G ANP
MECs
WT1 KO1 WT2 KO2
5A β-actin
5A β-actin
Heart-WT Heart-Bmi1Tg
6A NF-κB-p65
6A IκB
6A β-actin
6A p-IκB
2G BNP
2G BNP
2G GATA4
2G GATA4
2G p16
2G p16
2G LC3B
2G LC3B
2G BNP
Heart
WT KO
Heart
WT KO
2G BNP
MECs
WT1 KO1 WT2 KO2
2G GATA4
Heart
WT KO
Heart
WT KO
2G GATA4
MECs
WT1 KO1 WT2 KO2
2G p16
Heart
WT KO
Heart
WT KO
2G p16
MECs
WT1 KO1 WT2 KO2
2G LC3B
Heart
WT KO
2G LC3B
Heart
WT KO
2G β-actin
2G β-actin
Heart
WT KO
2G β-actin
Heart
WT KO
2G β-actin
MECs
WT1 KO1 WT2 KO2
2G LC3B
MECs
WT1 KO1 WT2 KO2
8L Bmi-1
WT WT+Ang II WT+Ang II +Bmi1-RING1B
8L Bmi-1
8L RING1B
WT WT+Ang II WT+Ang II +Bmi1-RING1B
8L RING1B
8L Renin
WT WT+Ang II WT+Ang II +Bmi1-RING1B
8L Renin
WT WT+Ang II WT+Ang II +Bmi1-RING1B

Figure S15

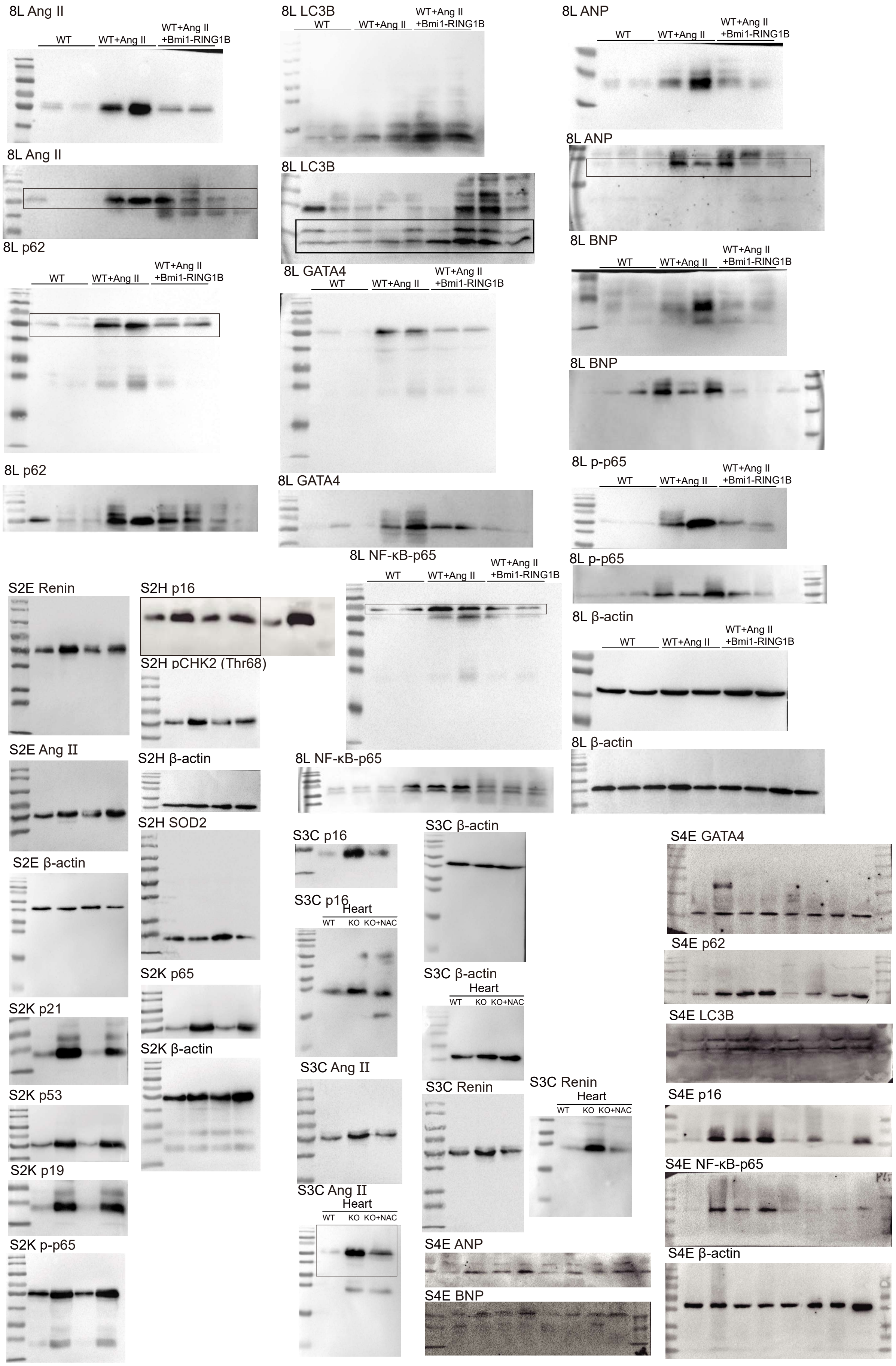

8L Ang II
WT
WT+Ang II
WT+Ang II +Bmi1-RING1B
8L Ang II
8L p62
WT
WT+Ang II
WT+Ang II +Bmi1-RING1B
8L p62
8L LC3B
WT
WT+Ang II
WT+Ang II +Bmi1-RING1B
8L LC3B
8L GATA4
WT
WT+Ang II
WT+Ang II +Bmi1-RING1B
8L GATA4
8L ANP
WT
WT+Ang II
WT+Ang II +Bmi1-RING1B
8L ANP
8L BNP
WT
WT+Ang II
WT+Ang II +Bmi1-RING1B
8L BNP
8L p-p65
WT
WT+Ang II
WT+Ang II +Bmi1-RING1B
8L p-p65
8L β-actin
WT
WT+Ang II
WT+Ang II +Bmi1-RING1B
8L β-actin
8L NF-κB-p65
WT
WT+Ang II
WT+Ang II +Bmi1-RING1B
8L NF-κB-p65
S2E Renin
S2E Ang II
S2E β-actin
S2H p16
S2H pCHK2 (Thr68)
S2H β-actin
S2H SOD2
S2K p21
S2K p53
S2K p19
S2K p-p65
S2K p65
S2K β-actin
S3C p16
S3C p16
Heart
WT KO KO+NAC
S3C Ang II
S3C Ang II
Heart
WT KO KO+NAC
S3C β-actin
S3C β-actin
Heart
WT KO KO+NAC
S3C Renin
S3C Renin
Heart
WT KO KO+NAC
S4E ANP
S4E BNP
S4E GATA4
S4E p62
S4E LC3B
S4E p16
S4E NF-κB-p65
S4E β-actin

Figure S16

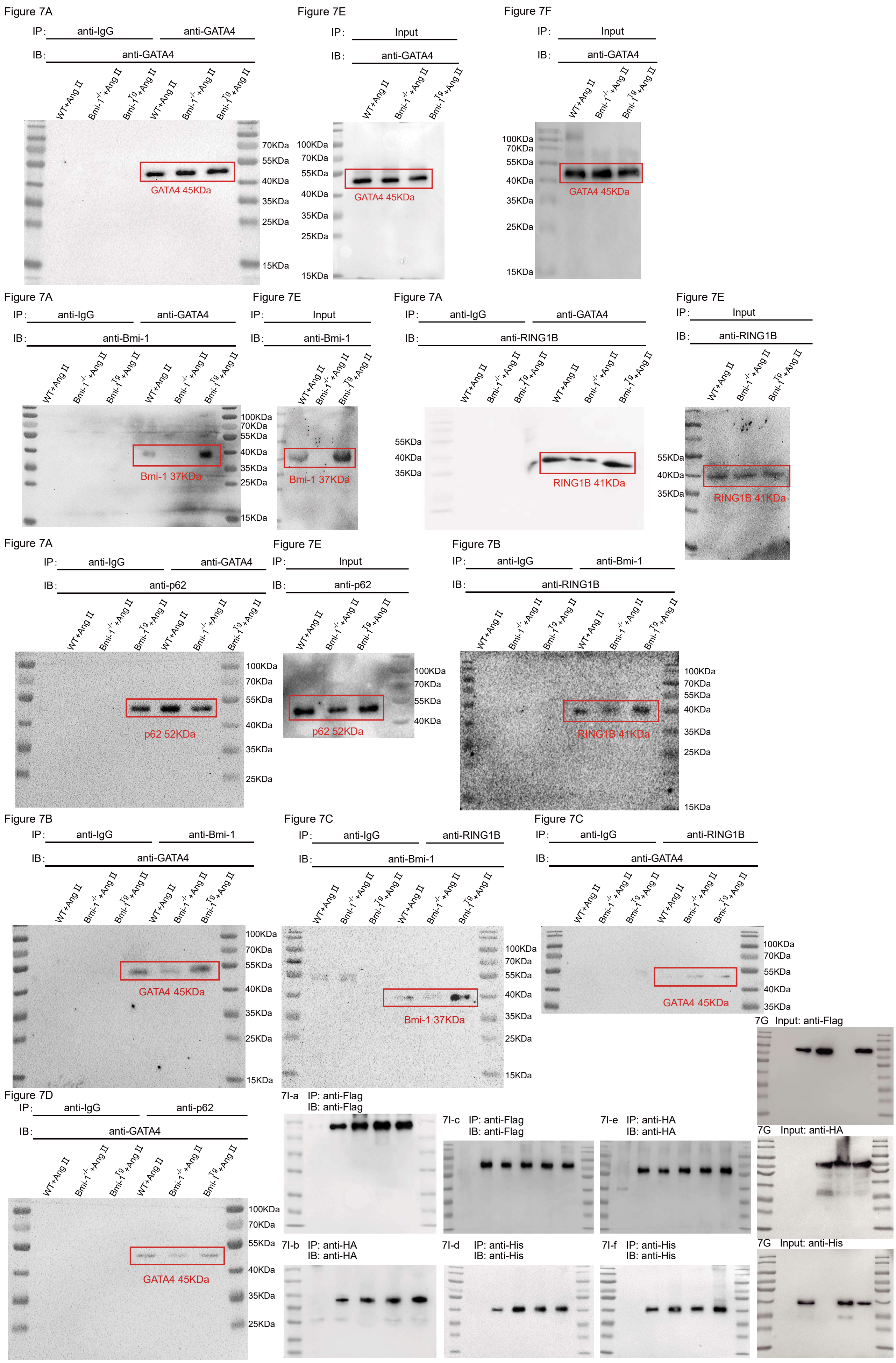
Figure 7A
IP: anti-IgG anti-GATA4
IB: anti-GATA4
WT+Ang II
Bmi-1-/-+Ang II
Bmi-1Tg+Ang II
70KDa
55KDa
40KDa
35KDa
25KDa
15KDa
GATA4 45KDa
Figure 7E
IP: Input
IB: anti-GATA4
100KDa
70KDa
55KDa
40KDa
35KDa
25KDa
15KDa
GATA4 45KDa
Figure 7F
IP: Input
IB: anti-GATA4
GATA4 45KDa
Figure 7A
IP: anti-IgG anti-GATA4
IB: anti-Bmi-1
Bmi-1 37KDa
Figure 7E
IP: Input
IB: anti-Bmi-1
Bmi-1 37KDa
Figure 7A
IP: anti-IgG anti-GATA4
IB: anti-RING1B
RING1B 41KDa
Figure 7E
IP: Input
IB: anti-RING1B
RING1B 41KDa
Figure 7A
IP: anti-IgG anti-GATA4
IB: anti-p62
p62 52KDa
Figure 7E
IP: Input
IB: anti-p62
p62 52KDa
Figure 7B
IP: anti-IgG anti-Bmi-1
IB: anti-RING1B
RING1B 41KDa
Figure 7B
IP: anti-IgG anti-Bmi-1
IB: anti-GATA4
GATA4 45KDa
Figure 7C
IP: anti-IgG anti-RING1B
IB: anti-Bmi-1
Bmi-1 37KDa
Figure 7C
IP: anti-IgG anti-RING1B
IB: anti-GATA4
GATA4 45KDa
7G Input: anti-Flag
Figure 7D
IP: anti-IgG anti-p62
IB: anti-GATA4
GATA4 45KDa
7I-a IP: anti-Flag
IB: anti-Flag
7I-c IP: anti-Flag
IB: anti-Flag
7I-e IP: anti-HA
IB: anti-HA
7G Input: anti-HA
7I-b IP: anti-HA
IB: anti-HA
7I-d IP: anti-His
IB: anti-His
7I-f IP: anti-His
IB: anti-His
7G Input: anti-His

Figure S17

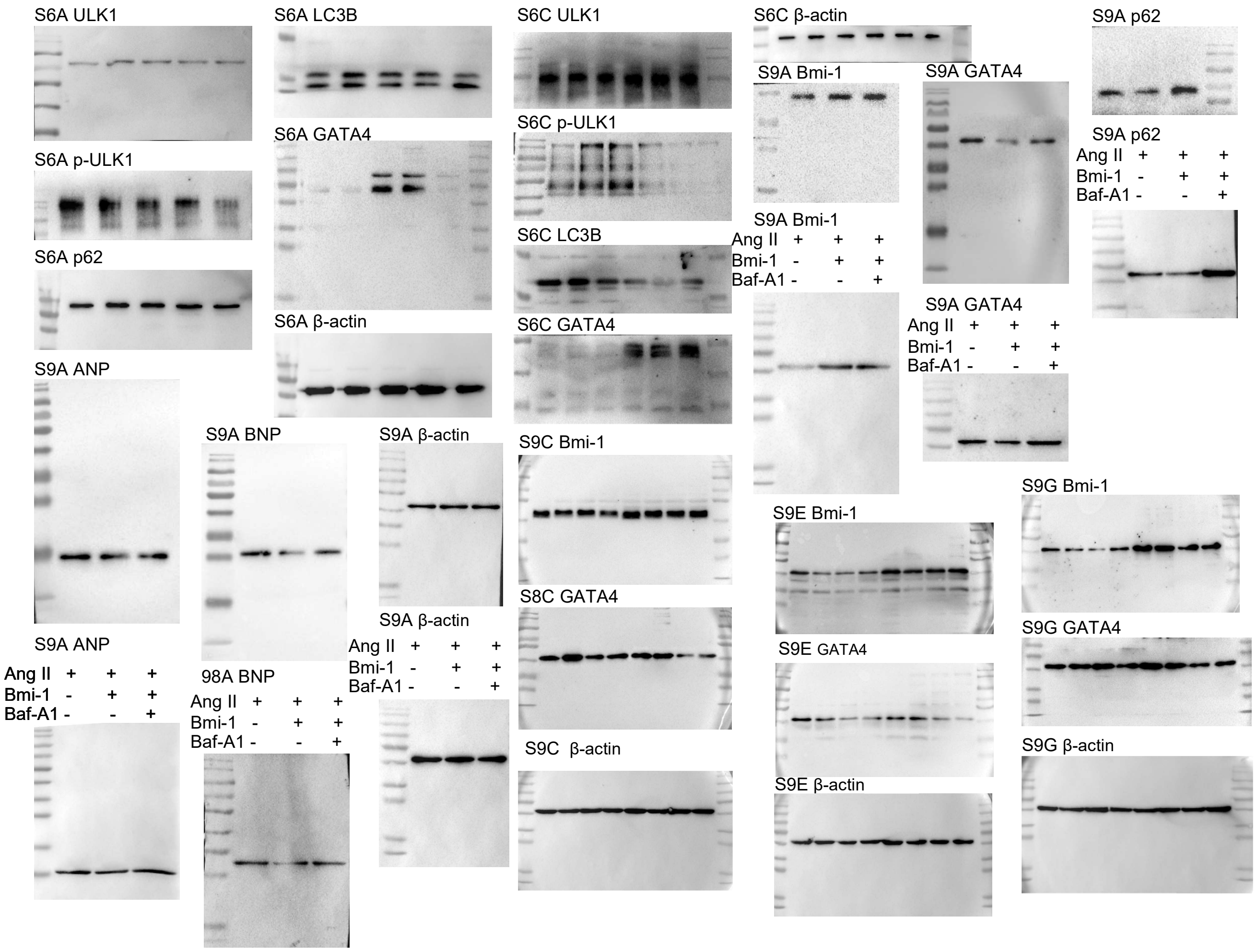

S6A ULK1
S6A LC3B
S6C ULK1
S6C β-actin
S9A p62
S9A Bmi-1
S9A GATA4
S6A GATA4
S6C p-ULK1
S9A p62
Ang II + + +
Bmi-1 - + +
Baf-A1 - - +
S6A p-ULK1
S9A Bmi-1
Ang II + + +
Bmi-1 - + +
Baf-A1 - - +
S6C LC3B
S6A p62
S9A GATA4
Ang II + + +
Bmi-1 - + +
Baf-A1 - - +
S6A β-actin
S6C GATA4
S9A ANP
S9A BNP
S9A β-actin
S9C Bmi-1
S9G Bmi-1
S9E Bmi-1
S8C GATA4
S9A β-actin
Ang II + + +
Bmi-1 - + +
Baf-A1 - - +
S9G GATA4
S9A ANP
Ang II + + +
Bmi-1 - + +
Baf-A1 - - +
S9E GATA4
98A BNP
Ang II + + +
Bmi-1 - + +
Baf-A1 - - +
S9C β-actin
S9G β-actin
S9E β-actin
